# Recent advances in antituberculosis drugs and therapeutics: from mechanistic breakthroughs to clinical translation

**DOI:** 10.1186/s43556-026-00610-5

**Published:** 2026-10-10

**Authors:** Zhixiang Pan, Guan Liu, Yuehua Li, Hegui Yan, Yu Peng, Yiyi Wang, Hongri Gong, Juan Du, Shuang Wei

**Affiliations:** 1 Wuhan Pulmonary Hospital (Wuhan Tuberculosis Prevention and Control Institute), Hubei Branch of National Clinical Research Center for Infectious Diseases, Wuhan, Hubei 430030 China; 2 State Key Laboratory of Medicinal Chemical Biology and College of Life Sciences, Nankai University, Tianjin, 300071 China; 3 Department of Respiratory and Critical Care Medicine, National Health Commission (NHC) Key Laboratory of Respiratory Disease, Tongji Hospital, Tongji Medical College, Huazhong University of Science and Technology, Wuhan, Hubei 430030 China

**Keywords:** *Mycobacterium tuberculosis*, Drug-resistant tuberculosis, Anti-TB drugs, Vaccine development, Global TB control

## Abstract

Tuberculosis (TB), caused by Mycobacterium tuberculosis, reclaimed its status as the deadliest single-pathogen infectious disease globally in 2023, accounting for 10.8 million new cases and 1.25 million deaths in 2023. The escalating crisis of drug-resistant TB, suboptimal BCG vaccine efficacy in adults, and persistent treatment gaps for vulnerable populations collectively drive an urgent shift toward mechanism-driven, precision-oriented therapeutic strategies.This review presents a critically integrated appraisal of anti-TB research advances spanning 2020 to 2025, drawing on structural biology, multi-omics, and real-world clinical evidence. It distinguishes itself from conventional descriptive literature compilations through systematic cross-population efficacy comparisons, in-depth dissection of translational bottlenecks, and explicit maturity stratification of emerging biotherapies. We synthesize progress across six interconnected domains: mechanistic insights into drug targets and spatiotemporal resistance evolution, novel short-course therapeutic regimens, precision medicine strategies, host-directed therapies, targeted delivery systems for extrapulmonary TB, and next-generation vaccine development. Three transformative breakthroughs are particularly highlighted: structural biology-guided rational drug design, spatial immune profiling of tuberculous granulomas, and AI-assisted diagnostic pipelines.We conclude by delineating priority research directions aligned with the WHO End TB Strategy, providing a translational roadmap to advance global TB elimination efforts. This rigorous, multi-dimensional synthesis offers an authoritative reference for advancing basic and translational anti-TB research and informing global public health strategy.

## Introduction

Tuberculosis remains a leading cause of mortality from a single infectious agent, with the World Health Organization estimating approximately 10 million new cases and 1.3 million deaths annually, positioning it as the second deadliest infectious disease globally after COVID-19 (2020–2022) [1]. The global burden is disproportionately concentrated in specific regions, with China ranking fourth among the highest-burden countries, reporting approximately 741,000 (2023) new TB cases each year [1].

Despite significant progress in control efforts over the past two decades, the path toward global TB elimination is obstructed by the escalating prevalence of drug-resistant strains, particularly multidrug-resistant TB (MDR-TB) and extensively drug-resistant TB (XDR-TB) [2]. These resistant forms are driven by both ongoing resistance acquisition during inadequate treatment and direct person-to-person transmission, exacerbating poor outcomes due to late diagnosis and limited access to effective regimens [2].The clinical management of MDR-TB and XDR-TB is notoriously difficult, historically resulting in devastating outcomes, although recent meta-analyses indicate that successful treatment outcomes are achievable with modern all-oral regimens [3]. In China, the prevalence and patterns of drug-resistant Mycobacterium tuberculosis remain a critical public health challenge, with systematic reviews highlighting the need for enhanced surveillance and targeted interventions in newly diagnosed patients [4]. The treatment landscape is further complicated by prolonged therapeutic courses and severe adverse effects associated with existing regimens, which hinder patient adherence and contribute to the persistence of the epidemic [5]. For instance, cohort studies in Eastern China have evaluated the effectiveness, cost, and safety of WHO-recommended regimens for rifampicin-resistant and MDR-TB, underscoring the economic and clinical burdens placed on healthcare systems [5]. Additionally, isoniazid-resistant, rifampicin-susceptible TB (Hr-TB) represents the most frequent form of drug-resistant TB globally, with unfavorable outcomes occurring more commonly than in drug-susceptible cases [6].Diagnostic challenges continue to plague TB control efforts, as accurate and rapid identification of Mycobacterium tuberculosis and its resistance profiles is essential for effective management [7]. While advances in rapid molecular testing and next-generation sequencing have improved diagnostic capabilities, gaps in implementation persist, particularly in high-burden settings [2]. Studies utilizing nucleotide MALDI-TOF MS assays in China have demonstrated promising positivity rates and detailed drug resistance patterns, offering potential improvements in diagnostic efficacy [8]. Furthermore, the evaluation of diagnostic tools such as the EC-test for latent tuberculosis infection in ambulatory people with HIV highlights the ongoing need for robust diagnostic strategies in vulnerable populations [1].The licensed Bacillus Calmette-Guérin (BCG) vaccine, while effective in preventing severe disseminated TB in infants, exhibits variable and limited protective efficacy against adult pulmonary TB, necessitating the development of novel vaccine candidates [9]. Recent research into recombinant BCG vaccines expressing multistage antigens and virus-like particle-based candidates shows promise in providing long-term immunity, yet no new vaccine has been widely licensed for general use [10, 11]. Modeling studies in India suggest that adolescent and adult vaccination with candidates like M72/AS01E could significantly reduce the TB burden, highlighting the potential health and economic impacts of next-generation vaccines [12].The COVID-19 pandemic severely disrupted routine TB control services, leading to a resurgence in global TB incidence and reversing years of progress [2]. Sub-Saharan Africa and Southeast Asia remain the highest-burden regions, where HIV-co-infected individuals face a significantly elevated risk of developing active TB [13]. The intricate relationship between HIV and TB, particularly in settings like South Africa, presents multifaceted challenges influenced by historical, social, and biological factors [13]. In China, the evolving dynamics of TB and emerging HIV co-infection require age-period-cohort analyses to project trends and inform control strategies through 2035 [14].Latent tuberculosis infection (LTBI) represents a vast reservoir for future active cases, with recent analyses challenging the dogma that one-quarter of the world’s population is latently infected, thereby urging a reprioritization of research efforts [15]. Nevertheless, the LTBI population remains substantial, with an annual reactivation rate posing a major threat to long-term control [16]. Cost-effectiveness studies in rural China and Thailand indicate that LTBI testing and preventive treatment among key populations, such as older adults and household contacts, are crucial strategies for achieving the End TB Strategy targets [17, 18]. Specific high-risk groups, including silica-dust-exposed workers and migrants, require targeted screening and management to mitigate LTBI progression [19, 20].Global burden of disease studies from 2010 to 2021 reveal persistent and severe trends in MDR-TB and XDR-TB among adolescents and adults aged 15–49 years, emphasizing the need for focused interventions in this demographic [21, 22]. Mathematical modeling estimates the lifetime disability-adjusted life years (DALYs) for individuals developing rifampicin-resistant TB, highlighting the profound long-term impact of drug resistance on global health [23].

Addressing these challenges requires a comprehensive framework integrating rapid diagnostics, optimized therapeutic regimens, next-generation vaccines, and robust public health strategies to resolve persistent bottlenecks in global TB control. Against this backdrop, anti-tuberculosis research has undergone a paradigm shift from phenotype-driven empirical treatment to mechanism-guided precision intervention. Breakthroughs in structural biology, multi-omics technologies and artificial intelligence have not only refined molecular-level dissection of drug-target interactions and resistance evolution, but also reshaped the therapeutic landscape of treatment regimens, delivery systems and preventive strategies. Most existing reviews, however, remain confined to individual subfields, with limited cross-domain integration of mechanistic insights, clinical evidence and translational bottlenecks, and few explicitly stratify the developmental maturity of emerging technologies. To address this gap, this review provides a critical, multi-dimensional synthesis of anti-tuberculosis research advances spanning 2020 to 2025, with a central focus on bridging basic mechanistic discoveries and clinical translational applications.

**Fig. 1:**
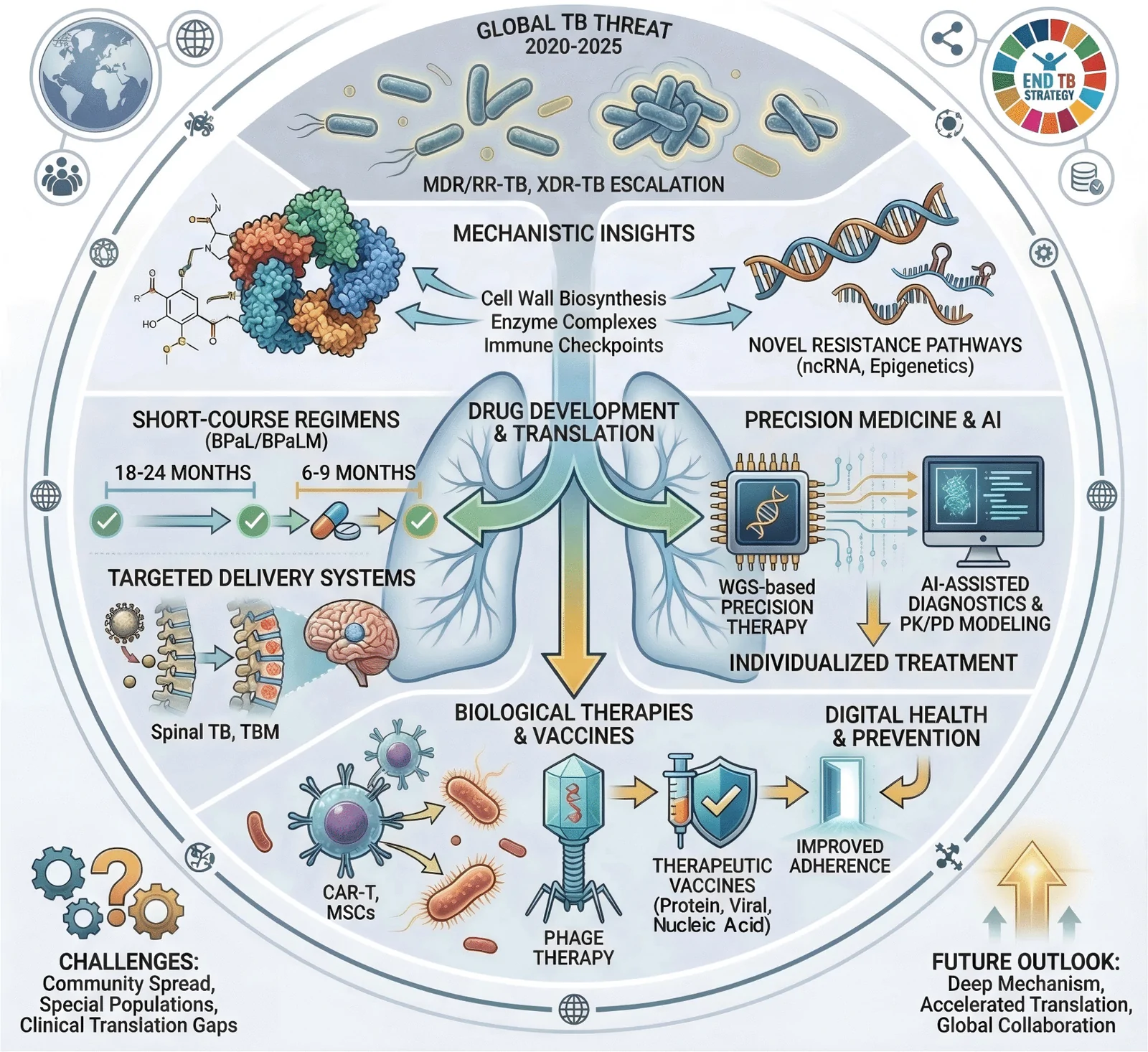
Overview of antituberculosis therapeutic research advances aligned with the WHO End TB Strategy. Landscape of anti-tuberculosis therapeutic research advances from 2020 to 2025, aligned with the WHO End TB Strategy. The spread of drug-resistant tuberculosis (MDR/RR-TB and XDR-TB) drives innovation across six interconnected domains: mechanistic insights into drug targets and resistance pathways; short-course BPaL/BPaLM regimens; precision medicine and artificial intelligence tools; targeted delivery systems for extrapulmonary tuberculosis; host-directed and biological therapies; next-generation vaccines and digital health interventions. Persistent challenges and future priority directions are also outlined

Grounded in structural biology, multi-omics technologies and real-world clinical evidence, the analysis is structured around six interconnected research domains. It opens with mechanistic dissection of drug targets and the spatiotemporal evolution of drug resistance, establishing the foundational framework for therapeutic innovation. Building on these insights, it evaluates progress in novel anti-tuberculosis agents and short-course treatment regimens, followed by precision medicine strategies and targeted delivery systems for extrapulmonary tuberculosis. Shifting focus from direct antibacterial targeting to host-centered interventions, the subsequent section examines host-directed therapies and emerging biotherapies, with explicit stratification of their translational maturity. The review further addresses advances in next-generation vaccines and latent tuberculosis infection intervention strategies. It concludes by delineating priority research directions aligned with the WHO End TB Strategy, offering a translational roadmap to advance global tuberculosis elimination efforts. Full scope from epidemiological burden and mechanistic discoveries to therapeutic advances and future priorities is visually summarized in Fig. 1.

## Antituberculosis drug targets and resistance mechanisms

Deciphering drug-target interaction mechanisms and the spatiotemporal evolution of drug resistance is fundamental to the development of next-generation anti-tuberculosis therapeutics and optimization of existing regimens. The following sections dissect the mechanistic basis of both classic and emerging anti-tuberculosis drug targets, delineate the layered evolutionary trajectory of drug resistance from single-gene mutations to adaptive compensation, and extend to non-genetic resistance pathways and the spatial immune microenvironment of granulomas. Beginning with validated classic targets in energy metabolism and cell wall biosynthesis, recent structural insights into drug action and resistance are elaborated, followed by emerging therapeutic targets including enzyme complexes, protein synthesis machinery and virulence regulators. On this basis, multi-layered resistance mechanisms, cross-resistance networks, and host- and microbiota-associated modulators are analyzed, laying a mechanistic foundation for subsequent discussion of novel drugs and precision treatment strategies.

### Mechanistic deepening and optimization of classic targets

Energy metabolism represents one of the most validated targets for anti-TB drug development. In 2020, bedaquiline's binding site on ATP synthase subunit C was confirmed to locate within the hydrophobic cavity of this subunit, thereby blocking the proton channel and inhibiting enzyme activity [24–26]. In 2023, the conformational blocking mechanism mediated by *Rv0678* mutation was deciphered [27–30]; *Rv0678* encodes a transcriptional repressor that regulates the expression of the MmpS5-MmpL5 efflux pump [27]. When the *Rv0678* gene mutates, it leads to overexpression of the efflux pump MmpS5-MmpL5, which expels bedaquiline from bacterial cells, reducing intracellular drug concentration and thereby inducing drug resistance [27, 28, 31]. In 2025, it was found that bedaquiline disrupts the energy metabolism chain of dormant bacteria, which is crucial for the treatment of latent TB infection [32, 33].Beyond resistance and dormant bacterial activity, structural studies have advanced our understanding of inhibitor selectivity. Our group resolved the inhibitory mechanism of both bedaquiline and its analog TBAJ-587 against mycobacterial ATP synthase, and further characterized their cross-reactivity with human ATP synthase at the molecular level, providing a structural framework for assessing off-target toxicity and optimizing next-generation analogs [34, 35].

Respiratory chain complexes represent another expanding category of energy metabolism targets. Our laboratory determined the co-crystal structure of the mycobacterial cytochrome bcc complex with drug candidates Q203 and TB47, delineating the molecular basis of their inhibition [36, 37]. We further resolved the structure of mycobacterial cytochrome bd oxidase and identified its oxygen channels; these structural findings have been independently validated by multiple international laboratories, confirming conserved inhibitory mechanisms across mycobacterial strains and providing cross-laboratory evidence for structure-based drug optimization. For selectivity evaluation, the structure of human respiratory chain complex II has also been elucidated.Additional energy metabolism enzymes have also been structurally characterized. Our group uncovered the unique architecture of mycobacterial succinate dehydrogenase (SDH) and refined understanding of its trimeric assembly. Furthermore, the cryo-EM structure of DyP-loaded encapsulin in Mycobacterium smegmatis has been resolved, offering insights into bacterial stress response systems [38].

Cell wall synthesis blockade is another core category of classic anti-TB targets. In 2021, the Ddn enzyme activation kinetics of delamanid were elucidated; Strains resistant to delamanid usually exhibit deletion or mutation of dehydrogenase (Ddn) in the F420 biosynthesis pathway, which reduces drug bioactivation [39]. In 2024, pretomanid was found to selectively inhibit mycolic acid synthesis in dormant bacteria, thereby disrupting cell wall integrity [40]; in the same year, our team resolved the three-dimensional structure of Rv3806c, revealing the allosteric regulatory mechanism mediating ethambutol resistance [41].

Intervention in protein synthesis provides another critical therapeutic angle. In 2020, the crystal structure of oxazolidinones binding to the 50S ribosome was discovered [42]; Linezolid, an important oxazolidinone antibiotic, targets mainly the 23S rRNA on the 50S subunit of bacterial ribosomes [43]. Methylation modification of ribosomal RNA is closely related to antibiotic resistance and ribosome function [44–46]; In 2022, it was found that *Mycobacterium tuberculosis* ribosomal RNA methyltransferase TlyA recognizes and modifies the 50S subunit, mediating antibiotic resistance by altering the rRNA modification pattern [46] [67]. tRNA charging (or aminoacylation) efficiency is a key step in protein synthesis, and aminoacyl-tRNA synthetases (aaRSs) are responsible for linking specific amino acids to corresponding tRNAs [47, 48]. Structural studies on *Mycobacterium tuberculosis* phenylalanine-tRNA synthetase (Phe-tRNA synthetase) have revealed the tRNA recognition and aminoacylation processes, which are of great significance for the development of novel antibiotics targeting this site [47, 48].

Drug efflux pump mechanisms represent a major driver of natural and acquired drug tolerance. In 2025, our team identified the heterodimeric ABC transporter MsRv1273c/72c as an isoniazid efflux pump; the asymmetric transport mechanism mediated by its degenerate ATP-binding site is the key to natural tolerance, providing a target for the development of efflux pump inhibitors [38]; We further clarified the structural assembly of the MmpL5/MmpS5 transporter complex, which underlies bedaquiline efflux and resistance [49]. Complementing these findings, the structural resolution of EfpA, the first identified multidrug efflux pump in *Mycobacterium tuberculosis*, provides a blueprint for the design of targeted inhibitors [50]; benzophenanthridine derivatives have been found to have potent activity against multidrug-resistant *Mycobacterium tuberculosis* [51], presumably by inhibiting efflux pumps to enhance the efficacy of existing drugs [51].

Virulence factor regulation and rare adverse reaction mechanisms further expand the scope of target research. The PhoPR system, as an important virulence regulatory factor, has become a potential target for the development of novel TB drugs [52]. A dimer-based screening system has been developed to screen for PhoR dimerization inhibitors, aiming to reduce bacterial virulence [52]. Mtb catalase KatG is crucial for its detoxification mechanism and is also a significant virulence factor [53]. Fluoropyridine and benzothiophene derivatives can kill Mtb and reduce its virulence, suggesting that KatG can be used as a potential target [53]. CRISPR sequencing can identify virulence determinants associated with the persistence of Mtb in macrophages, and two of the most significant genes, *Rv0066c* and *Rv3139*, have been verified and screened [54]. In terms of adverse reactions, some antituberculosis drugs may interact with antiretroviral drugs (ARVs), leading to adverse reactions in HIV/TB co-infected patients [55]; immune reconstitution induced by antiretroviral therapy may cause the immune system to overreact to *Mycobacterium tuberculosis* antigens, thereby triggering paradoxical reactions [56].

Deeper mechanistic elucidation of classical antitubercular drug targets has unveiled the complex molecular basis of drug resistance, encompassing structural alterations of drug targets, impaired prodrug activation, and restricted cell wall permeability. Leveraging structure-based drug design, molecular dynamics simulations, and genomics-guided analyses of compensatory mutations, researchers are optimizing inhibitors against established targets while identifying novel vulnerabilities such as PafA, DprE1, and CanB. Future investigations should prioritize the rational design of multi-target combination therapies to counteract evolving resistance mechanisms, and harness artificial intelligence and machine learning to predict drug permeability and target-binding kinetics, thereby accelerating the development of next-generation antitubercular agents.

Building on recent advances in mechanistic elucidation and optimization of classic antituberculosis drug targets, future research endeavors should be centered on three core directions: structure-guided precision drug design, systematic interception of resistance mechanisms, and rational development of multitarget combination regimens. As *Mycobacterium tuberculosis* resistance mechanisms have expanded from single-target mutations into intricate regulatory networks—exemplified by Rv0678-driven efflux pump overexpression and deficiencies in the F₄₂₀-dependent bioactivation pathway—proactive research strategies must transcend the conventional "discovery-then-validation" model and shift toward a "prediction-and-prevention" paradigm underpinned by high-resolution structural biology and systems pharmacology.

For energy metabolism targets, ATP synthase (the molecular target of bedaquiline) acquires resistance primarily via *Rv0678* mutation-mediated overexpression of the MmpS5-MmpL5 efflux pump, rather than structural modifications to ATP synthase itself. Future investigations should leverage cryo-electron microscopy (cryo-EM) to resolve the dynamic conformational landscape of the intact ATP synthase holoenzyme, and develop dual-site inhibitors that simultaneously engage the c-ring and peripheral stalk to elevate the genetic barrier to resistance. Furthermore, comparative structural modeling should be deployed to assess selectivity against human mitochondrial ATP synthase, thereby refining drug safety profiles. When integrated with lesion-level pharmacokinetic simulations, this strategy will support the design of exposure regimens that minimize Rv0678-triggered efflux induction. For nitroimidazole agents such as delamanid and pretomanid, whose bactericidal activity relies on F₄₂₀-dependent bioactivation, resistance typically arises from loss-of-function mutations in *fbiA*, *fbiB*, *fbiC*, or *ddn*. Going forward, efforts should focus on developing next-generation prodrug analogs independent of a single F₄₂₀-dependent activation step; alternatively, mapping synergistic sensitivity profiles with energy metabolism inhibitors may exploit metabolic vulnerabilities to counteract acquired resistance.

In the realm of protein synthesis targets, phenylalanyl-tRNA synthetase (PheRS) and ribosomal modification enzymes (e.g., TlyA) represent high-value underexploited targets. Future drug design should capitalize on structural divergences in active-site geometry and editing domains between mycobacterial and human tRNA synthetases to develop highly selective allosteric modulators with reduced cross-resistance risk. Saturation mutagenesis analysis and fitness landscape modeling will enable preemptive identification of high-frequency escape variants, while integration of target engagement biomarkers such as thermal proteome profiling into early clinical translation will allow real-time monitoring of in vivo drug efficacy. In parallel, research on ribosomal modification mechanisms should delineate their synergistic interactions with existing ribosome-targeting agents to forestall the emergence of multidrug-resistant strains.

The regulatory network of drug efflux pumps constitutes a core driver of resistance evolution. Beyond the well-characterized Rv0678 axis, the identification of novel efflux systems such as MsRv1273c/72c has uncovered an unrecognized pathway of isoniazid tolerance. Future studies should employ multi-omics and chemical genetics approaches to systematically map master regulators of the efflux network and characterize their transcriptional dynamics under physiologically relevant pharmacokinetic exposure. Priority should be given to developing efflux pump inhibitors with broad substrate coverage and acceptable cardiac safety margins, followed by optimization of combination dosing schedules with bedaquiline or clofazimine to mitigate efflux induction and hysteresis effects. Additionally, single-cell imaging and quantitative lesion pharmacokinetics will be applied to characterize drug permeability and intracellular distribution, narrowing the translational gap between in vitro assays and in vivo efficacy.

Regarding virulence factor regulation and the host–pathogen interface, future therapeutic strategies should integrate host-directed adjuvant therapy with classic antibacterial targeting. Attenuation of stress response pathways—including DosR regulon modulation and lipid metabolic rewiring—will diminish stress-induced drug tolerance. Remodeling the redox and lipid microenvironment of macrophages will erode the protective niche for persistent bacterial subpopulations. Pairing immunomodulatory agents with bactericidal drugs will enable synchronous bacterial killing and lesion restructuring, thereby accelerating overall sterilization kinetics.

Finally, the rational design of combination regimens remains the cornerstone of resistance mitigation. Quantitative systems pharmacology and chemogenetic interaction mapping will guide the construction of regimens that exert orthogonal selective pressure on discrete resistance axes: efflux machinery, bioactivation cascades, and target-site mutations. Dosing sequences and treatment schedules should be optimized to exploit temporal windows of vulnerability—for instance, triggering energy collapse prior to cell wall biosynthesis inhibition. Integration of rapid genotypic resistance surveillance and adaptive treatment adjustments will further sustain long-term efficacy. Resistance suppression performance will be validated via stochastic modeling and clinical microbiology datasets to ensure robust activity across heterogeneous lesion compartments. Taken together, the next generation of antituberculosis drug development will deeply converge structural biology, systems pharmacology, and host immunology to build a precise, high-efficacy therapeutic framework with durable resilience against drug resistance.

### Emerging targets and breakthroughs in drug development

In TB drug target research, Surface Plasmon Resonance (SPR) technology, featuring label-free, real-time, and high-sensitivity characteristics, has emerged as a core tool for deciphering drug-target interaction kinetics and affinity. By monitoring minute changes in refractive index at the metal–dielectric interface in real time, SPR directly quantifies the association rate constant (kon), dissociation rate constant (koff), and equilibrium dissociation constant (KD) of biomolecular interactions, providing critical quantitative data for the screening and optimization of lead drug compounds [57, 58].

In practical applications, SPR technology has been widely employed for the validation of diverse complex targets, including G protein-coupled receptors (GPCRs), membrane proteins, and RNA-small molecule interactions [59–61]. For GPCRs—targets that have traditionally been challenging to purify and immobilize—SPR has enabled direct measurement of ligand binding kinetics through immobilization strategies utilizing lipid nanodiscs or detergent micelles, filling the gap left by indirect signaling assays that cannot provide direct affinity data [59]. Furthermore, in antibody drug development, SPR serves not only as a key screening tool for affinity maturation but also enables rapid epitope binning of large-scale antibody libraries through high-throughput SPR imaging (SPRi) technology [58, 62]. For example, using the BreviA high-throughput SPR analysis system, researchers can rapidly process vast amounts of antigen–antibody interaction data during data-driven antibody design, significantly accelerating the selection of candidate molecules [62].

Nevertheless, the success of SPR experiments is highly dependent on rigorous experimental design and data quality control [63, 64]. Common technical pitfalls include nonspecific binding, ligand inactivation, and mass transport limitation effects arising from diffusion-controlled processes [57, 65]. To overcome mass transport limitations and ensure that intrinsic kinetic parameters rather than apparent parameters are measured, experimentalists must optimize flow rates and reduce ligand immobilization density to minimize the Damköhler number [66]. Concurrently, the implementation of a dual reference subtraction strategy—using both a blank channel and buffer injection as references—is essential for eliminating bulk effects and instrument drift [63, 64]. In recent years, the integration of computational approaches, including the development of high-throughput data analysis tools such as TitrationAnalysis, has further improved the efficiency and accuracy of SPR and biolayer interferometry (BLI) data processing, enabling integrated analysis of kinetic data across multiple platforms [67].

SPR occupies a dominant position in the validation of drug-target interactions by virtue of its label-free, real-time, and high-sensitivity characteristics [58, 68]. Although potential artifacts arising from surface immobilization remain a concern, rational experimental design combined with orthogonal validation using solution-phase techniques such as ITC and MST enables SPR to provide highly valuable kinetic and affinity data [69, 70]. Looking forward, advances in microfluidic technology and integration with other omics approaches, such as chemoproteomics, will position SPR to play an even more profound role in unraveling complex biological mechanisms and accelerating innovative drug development [71, 72].

Cell wall biosynthesis remains a rich source of emerging drug targets. In 2023, the structure of arabinosyltransferase AftA was reported, revealing its role in arabinan assembly [73]; In 2024, the cryo-EM structure of phosphoribosyltransferase Rv3806c in the presence of donors was reported [41]; The mechanism of DprE2 is less clear than that of DprE1, but it has been considered a potential target for the treatment of drug-resistant *Mycobacterium tuberculosis*, and comprehensive computational methods aim to identify DprE2 inhibitors [74]. MabA is a key enzyme in the fatty acid synthase II (FAS-II) system, participating in the elongation cycle of mycolic acids [75], and some compounds with potential MabA inhibitory activity have been identified [76]; KasA, another key component of the FAS-II system, the potential of marine mollusk metabolites as KasA inhibitors to interfere with mycolic acid biosynthesis has been reported [77]; Polyketide synthase 13 (Pks13) is responsible for combining long-chain fatty acids produced by fatty acid synthase I and FAS-II systems to form mycolic acids [78, 79]; CMX410 can irreversibly inhibit the acyltransferase domain of Pks13 [78]; Targeting Pks13, a key enzyme in mycolic acid biosynthesis, has emerged as a promising approach against drug-resistant TB. Through structure-based virtual screening and rational design, our laboratory developed pyrimido[1,2-a]imidazole derivatives as potent Pks13-TE inhibitors, offering new candidate molecules for addressing drug-resistant tuberculosis [80]. Building on this, we further identified novel furo[2,3-b]isoquinoline derivatives targeting Pks13 that retain strong antimycobacterial activity while significantly reducing hERG-related cardiotoxicity risk, representing a notable advance in safety optimization [81].Mycolic acid transporter MmpL3 is responsible for transporting mycolic acids to the outer layer of the cell wall [82, 83]; Trisubstituted benzimidazole derivatives can significantly reduce the number of bacteria in mouse models [82], and thienopyrimidine amide analogs also exert antituberculosis activity by targeting MmpL3 [83]; L,D-transpeptidases, distinct from D,D-transpeptidases mainly targeted by β-lactam antibiotics, may be more important in drug-resistant strains, and fragment-like compounds targeting L,D-transpeptidases have been verified [84]; Cell wall core assembly protein CpsA plays a role in the core assembly of the *Mycobacterium tuberculosis* cell wall [85]; Compound ACA (3-azidothiophene-2-carboxylic acid) has been found to kill *Mycobacterium tuberculosis* and Mycobacterium bovis by targeting CpsA [85].

Enzyme complex regulation and protein degradation innovation represent novel directions for target expansion. Polyamines play important roles in bacterial metabolism, and drug development targeting key enzyme complexes in the Mtb polyamine metabolic pathway is underway [86]; Eis protein is an acetyltransferase that acetylates aminoglycoside antibiotics, preventing their binding to bacterial ribosomes, thereby leading to Mtb resistance to aminoglycoside antibiotics [87]. Computational drug discovery methods have been used to target the Eis protein to inhibit Mtb virulence and drug resistance, promising the development of novel antituberculosis drugs [87]; Rv1151c is a bifunctional enzyme in Mtb with deacetylation and desuccinylation activities, playing important roles in Mtb drug resistance and stress response. Through drug repurposing, the antidiabetic drug ipragliflozin has been found to inhibit the Rv1151c protein and act synergistically with isoniazid [88]. Targeted Protein Degradation (TPD) technology has antibacterial effects and can enhance the efficacy of antibiotics [89]; TPD can target various "undruggable" pathogenic proteins in Mtb, such as some proteins involved in resistance mechanisms or key transcription factors, promising the development of novel antituberculosis drugs that overcome drug resistance and shorten treatment duration [89–91].

Dormant bacteria targeting, immune checkpoint targets and microbiota-host interaction targets further broaden the therapeutic landscape. Mtb MS is a key rate-limiting enzyme in the glyoxylate cycle, crucial for the survival of dormant Mtb but absent in the host [92], and novel natural inhibitors of Mtb MS have been identified [92]; Mtb enters a dormant state under carbon starvation conditions and adapts to the environment by altering proteomics [93]. Characterization of carbon starvation-induced proteins helps identify potential drug targets related to dormancy [93] [96]; RpfB protein is crucial for awakening dormant cells [94], and targeting RpfB may help eliminate dormant Mtb and prevent its reactivation [95]. In addition to directly targeting bacteria, regulating host immune responses is also a strategy to eliminate dormant bacteria. For example, human β-defensin-2 may affect bacterial survival by inducing Mtb to enter a dormant state [95]. Granulysin has been found to promote Mtb dormancy, indicating its complex role in the occurrence and development of TB and potential therapeutic intervention points [96]. In TB, Mtb evades host immunity through multiple mechanisms, including inhibiting phagosome-lysosome fusion, resisting reactive oxygen species and reactive nitrogen species, and interfering with host signaling pathways [97]. For example, the PknF protein secreted by Mtb can inhibit NLRP3 inflammasome activation, and lipid-mediated defense mechanisms also counteract host immune responses [97]; PD-1/PD-L1 has important effects on host immune responses during Mtb infection. Overexpression of lymphocyte activation gene 3 (LAG-3) may be a potential indicator of immune dysfunction in TB patients [98]. Its upregulation may lead to T cell exhaustion, thereby impairing the body's ability to clear Mtb [98]; By regulating immune checkpoints, the killing ability of host cells such as macrophages against Mtb can be enhanced, thereby improving the therapeutic effect of TB [99]. For example, the hypoxia-inducible factor-1α (HIF-1α) signaling pathway plays an important role in Mtb infection, and regulating this pathway may become a new target for antituberculosis therapy [100]. Host-Directed Therapy (HDT) aim to regulate host immune responses and enhance their ability to clear Mtb, thereby serving as an auxiliary strategy for traditional antituberculosis treatment [101, 102]. The mechanisms of HDT include inducing autophagy, regulating inflammatory responses, and improving immune cell function [102]. For example, small molecules, proteins, lipids, vitamins, and polysaccharide-based therapeutics are used to regulate host responses [102]; Protease inhibitors, as HDT, have shown potential in controlling Mtb and HIV co-infection [103]; Mtb can manipulate host iron homeostasis to promote its growth, such as regulating host iron metabolism by hijacking the UBE2O pathway, which is a potential drug target [104].

Surface plasmon resonance technology exerts irreplaceable value in drug-target interaction validation. It enables real-time, label-free characterization of binding kinetic parameters between small-molecule inhibitors and target proteins, delivering accurate thermodynamic and kinetic data to support high-throughput screening. However, SPR technology remains confronted with challenges in handling low-affinity interactions and interference from complex biological matrices, necessitating the implementation of stringent quality control protocols to ensure data reliability.

As a mycolic acid transporter, MmpL3 has emerged as a highly promising novel target given that its inhibition results in compromised cell wall integrity, even though the structural complexity of its transmembrane domains places higher demands on drug permeability. The identification of novel targets such as Rv1151c, coupled with targeted protein degradation technology that degrades pathogenic proteins through bacteria-specific ubiquitin-like systems or proteasomal pathways, offers a brand-new paradigm for clearing persister bacteria. Distinct from the conventional "occupancy-driven" pharmacological mode, this "event-driven" pattern can effectively counteract drug resistance caused by target mutations, and demonstrates unique advantages especially against low-metabolic bacterial populations embedded in biofilms.

**Fig. 2:**
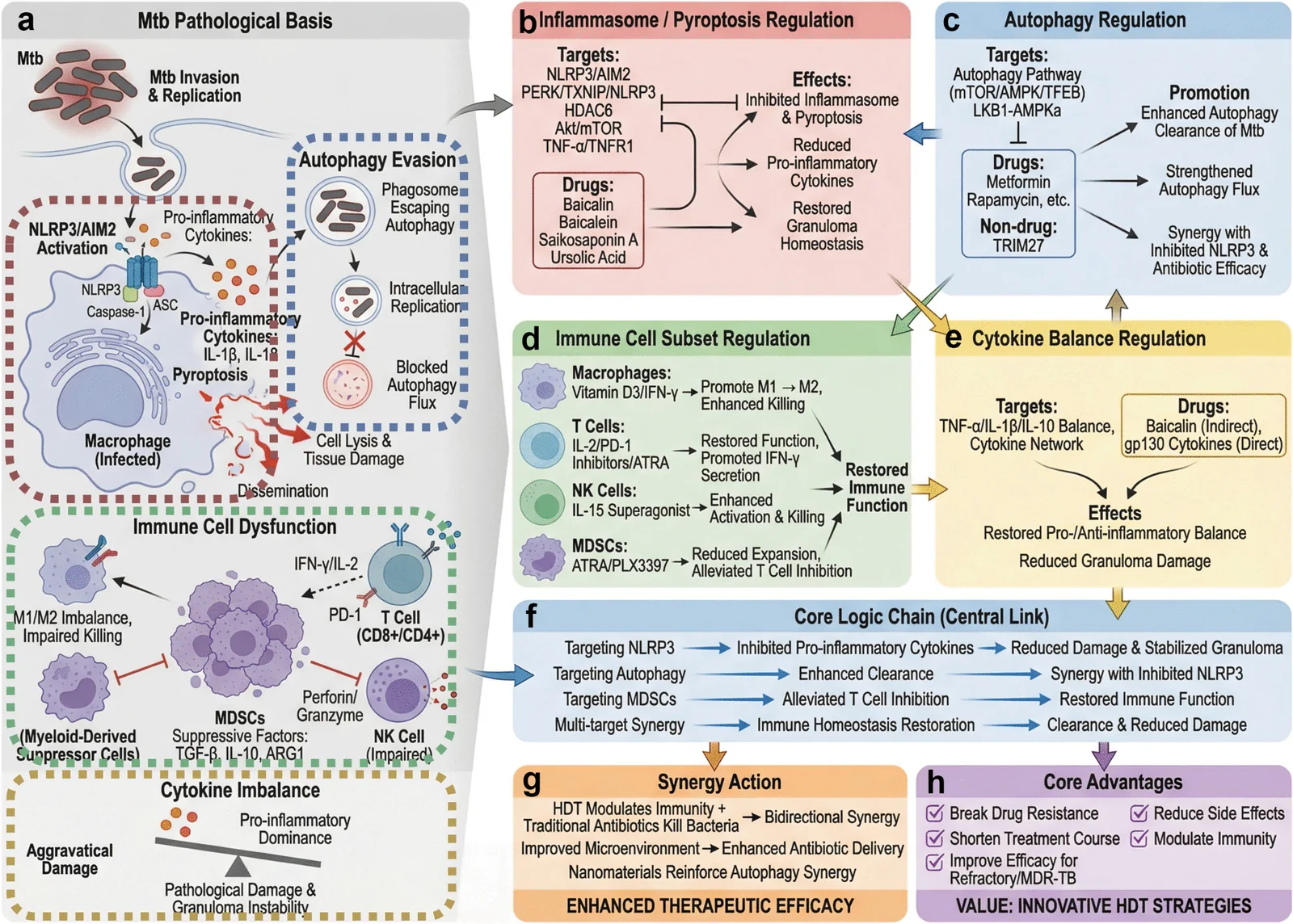
Integrated immune regulatory network of host-directed therapy for tuberculosis: targets, agents, effects and translational advantages. Integrated immune regulatory network of tuberculosis host-directed therapy (HDT), mapping targets, agents, functional outputs and translational advantages across four axes. **a** Mtb infection pathology. Mtb invasion triggers macrophage inflammasome activation, autophagy blockade, immune dysfunction and cytokine imbalance, driving tissue damage and bacterial dissemination. **b** Inflammasome/pyroptosis regulation. Baicalin, baicalein and related agents target PERK/TXNIP/NLRP3 and Akt/mTOR axes to suppress excessive pyroptosis and restore granuloma homeostasis. **c** Autophagy regulation. Metformin, rapamycin and TRIM27 modulate mTOR/AMPK/TFEB pathways to enhance autophagic Mtb clearance and synergize with antibiotics. **d** Immune cell modulation. Vitamin D₃, IFN-γ, IL-2/PD-1 inhibitors and ATRA reprogram macrophage polarization, restore T/NK cell function and alleviate MDSC-driven immunosuppression. **e** Cytokine balance regulation. Baicalin and gp130 rebalance the TNF-α/IL-1β/IL-10 network and reduce granulomatous damage. **f** Core logic chain. Targeting inflammasome/autophagy pathways stabilizes granulomas; targeting MDSCs restores T cell function; multi-target synergy restores immune homeostasis and promotes bacterial clearance. **g** Synergy with antibiotics. HDT modulates host immunity while antibiotics directly kill Mtb, generating bidirectional synergy and enhancing lesional drug penetration. **h** Translational advantages. HDT overcomes drug resistance, reduces adverse events, shortens courses and modulates immunity, with high value for refractory MDR-TB

Looking forward, research efforts should be directed toward the rational design of multi-target combination therapies and personalized precision medicine. First, artificial intelligence and machine learning algorithms should be deployed to predict drug permeability across the intricate bacterial cell wall and the synergistic effects of multi-target interventions, thereby optimizing the pharmacokinetic profiles of lead compounds. Second, real-time monitoring platforms based on highly sensitive techniques represented by SPR should be developed to dynamically assess the intracellular target occupancy of drugs and the evolutionary trajectories of resistance mutations within host cells. The integrated immune regulatory network of host-directed therapy for tuberculosis, mapping molecular targets, representative pharmacological agents, functional effects and translational advantages across four mechanistic axes, is systematically depicted in Fig. 2.

### Spatiotemporal evolution of drug resistance mechanisms

Classic resistance escalation remains the primary driver of the global drug-resistant TB epidemic. Globally, especially in regions such as West Africa, the continuous evolution of drug-resistant Mtb strains has become a major public health problem, posing a serious threat to the achievement of the "End TB Strategy" [105]. In many high-burden regions of drug-resistant TB, the epidemic of drug-resistant TB is no longer solely driven by acquired resistance in individuals with inadequate treatment or poor adherence; community transmission has become common [106]. For example, in Vietnam, Beijing family Mtb strains have driven the evolution and transmission of antibiotic resistance [107]. Through genomic evolution and dynamic analysis of Mtb strains, the transmission routes and geographical distribution of drug resistance can be better understood [105, 108].

Novel resistance pathways beyond classic coding region mutations are increasingly recognized. In addition to mutations in gene-coding regions, non-coding RNAs play roles in regulating drug tolerance of Mtb [109]. In addition, epigenetic modifications have been found to affect Mtb gene expression, thereby influencing its drug resistance. These mechanisms do not change the DNA sequence but affect gene expression by altering gene packaging or marking, for example, leading to upregulation or downregulation of drug resistance-related genes [110–112]; Mtb can adapt to drug pressure and develop drug resistance by altering its metabolic pathways [109, 113]. For example, CRISPR screening and comparative liquid chromatography-mass spectrometry techniques have been used to identify genes affecting the efficacy of Delamanid and Pretomanid and characterize the metabolic pathways of these drugs in Mtb, providing insights for the development of novel antituberculosis drugs and targeted treatment regimens for drug-resistant TB [114]. Another omics analysis study identified the *Rv3094c* gene, which is related to the metabolism of ethionamide, revealing a new mechanism of drug resistance, especially in 5%−30% of Mtb strains, where resistance is not associated with reported single nucleotide polymorphisms (SNPs) and may involve changes in gene/protein expression [115]; the unique cell wall structure of Mtb endows it with natural resistance to various antibiotics [116–118]. Drugs targeting the cell wall synthesis pathway, such as ethambutol and isoniazid, act by inhibiting mycolic acid synthesis or peptidoglycan layer formation [111]. Mtb can counteract these drugs by changing the composition of the cell wall or increasing its impermeability, thereby limiting drug entry into the cell [111, 118].

Mtb can prevent phagosome-lysosome fusion to avoid degradation; it can regulate the production of reactive oxygen species and reactive nitrogen species in macrophages to reduce the host's antibacterial ability; it can regulate cell death pathways such as apoptosis, necroptosis, and autophagy to facilitate its own survival and transmission [119, 120]; Mtb infection leads to the formation of granulomas, which are both the host's attempt to limit infection and provide a hidden and persistent microenvironment for bacteria. Within granulomas, Mtb can enter a low metabolic state to evade immune clearance and drug action [120, 121]; it can manipulate the autophagic process of host cells to escape immune responses and promote its own intracellular replication; it can regulate host cytokine pathways by secreting various molecules, such as inhibiting pro-inflammatory cytokines and enhancing anti-inflammatory cytokines, thereby impairing the host's immune response [119, 120]; drug-resistant Mtb strains may have complex interactions with the host immune system. For example, rifampicin resistance can affect host immune responses, leading to immune dysregulation and thereby exacerbating disease progression [122]. This immune dysregulation may manifest as changes in the cytokine profile, thereby affecting the recruitment and function of immune cells [122].

Cross-resistance networks and drivers of resistance evolution further shape the evolutionary trajectory of drug-resistant strains. With the introduction of new generation antituberculosis drugs such as bedaquiline and delamanid, the treatment landscape of drug-resistant TB has changed [123, 124]. However, resistance to these new drugs has also emerged and may cross-react with other drugs [125]. For example, fluoroquinolones are key second-line drugs for the treatment of MDR-TB; if MDR/RR-TB patients are also resistant to fluoroquinolones, they are classified as pre-XDR-TB [112]; recent studies have revealed potential "trade-off drug phenotypes" associated with the evolution of antibiotic resistance [126]. This means that while bacteria develop resistance to a certain drug, they may develop higher sensitivity or stronger resistance to another drug. For example, the evolution of resistance to certain antibiotics may lead to reduced sensitivity to other antibiotics, making specific drug combinations ineffective. Understanding these complex relationships helps design more optimized combination therapy regimens to overcome or delay the development of resistance [126]. Whole-genome sequencing data analysis of TB patients in eastern China from 2015 to 2021 showed that the acquisition of drug-resistant mutations follows a specific order. For example, the *katG* S315T mutation occurs first, followed by other resistance-related mutations [127]. This dynamic process of mutation acquisition provides valuable clues for understanding how Mtb develops drug resistance; stressors in the host microenvironment such as redox status, acidity, hypoxia, and nutrients can induce Mtb to produce drug-tolerant phenotypes [110]. In addition, uneven permeability and distribution of drugs in infection lesions can also promote the development of drug resistance [112].

Microbiota-associated resistance represents an underrecognized dimension of drug resistance evolution. The lungs were once considered a sterile environment but have now been found to have their own unique microbiota [128]. These microorganisms play key roles in host immune system development and resistance to pathogen infection. Mtb infection itself and antibiotic treatment can alter the composition of the microbiota, thereby affecting the host's resistance to infection and drug efficacy [128]; The gut microbiota produces a variety of metabolites that can directly or indirectly affect the host's immune response and even the growth and drug sensitivity of Mtb. Dysbiosis may alter the balance of these metabolites, thereby favoring the growth of drug-resistant strains; It may impair host immune function, making it more difficult to clear Mtb infection, and may even promote the emergence and transmission of drug-resistant strains [128]; It may change the pH value, oxygen level, and availability of nutrients in the local host microenvironment, which can affect the physiological state and drug sensitivity of Mtb [110]. Although Mtb is usually genomically conserved, other bacteria or phages may transfer resistance genes to Mtb or to microorganisms sharing the same niche as Mtb through horizontal gene transfer, thereby indirectly affecting the drug resistance of Mtb. The high diversity and close interaction of microorganisms in the gut provide a potential environment for such gene transfer [129]; Phages can indirectly affect the drug sensitivity of Mtb by inducing bacterial stress responses or altering their physiological state. Although there is currently no direct evidence that phages directly mediate the transfer of resistance genes in Mtb, their impact on the structure and function of the host bacterial community cannot be ignored [129].

Recent breakthroughs in high-resolution spatial biology technologies, including spatial transcriptomics, multiplex immunofluorescence (mIF), and imaging mass cytometry, have enabled researchers to reconstruct three-dimensional spatial immune atlases of granulomas at single-cell resolution, unveiling unprecedented cellular heterogeneity and functional compartmentalization within these structures.

To validate and refine transcriptomic findings at the protein level, multiplex immunofluorescence technology has undergone a transformative evolution from conventional cyclic staining to spectral imaging and Opal tyramide signal amplification (Opal-TSA) methodologies. Opal-TSA technology permits multiplex protein labeling in thick formalin-fixed paraffin-embedded (FFPE) tissue sections, enabling simultaneous detection of epithelial compartments, immune cell populations, and bacteria within pulmonary tuberculosis lesions [130]. This approach overcomes the limitation of traditional immunohistochemistry, which can only detect single markers, thereby allowing researchers to dissect complex cellular proximity relationships within the same field of view [131]. For instance, single-step multiplex immunofluorescence based on spectral imaging (SISS-mIF) technology achieves multicolor labeling in a single staining step through automatic optimization of tissue autofluorescence spectra, substantially enhancing diagnostic efficiency and the accuracy of spatial clustering analysis [132]. In terms of technological integration, the combination of spatial transcriptomics with multiplex immunofluorescence has emerged as a pivotal strategy for deciphering hierarchical transcriptome-phenotype relationships. The ST-FFPE-mIF protocol performs nine-plex immunofluorescence staining on the same FFPE tissue section following spatial transcriptomic data acquisition via Stereo-seq, achieving precise correspondence between gene expression and protein localization [133]. This multimodal integration not only confirms the cell types corresponding to specific gene clusters but also reveals alterations in the spatial distribution of regulatory T cells following specific therapeutic interventions [134]. Furthermore, deep learning algorithms have been applied to analyze multiplex immunofluorescence images in diversity outbred mouse models, enabling discrimination between asymptomatic latent infection and severe fatal tuberculosis disease states through the identification of cell-based spatial features, demonstrating the substantial potential of artificial intelligence in interpreting complex spatial immune data [135].

Despite remarkable progress in spatial immune atlas construction, numerous challenges persist. First, the profound heterogeneity of granulomas across individuals necessitates multicenter studies with larger sample sizes to establish universally applicable classification criteria [136]. Second, current spatial technologies are predominantly performed on two-dimensional sections; although three-dimensional imaging approaches such as light-sheet fluorescence microscopy combined with tissue clearing techniques are under development, their application in large-scale clinical samples remains limited [137]. Finally, translating static spatial snapshots into dynamic models of disease progression requires the integration of computational modeling and time-series data [138, 139]. Future research should focus on integrating spatial metabolomics, transcriptomics, and proteomics to construct comprehensive multidimensional spatial atlases, thereby precisely identifying key cellular interaction nodes that drive disease progression and providing a robust theoretical foundation for the development of novel host-directed therapies that break immune tolerance and promote bacterial clearance [140, 141].

Building on current comprehensive insights into canonical resistance escalation, novel non-coding regulatory pathways, host microenvironment-mediated tolerance, and intricate cross-resistance networks, future research priorities should center on three core fronts: constructing high-resolution spatiotemporal dynamic models, developing precise intervention strategies against non-conventional targets, and establishing a systematic therapeutic framework for host–pathogen–microbiome interactions.

For the construction of high-resolution spatiotemporal evolutionary landscapes and predictive models, the community transmission of classically resistant strains—particularly the global spread driven by Beijing-lineage *Mycobacterium tuberculosis*—has revealed pronounced lineage specificity and geographic clustering in antimicrobial resistance evolution. Moving beyond conventional static genome sequencing, future investigations must shift toward integrated analyses of spatiotemporal transcriptomics and spatial metabolomics at single-cell resolution. By building digital twin models that simulate dynamic shifts in the metabolic states and resistance phenotypes of Mtb subpopulations across distinct granuloma compartments (e.g., the hypoxic core and immunologically active periphery), researchers can accurately predict the sequential acquisition of resistance mutations and their clonal evolutionary trajectories within the host. This multiscale modeling approach enables the identification of heteroresistance as an early warning precursor to full-blown drug resistance, making it possible to interrupt resistance evolution prior to the emergence of clinically detectable phenotypes.

Regarding the development of novel therapeutics targeting non-coding regions and epigenetic regulation, small-molecule inhibitors of Mtb epigenetic regulators—such as DNA methyltransferases and histone-like protein-modifying enzymes—hold the potential to reverse bacterial drug tolerance and restore susceptibility to existing antibiotics. For instance, modulating miRNA expression in host cells not only shapes host immune responses but may also indirectly tune bacterial stress adaptation pathways. Furthermore, in-depth dissection of Mtb metabolic plasticity under host-imposed stress, especially the mechanistic link between lipid metabolic reprogramming and persister formation, will uncover novel targets for the development of metabolically lethal antimicrobial agents.

In the area of host microenvironment modulation and immuno-metabolic combination therapy, the complex granuloma microenvironment—defined by hypoxia, acidic pH, and nutrient limitation—represents a primary driver of drug-tolerant states in Mtb. Future therapeutic strategies will transition from purely bactericidal approaches toward microenvironment remodeling. Host-directed therapies can be deployed to modulate immune cell function within granulomas, enhance drug penetration, and eliminate the niches that sustain persister survival. Promising translational strategies include targeting cell death pathways to boost the bactericidal capacity of macrophages, and leveraging nanoparticle delivery systems to overcome cell wall permeability barriers and deliver drugs precisely to the granuloma core.

**Fig. 3:**
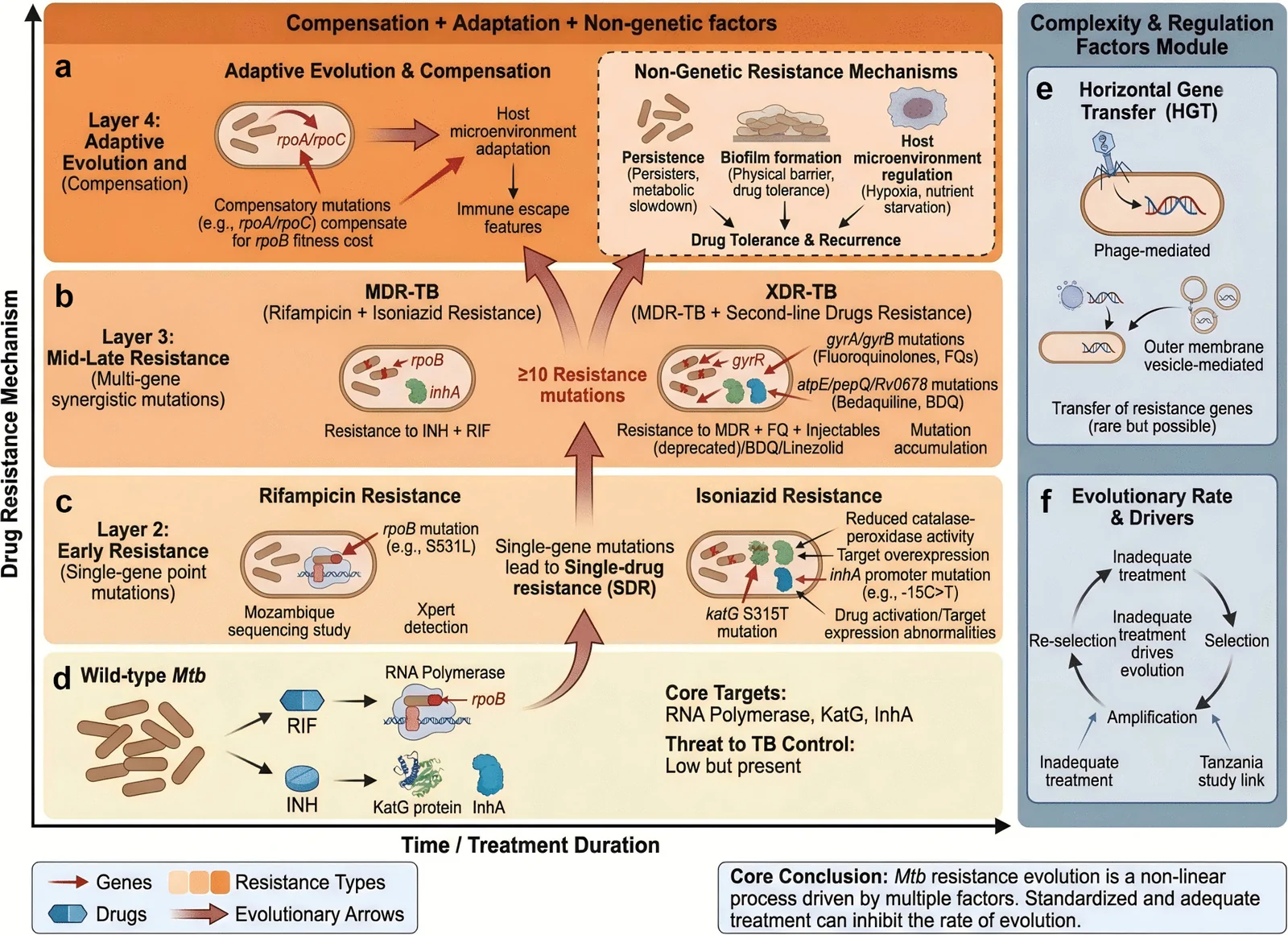
Stepwise evolutionary trajectory of drug resistance in *Mycobacterium tuberculosis* across treatment duration. Stepwise evolutionary trajectory of Mtb drug resistance over the treatment course. **a** Early resistance. Single-gene point mutations drive single-drug resistance: *rpoB* for rifampicin; *katG/inhA* for isoniazid. Detectable via Xpert and whole-genome sequencing. **b** Mid-late resistance. Accumulation of ≥ 10 synergistic mutations produces MDR-TB and XDR-TB, with additional resistance to fluoroquinolones and second-line injectables. **c** Adaptive evolution & non-genetic resistance. Compensatory *rpoA/rpoC* mutations reduce fitness costs; persister dormancy, biofilms and microenvironment plasticity drive treatment failure. **d** Regulatory modulators. Horizontal gene transfer is a potential source of novel resistance; inadequate treatment accelerates evolution, while standardized therapy decelerates it. **e** Complexity module. Horizontal gene transfer expands resistance evolution beyond chromosomal mutations, representing an underrecognized acquisition route. **f** Evolution drivers. Inadequate treatment and poor adherence impose strong selection pressure; standardized consistent therapy slows resistance evolution. INH, isoniazid; RIF, rifampicin; FQs, fluoroquinolones; BDQ, bedaquiline; SDR, single-drug resistance; Xpert, GeneXpert MTB/RIF assay

For deciphering cross-resistance networks and optimizing combination regimens, resistance mutations do not exist in isolation but form elaborate cross-resistance networks, wherein certain resistance mutations may confer heightened susceptibility to other agents (i.e., trade-off phenotypes). Future studies need to systematically map the genome-wide cross-resistance landscape of Mtb and identify drug combinations that exert "negative synergistic effects". Guided by quantitative pharmacology models, combination regimens incorporating novel agents such as bedaquiline and delamanid should be optimized by integrating not only pharmacodynamic properties but also inter-individual host variability and microbiome metabolic factors. Through real-time monitoring of microbiome dynamics and the abundance of resistant subpopulations during treatment, the implementation of adaptive dosing strategies is expected to fully halt further resistance evolution and achieve precision cure for tuberculosis. The stepwise evolutionary trajectory of Mtb drug resistance across treatment duration, encompassing genetic mutation accumulation, adaptive compensation, non-genetic mechanisms and evolutionary modulators, is schematically illustrated in Fig. 3.

### Stratified treatment regimens for drug-resistant TB

The clinical application and promotion of the BPaL regimen is one of the most significant advances in the treatment of susceptible MDR-TB. This regimen shortens the treatment duration from the traditional 18–24 months to 6–9 months and significantly improves the treatment success rate [142–144]. The BPaL regimen has achieved ~90% cure rates in MDR-TB and XDR-TB, primarily based on pivotal trials like Nix-TB, revolutionizing treatment with a short-course, all-oral regimen. Real-world data from TB-PRACTECAL and multicountry cohorts confirm its efficacy, though optimized linezolid dosing is crucial to balance toxicity and effectiveness. While challenges remain, including limited long-term follow-up, drug accessibility, and implementation in resource-limited settings, BPaL represents a transformative breakthrough in drug-resistant TB treatment.

For pre-XDR/XDR-TB, a multicenter cohort study evaluated the safety and efficacy of three 9-month all-oral five-drug regimens in MDR-TB and pre-XDR-TB patients [145]. These regimens include bedaquiline, linezolid, moxifloxacin, cycloserine, and pyrazinamide (Regimen A), and linezolid, moxifloxacin, cycloserine, clofazimine, and pyrazinamide (Regimen B). The results showed that these short-course regimens help improve patient treatment adherence and increase treatment success rates [145]. For refractory cases, new treatment regimens, such as those containing benzimidazole drugs, are being developed to improve treatment outcomes, especially for patients with drug-resistant TB [146, 147].

Future research and clinical practice priorities should focus on large-scale validation of real-world evidence, precise management of drug toxicity, integrated development of agents targeting novel mechanisms, and widespread adoption of adaptive clinical trial designs, with the overarching goal of establishing a more stratified, personalized, and globally applicable diagnostic and therapeutic framework for drug-resistant tuberculosis.

#### Large-scale validation of real-world evidence and optimized programmatic implementation

Although pivotal clinical trials such as Nix-TB have laid the efficacy foundation for the BPaL regimen, the core challenge in translating this regimen into a global public health strategy lies in its feasibility and safety monitoring in real-world settings. Future directions emphasize the establishment of large-scale, multinational, multicenter registry cohort studies to evaluate long-term recurrence rates, the risk of acquired drug resistance, and mortality associated with BPaL/BPaLM regimens across diverse epidemiological contexts, including high-burden regions such as South Africa, India, and Southeast Asia. Specifically, among patients with pre-extensively drug-resistant TB (pre-XDR-TB), the superiority of the moxifloxacin-containing BPaLM regimen over the fluoroquinolone-free BPaL regimen in preventing resistance evolution warrants further validation. Furthermore, early implementation experiences from settings including Pakistan and Germany indicate that enhanced monitoring and management of linezolid-related toxicities, such as peripheral neuropathy and myelosuppression, are critical to ensuring treatment completion rates. Going forward, standardized guidelines for toxicity grading and management should be developed, and optimal strategies to balance efficacy and safety via linezolid dose adjustments—for instance, reducing the dose from 1200 to 600 mg or implementing individualized titration based on therapeutic drug monitoring—need to be explored.

#### Clinical translation of benzimidazole agents

Incorporation of DprE1 inhibitors (such as benzothiazinone analogs or novel benzimidazole derivatives) into BPaL-based intensified regimens may provide a salvage therapeutic option for highly refractory drug-resistant TB. Additionally, investigations into DprE2, the isozyme of DprE1, have revealed that active metabolites of pretomanid and delamanid may target DprE2, suggesting that combining nitroimidazole agents with distinct mechanisms or developing dual-targeting inhibitors may yield synergistic effects.

#### Integration of adaptive clinical trial design and precision medicine

Conventional randomized controlled trials (RCTs) appear rigid and time-consuming in addressing the rapidly evolving landscape of drug-resistant *Mycobacterium tuberculosis* strains. The future research paradigm for drug-resistant TB will shift toward adaptive platform trials, as exemplified by the TB-PRACTECAL trial, which allows simultaneous evaluation of multiple interventions (e.g., BPaL, BPaLC, BPaLM) within a single framework and dynamic adjustment of trial arms based on interim analysis results. This "learning health system" approach can accelerate the identification of effective regimens and the discontinuation of ineffective combinations, thereby substantially improving research and development efficiency.

Aligned with the personalized medicine research methodology advocated by the PERMIT guidelines, future investigations will deeply integrate genomic, transcriptomic, and metabolomic data to identify biomarkers predictive of treatment response and toxicity risk. For instance, rapid genotypic testing to identify key resistance mutations in genes such as *rpoB*, *katG*, and *gyrA* can be applied not only for diagnostic purposes but also to guide stratified treatment: BPaLM is prioritized for fluoroquinolone-susceptible patients, BPaL for fluoroquinolone-resistant cases, and predefined linezolid dose adjustment protocols are implemented for patients with specific genotypic profiles. This molecular feature-based precision stratification strategy is expected to further shorten treatment duration and reduce the incidence of adverse events.

## Novel antituberculosis drugs and short-course regimens

Building on mechanistic insights into drug targets and resistance, clinical translation of novel anti-tuberculosis agents and short-course regimens has delivered transformative progress over the past five years, reshaping the standard of care for both drug-susceptible and drug-resistant tuberculosis. Approved novel agents such as bedaquiline, linezolid and delamanid have seen expanding real-world application and optimized dosing strategies, while late-stage investigational drugs with novel mechanisms continue to enrich the therapeutic pipeline. Alongside therapeutic advances, short-course regimens have redefined treatment paradigms, with corresponding safety management strategies evolving in parallel. The following sections evaluate clinical application of approved agents, outline the investigational drug pipeline, and analyze clinical breakthroughs in short-course regimens as well as safety management strategies.

### Clinical application and optimization of approved new drugs

The BPaL/BPaLM regimens have shown excellent efficacy, but their safety needs to be monitored in clinical applications. Some studies are also exploring modified BPaL regimens using inhaled spectinamides instead of linezolid, aiming to maintain efficacy while reducing adverse reactions [148, 149]. In addition, potential drug resistance issues have been reported when pretomanid is used alone in some case reports, suggesting the need to be vigilant against the emergence of resistance to novel drugs [150]. Hepatotoxicity has also been a concern in regimens containing pretomanid and pyrazinamide, but in the Nix-TB and ZeNix trials, the BPaL regimen showed a favorable hepatosafety profile [151].

Real-world evidence demonstrates significant heterogeneity in the efficacy and safety profiles of BPaL and BPaLM regimens across diverse patient populations, primarily influenced by immune status, pharmacokinetic variability, and healthcare system capacity. While HIV-positive individuals achieve comparable or superior treatment success rates with these regimens, they require careful management of drug-drug interactions—particularly between bedaquiline and antiretroviral agents—and heightened vigilance for immune reconstitution inflammatory syndrome (IRIS). Pediatric populations face unique pharmacokinetic challenges due to developmental changes in drug metabolism, with limited safety data on long-term linezolid use and heightened risks of neurotoxicity and myelosuppression. In high-burden regions of Asia and Africa, outcomes are heavily dependent on healthcare infrastructure, drug supply chains, and socioeconomic support systems; genetic polymorphisms in drug-metabolizing enzymes (e.g., CYP3A4/CYP2C8) further contribute to interregional variability in bedaquiline exposure and QT-prolongation risk. Linezolid-associated hematologic toxicity and peripheral neuropathy remain the most common adverse events, with higher incidence in malnourished or anemic populations. Future efforts must prioritize population-specific dosing optimization, strengthened pharmacovigilance networks, and health economic analyses to ensure equitable global implementation of these innovative regimens while mitigating safety risks through context-adapted clinical monitoring protocols.

To reduce linezolid toxicity, researchers are exploring shorter-course and lower-dose regimens [152, 153]. For example, in the BPaLM regimen, the dose of linezolid is usually 600 mg once daily for 16 weeks [152]. However, studies have shown that even at a lower dose of 300 mg once daily, good blood drug concentrations and efficacy can be achieved, while significantly reducing the incidence of adverse reactions [154]; Linezolid is often used in combination with other novel antituberculosis drugs to enhance efficacy and may reduce its toxicity by shortening treatment duration [129, 155]. For example, in the treatment of MDR-TB patients resistant to fluoroquinolones, the "core" combination of bedaquiline, linezolid, and clofazimine is considered effective [156]. This combination has shown potential in improving treatment outcomes, especially in cases where existing treatment regimens are ineffective [156]; To overcome linezolid toxicity, researchers are developing novel oxazolidinones with better safety profiles. For example, OTB-658 is a novel oxazolidinone designed to provide higher efficacy and safety than linezolid [157]. Preclinical evaluations have shown that OTB-658 exhibits superior efficacy to linezolid in cell models and animal studies with better safety, promising to become a next-generation antituberculosis drug to address the limitations of existing treatments [157]; Studies have also found that linezolid can cross the blood–brain barrier, making it a potential candidate for the treatment of tuberculous meningitis (TBM), but further research on its pharmacokinetic characteristics in cerebrospinal fluid is needed [158].

Delamanid resistance is a growing clinical concern [159]; and resistance has been increasingly reported in Asia and Europe [39]. JDB0131 is a novel nitroimidazoxazole antituberculosis drug similar to delamanid but may have better pharmacokinetic properties and lower toxicity [160, 161]. Preventive treatment with delamanid in household contacts of MDR-TB patients is also undergoing clinical trials to evaluate its effectiveness in reducing infection risk [162].

The heterogeneous treatment efficacy, drug-related toxicities, and emerging resistance risks identified in clinical implementation indicate that future research must transition from a sole focus on "regimen shortening" to the dual priorities of "precision optimization" and "mechanistic innovation."

#### Precision management of linezolid-associated toxicity

As a cornerstone component of the BPaL and BPaLM regimens, the clinical utility of linezolid in prolonged treatment courses or vulnerable populations is constrained by severe adverse events, most notably myelosuppression and peripheral neuropathy. A foremost future objective is to establish pharmacokinetic/pharmacodynamic (PK/PD)-driven algorithms for individualized dose adjustment. Multiscale modeling of the efficacy and safety profiles of BPaL-based regimens can further refine the balance between systemic drug exposure and clearance, thereby preserving bactericidal potency while minimizing toxic effects. Clinical practice is undergoing a gradual paradigm shift from fixed high-dose regimens to a strategy of "low initial dose with tolerance-guided titration". For example, real-world implementation data from the United States demonstrate that initiating therapy at a 600 mg daily dose with subsequent dose modifications guided by adverse event occurrence significantly improves patient retention rates.

#### Regimen expansion for special populations and novel paradigms for preventive therapy

To date, clinical evidence supporting BPaL/BPaLM regimens has been largely generated from adult patients with pulmonary tuberculosis, with substantial data gaps remaining for pediatric populations, pregnant individuals, patients with extrapulmonary tuberculosis (EPTB), and those with severe comorbidities including chronic kidney disease and hepatic impairment. Future investigations will prioritize addressing these unmet needs. For instance, case series from Canada have provided preliminary confirmation of the safety and efficacy of BPaLM in the management of extrapulmonary multidrug-resistant tuberculosis, offering preliminary evidence for indication expansion. In pediatric settings, alongside the development of child-friendly formulations and accumulating pharmacokinetic data for newer antituberculosis agents, bedaquiline- and delamanid-based short-course regimens are expected to progressively replace conventional long-term injectable regimens, leading to meaningful improvements in quality of life for affected children.

### Late-stage clinical investigational new drugs

#### Next-generation diaryquinolines

TBAJ-876, as a new generation of diarylquinoline drugs, is one of the focuses of current clinical research [32, 163, 164]. Compared with BDQ, TBAJ-876 shows stronger antimycobacterial activity and may have better safety profiles [163]. A phase I clinical trial in healthy subjects evaluated the pharmacokinetics and safety of TBAJ-876. The study included single ascending dose (SAD), food effect, multiple ascending dose (MAD), and relative bioavailability parts of tablets and oral suspensions [163]. The results showed that TBAJ-876 has favorable pharmacokinetic characteristics in vivo, laying the foundation for subsequent clinical development.

#### Novel targets & mechanisms

A variety of DprE1 inhibitors have entered preclinical research, among which BTZ043 is currently in phase II clinical trials, showing excellent in vitro and in vivo activity against susceptible and multidrug-resistant TB [165, 166]. In addition, DprE1 inhibitors such as PBTZ169, TBA-7371, and OPC-167832 have also demonstrated good antituberculosis activity [165]. Multiple obstacles impede the clinical translation of DprE1 inhibitors. First, limited industrial investment constrains R&D resources and timelines. Second, formulation optimization poses challenges, with complex processes and high costs hindering scalable production. Third, patient enrollment difficulties, exemplified by the terminated phase 2a trial of PBTZ169 due to slow recruitment, further slow progress. Together, these factors form barriers to development.

GSK3036656 (ganfeborole) is a first-in-class benzoxaborole drug that exerts its effect by inhibiting *Mycobacterium tuberculosis* leucyl-tRNA synthetase. A phase IIa clinical trial evaluated its early bactericidal activity and safety in previously untreated, rifampicin-susceptible TB patients, and the results showed significant early bactericidal activity and good tolerability [167].

#### Domestic investigational agents with independent intellectual property

Several novel anti-TB candidates developed by Chinese research teams have advanced into clinical stages, enriching the global drug development pipeline. Sudapyridine, a structurally optimized derivative of bedaquiline targeting Mtb ATP synthase, exerts bactericidal activity by inhibiting bacterial energy metabolism and has entered late-stage clinical evaluation [168, 169]. TBI-166, a structural analog of clofazimine, is designed to retain potent anti-TB activity while mitigating adverse reactions such as skin discoloration associated with clofazimine. Contezolid, an oxazolidinone agent initially approved for complicated skin and soft tissue infections, has demonstrated in vitro antibacterial activity against both drug-susceptible and drug-resistant Mtb clinical isolates, supporting further repurposing evaluation for TB treatment.

JDB0131, a third-generation nitroimidazole agent, has exhibited potent early bactericidal activity, favorable oral absorption, superior pharmacokinetic profiles, and acceptable tolerability in phase 2a clinical trials, with no observed risk of QT interval prolongation [161, 170]. JDB0131 with MICs superior or equivalent to delamanid and pretomanid against drug-resistant strains, it demonstrates dose-dependent bactericidal activity in vivo, showing synergism with bedaquiline in mouse models. Preclinical PK studies confirmed favorable oral bioavailability and half-life supporting once-daily dosing. Safety profiles exhibit no hERG inhibition and no severe toxicities in animal studies, with NOAELs significantly exceeding clinical doses.

The clinical translation of DprE1-targeted agents faces two core bottlenecks: constrained oral bioavailability and the rapid emergence of drug resistance. The development of next-generation DprE1 inhibitors with improved bioavailability and higher genetic resistance barriers—represented by OPC-167832 and TBA-7371—has become an inevitable developmental trend. Meanwhile, structural modification of drug molecules, such as the design of N-(amino)piperazinyl benzothiazinone derivatives with optimized safety profiles, holds promise for overcoming the inherent limitations of current agents. More critically, DprE1 inhibitors should be deployed in combination with drugs acting on distinct targets (e.g., ATP synthase or protein synthesis machinery) to exert synergistic bactericidal effects and delay the emergence of antimicrobial resistance. Since cytochrome bd oxidase serves as a compensatory survival pathway when the cytochrome bcc-aa₃ oxidase is inhibited, dual targeting of both respiratory branches represents a promising strategy to prevent treatment failure. Our group discovered a new class of cytochrome bd oxidase inhibitors, providing candidate compounds for achieving such dual-target combination therapy [171].

##### Systematic integration of short-course regimens and drug resistance surveillance

**Fig. 4:**
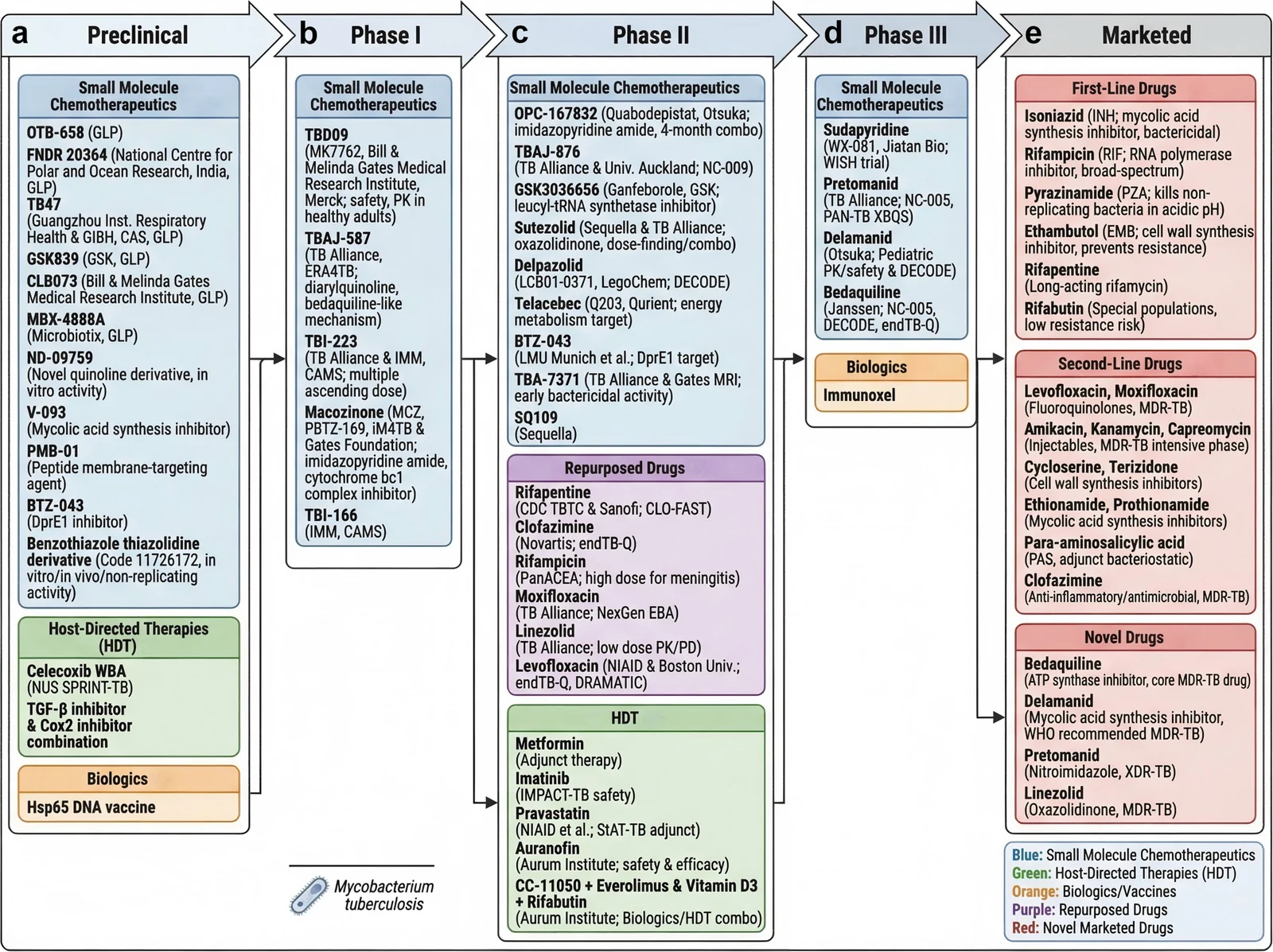
Research and development landscape of antituberculosis therapeutic agents by clinical stage and pharmacological class. Antituberculosis drug pipeline stratified by clinical stage and pharmacological class. **a** Preclinical. Lead compounds: OTB-658, GSK3036656 (ganfeborole), BTZ043, plus repurposed agents and HDT candidates. **b** Phase I. First-in-human studies assess pharmacokinetics, safety and tolerability of novel diarylquinolines and nitroimidazoles. **c** Phase II. Early bactericidal activity, optimal dosing and preliminary efficacy evaluated in drug-susceptible and drug-resistant TB patients. **d** Phase III. Large randomized controlled trials confirm regimen efficacy and safety for registration. **e** Marketed agents. Approved first- and second-line chemotherapeutics form the backbone of standard care. Agents categorized by class: small-molecule chemotherapeutics, biological agents, repurposed drugs, HDTs. INH, isoniazid; RIF, rifampicin; PZA, pyrazinamide; EMB, ethambutol; PAS, para-aminosalicylic acid; HDT, host-directed therapies; DprE1, decaprenylphosphoryl-β-D-ribofuranose 2′-epimerase

The realization of short-course tuberculosis treatment goals—namely 4–6 months of curative therapy for drug-susceptible TB and 6–9 months for drug-resistant TB—depends on the rational combination of multiple novel antituberculosis agents. Future efforts should establish an integrated developmental framework that unifies drug development, diagnostic tools, and resistance monitoring. Companion diagnostic assays should be developed in parallel with clinical trials of novel agents to enable real-time surveillance of resistance mutations such as Rv0678 and epiA, thereby supporting precision-guided pharmacotherapy. The research and development landscape of antituberculosis therapeutic agents, stratified by clinical development stage and pharmacological class, is outlined in Fig. 4.

### Clinical breakthroughs of short-course regimens

A series of clinical trials have provided strong evidence for shortening the treatment duration of drug-susceptible TB [172–174]. These studies confirmed the effectiveness of the new 4-month short-course regimen [174, 175]. For example, a 4-month regimen containing clofazimine, ethionamide, pyrazinamide, and ethambutol significantly shortened the time required for recurrent cure in preclinical studies [176]. In an open-label randomized clinical trial, this 4-month regimen was compared with the standard 6-month regimen in bacteriologically confirmed drug-susceptible TB patients to evaluate its efficacy and safety [176].

For extensively drug-resistant TB, a multicenter prospective cohort study conducted in China evaluated the safety and efficacy of three 9-month, all-oral, 5-drug regimens for the treatment of multidrug-resistant and pre-extensively drug-resistant tuberculosis. These regimens include: Regimen A: Bedaquiline + Linezolid + Moxifloxacin + Cycloserine + Pyrazinamide; Regimen B: Linezolid + Moxifloxacin + Cycloserine + Clofazimine + Pyrazinamide; Regimen C: Bedaquiline + Linezolid + Clofazimine + Cycloserine + Pyrazinamide. This study aims to provide evidence for selecting appropriate short-course all-oral regimens for multidrug-resistant and pre-extensively drug-resistant TB patients in China [145].

For latent infection prevention, the US Centers for Disease Control and Prevention has recommended shorter regimens since 2011, such as 12 weeks of combined isoniazid and rifapentine therapy, or 4 months of rifampicin monotherapy [177]. These short-course regimens have similar efficacy to the 6–9 month isoniazid regimen but better tolerability and higher treatment completion rates [177]. In rural communities in China, LTBI screening and treatment (including 6-week regimens) targeting key populations (≥ 50 years old) have shown potential cost-effectiveness advantages, contributing to the achievement of the "End TB" strategy goals [8].

For immunocompromised populations, decentralized, integrated bedaquiline-based short-course regimens have been proven effective and associated with improved HIV disease control [178]. This indicates the potential application of short-course regimens in immunocompromised populations. Immunocompromised patients often need to use multiple drugs simultaneously, leading to increased drug-drug interactions and toxicity [179]. For example, pharmacokinetic and drug-drug interaction studies of new drugs such as bedaquiline, delamanid, and pretomanid are crucial for formulating personalized dosing regimens to reduce adverse drug reactions.

**Fig. 5:**
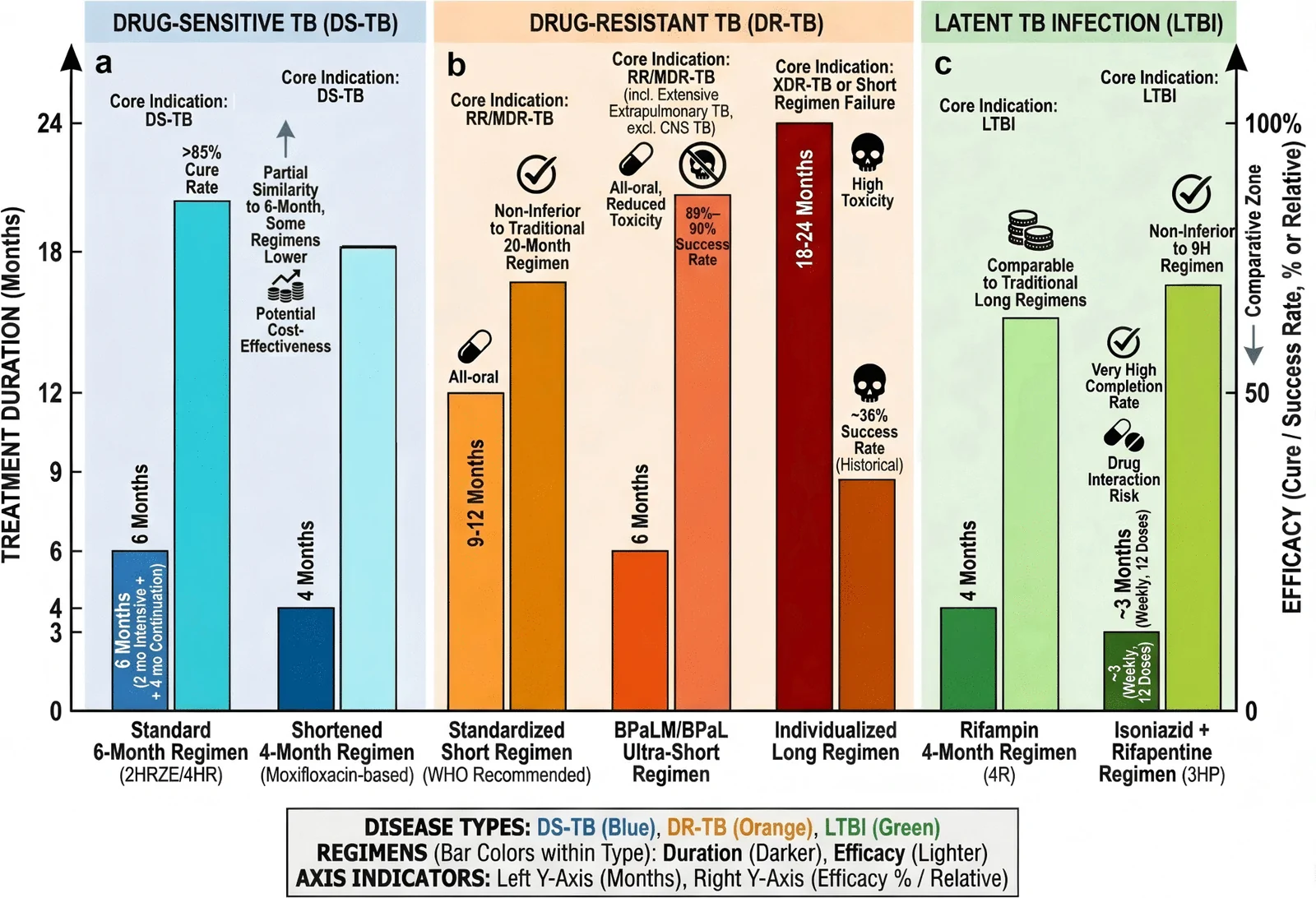
Comparative analysis of core clinical treatment regimens for tuberculosis with dual y-axis design. Comparative analysis of core TB treatment regimens with a dual y-axis design for treatment duration and relative efficacy. **a** Drug-susceptible TB. Standard 6‑month regimens vs 4‑month shortened moxifloxacin-based regimens. **b** Drug‑resistant TB. 18–24 month long‑course regimens vs 6–9 month all‑oral short‑course BPaL/BPaLM regimens. **c** Latent TB infection. 6–9 month isoniazid monotherapy vs shorter 4R and 3HP regimens. Left y-axis = treatment duration (months); right y-axis = relative treatment efficacy (percentage scale)

Clinical breakthroughs in short-course antituberculosis regimens have delivered powerful leverage for ending the global tuberculosis epidemic. Future research priorities should be centered on the following directions. First, the rational design of treatment-shortening strategies should be advanced, whereby patient stratification enables precision therapy and avoids unnecessary overtreatment. Second, the novel drug research and development pipeline needs to be strengthened: for example, combining next-generation oxazolidinone agents such as TBI-223 with existing short-course regimens holds the potential to further improve therapeutic efficacy while mitigating toxicity. Third, nanotechnology-enabled optimization of drug delivery systems can elevate drug concentrations at lesion sites, thereby shortening the overall treatment course. Fourth, global antimicrobial resistance surveillance networks should be consolidated, with artificial intelligence deployed to predict resistance evolution trends and inform the dynamic adaptation of short-course regimens. Finally, the social determinants of access to short-course regimens warrant dedicated attention to ensure that low-income countries and regions can equally benefit from this technological advancement.Driven by cross-disciplinary integration and sustained technological innovation, short-course therapeutic regimens are poised to become the standard paradigm for global tuberculosis control over the coming decade. Comparative analysis of core clinical treatment regimens across drug-susceptible TB, drug-resistant TB and latent TB infection, with a dual y-axis design concurrently presenting treatment duration and relative therapeutic efficacy, is summarized in Fig. 5.

### Side effects of tuberculosis drugs

#### Common adverse reactions and clinical management

Antituberculosis drug-induced liver injury (AT-DILI) is the most common severe adverse reaction during antituberculosis treatment, which may lead to treatment interruption and is one of the leading causes of clinical drug-induced liver injury [180, 181]. Isoniazid (INH), rifampicin (RFP) and pyrazinamide (PZA) are the major hepatotoxic agents [182, 183]. Isoniazid is regarded as one of the drugs with the strongest hepatotoxicity [184]. Its mechanism is complex, involving direct toxicity of drug metabolites, oxidative stress and immune-mediated injury [181, 185, 186]. Isoniazid is metabolized by N-acetyltransferase (NAT2) in vivo, and patients with the slow acetylator phenotype have a significantly increased risk of hepatotoxicity due to the accumulation of isoniazid and its toxic metabolites [186]. Rifampicin is a potent inducer of the CYP450 enzyme system, which can accelerate the metabolism of various drugs (including some antituberculosis drugs themselves) and increase the risk of toxic product formation. The risk of hepatotoxicity is elevated when rifampicin is used in combination with isoniazid. Pyrazinamide hepatotoxicity is dose-dependent, with a higher risk at high doses. Its mechanism may be related to mitochondrial dysfunction and cellular energy depletion [181]. Clinical manifestations may include asymptomatic elevated serum transaminases, and in severe cases, it can progress to acute liver failure [181].

Neurological adverse effects represent another major category of treatment-related toxicity. Peripheral Neuropathy is mainly caused by isoniazid, manifesting as numbness, pain or paresthesia of the hands and feet [184, 187]. Isoniazid interferes with the metabolism of vitamin B6, and supplementation with vitamin B6 can effectively prevent and treat this condition [188]. Linezolid also frequently induces peripheral neuropathy, which is associated with dosage and duration of treatment [189, 190]. Optic Neuritis is a characteristic adverse effect of ethambutol (EMB) [188]. It can lead to blurred vision, red-green color vision disturbance, and even blindness in severe cases. Its mechanism may be related to the interference with mitochondrial function of optic nerve cells as ethambutol acts as a metal ion chelator. Such injury is usually reversible but requires early detection and drug withdrawal [188]. Psychoneurological Symptoms: Cycloserine may cause anxiety, depression, psychotic reactions and even epileptic seizures [189].

Gastrointestinal reactions including nausea, vomiting, abdominal pain and loss of appetite are common adverse effects of various antituberculosis drugs [184, 187]. These reactions can usually be alleviated by adjusting the administration time, taking drugs with food or symptomatic treatment. Skin reactions such as rash, urticaria, and even more severe exfoliative dermatitis can be induced by various antituberculosis drugs [184, 187, 191]. Rifampicin sometimes causes "flu-like syndrome" or fixed drug eruption. Other adverse effects include ototoxicity and nephrotoxicity from aminoglycoside drugs such as streptomycin, amikacin and capreomycin [189], QT interval prolongation from moxifloxacin, bedaquiline and delamanid, hematological damage, and hyperuricemia induced by pyrazinamide.

#### Research progress on adverse effects and management strategies

Molecular biomarkers and pharmacogenetics enable early prediction and risk stratification of drug toxicity. For instance, isoniazid-induced hepatotoxicity is closely associated with *NAT2* gene polymorphism, and patients with the slow acetylator phenotype have a higher risk [186]. *SLCO1B1* gene polymorphism may also be related to rifampicin-induced liver injury. Novel biomarkers such as miR-122, HMGB1 and K18 have been investigated for the early prediction of antituberculosis drug-induced liver injury [181]. These molecular biomarkers provide a basis for individualized medication and risk stratification [192]. AI technology has also shown great potential in predicting antituberculosis drug-induced liver injury (TB-DILI) [193]. By integrating clinical data, biochemical indicators and genomic data, AI models can identify high-risk patients earlier and more accurately, thereby optimizing treatment management and improving patient outcomes [193]. Pharmacokinetic-Pharmacodynamic (PK-PD) studies support dose optimization: monitoring the blood concentration of antituberculosis drugs and adjusting dosages help improve therapeutic efficacy and reduce adverse reactions [194, 195]. For patients with multidrug-resistant tuberculosis, pharmacokinetic studies have found that the dosage of linezolid is weight-dependent, and high-dose linezolid calculated by body weight increases the risk of adverse reactions, especially peripheral neuropathy [190]. The research and development of a new generation of antituberculosis drugs is committed to improving efficacy and reducing side effects [196]. For example, benzothiazinones (BTZ) analogs (such as BTZ043) exert potent activity against drug-resistant *Mycobacterium tuberculosis* by inhibiting the DprE1 enzyme in bacterial cell wall synthesis, and may possess better safety profiles. Nano-drug delivery systems also provide a new approach to reduce the toxicity of antituberculosis drugs. By precisely delivering drugs to infected sites, they increase local drug concentration while reducing systemic exposure, thereby minimizing adverse effects [197].

Effective adverse effect management and monitoring strategies are crucial for the success of antituberculosis treatment [198–200]. Before the initiation of treatment, a comprehensive baseline assessment should be performed for patients, including liver and renal function, visual acuity, hearing, neurological status, as well as comorbidities and concomitant medications, to identify high-risk patients [201]. Special vigilance for the occurrence of drug adverse effects is required for patients with comorbidities such as liver disease, diabetes mellitus and HIV infection [201]. During treatment, regular clinical and laboratory monitoring should be implemented: liver function requires regular monitoring of serum transaminases and bilirubin, with frequency adjusted according to risk stratification per WHO guidelines; Routine supplementation of vitamin B6 is recommended for patients receiving isoniazid to prevent peripheral neuropathy [188]; Patients treated with ethambutol should undergo regular visual acuity and color vision tests at baseline and during treatment [188]. In the event of severe adverse reactions, suspension or adjustment of antituberculosis drug dosages may be necessary, followed by gradual drug reintroduction after symptom relief to identify the causative agent. Individualized treatment guided by Therapeutic Drug Monitoring (TDM) is particularly important for second-line drugs with greater toxicity and narrower therapeutic windows [194, 195]. Patient education, pharmacist intervention and comprehensive pharmacovigilance further support safe and effective treatment implementation [188, 199, 200, 202].

#### Drug-drug interaction risks between novel antituberculosis agents and commonly used chronic disease medications

Novel antituberculosis drugs—particularly bedaquiline, delamanid, and pretomanid—have demonstrated remarkable efficacy in the treatment of drug-resistant tuberculosis. However, their interaction risks with commonly used chronic disease medications pose substantial challenges to clinical management. These newer agents are primarily metabolized by the cytochrome P450 enzyme system, especially the CYP3A4 isoenzyme, rendering them highly susceptible to potent enzyme inducers or inhibitors and thereby triggering significant pharmacokinetic alterations [203]. In tuberculosis patients with comorbid diabetes, hypertension, cardiovascular disease, or human immunodeficiency virus (HIV) infection, such interactions may not only lead to antituberculosis treatment failure but also precipitate severe arrhythmias or hepatotoxicity [204–206].

Bedaquiline, a diarylquinoline compound with an extremely long half-life, is primarily metabolized by CYP3A4 into the less active N-monodesmethyl metabolite [203]. When bedaquiline is co-administered with potent CYP3A4 inducers such as rifampicin, its plasma concentration decreases significantly, thereby diminishing antibacterial efficacy and increasing the risk of drug resistance [203]. Conversely, co-administration with CYP3A4 inhibitors—such as certain antifungal agents or macrolide antibiotics—may result in accumulation of bedaquiline and its metabolites, prolonging the QT interval and increasing the risk of torsades de pointes, a potentially fatal arrhythmia [203, 207]. In a multicenter cohort study of patients with multidrug-resistant tuberculosis across 14 countries, the combination of bedaquiline and delamanid improved treatment success rates for complex drug-resistant strains but also underscored the necessity of close cardiac safety monitoring and vigilance for potential drug interactions [208]. Furthermore, the novel short-course regimen containing bedaquiline, pretomanid, and linezolid (BPaL) has shown favorable effectiveness in clinical practice; however, linezolid-associated hematological toxicity and potential additive toxicity with other agents still require rigorous evaluation through individualized data meta-analysis [209].

Patients with chronic conditions often require long-term polypharmacy, further compounding the complexity of drug interactions. For example, patients with diabetes frequently use oral hypoglycemic agents or insulin, and tuberculosis itself as well as antituberculosis drugs may affect glycemic control [205, 206]. Studies have demonstrated interactions between certain oral hypoglycemic agents and first-line antituberculosis drugs, which may alter the bacteriostatic and bactericidal activities of antituberculosis medications or affect therapeutic efficacy in additive, synergistic, or antagonistic manners [206]. For patients with comorbid hypertension, interactions between antihypertensive and antituberculosis drugs are equally noteworthy, particularly in patients with renal insufficiency, where altered drug clearance may lead to toxicity accumulation [206, 210]. In patients with chronic kidney disease (CKD) complicated by tuberculosis, impaired renal function and polypharmacy significantly increase the risk of adverse reactions and drug-drug interactions, with mortality rates reaching 16–37% [210]. Consequently, pharmacist-led medication review to identify clinically significant interactions is critical in this population.

Treatment of patients with HIV-tuberculosis co-infection is particularly complex due to extensive pharmacokinetic interactions between antiretroviral therapy (ART) and antituberculosis drugs [204, 211]. Rifampicin, a potent CYP450 enzyme inducer, can substantially reduce plasma concentrations of many non-nucleoside reverse transcriptase inhibitors (NNRTIs) and protease inhibitors (PIs), leading to virological failure [212, 213]. Although newer antituberculosis agents such as bedaquiline lack enzyme-inducing properties, their status as CYP3A4 substrates renders them susceptible to certain ART drugs—for example, efavirenz, a CYP3A4 inducer [203, 204]. Conversely, certain protease inhibitors, acting as CYP3A4 inhibitors, may elevate bedaquiline concentrations and increase cardiotoxicity risk [204]. Therefore, when initiating antiretroviral therapy, careful selection of drug combinations and close monitoring of drug concentrations and adverse reactions are imperative [214, 215].

Beyond pharmacokinetic interactions, additive toxicity at the pharmacodynamic level also warrants careful consideration. Both bedaquiline and delamanid carry potential QT-prolonging risks, which are compounded when co-administered with other cardiotoxic agents such as certain fluoroquinolones or antipsychotics [203, 216]. Furthermore, the hepatotoxicity commonly associated with antituberculosis drugs may be exacerbated when combined with other hepatically metabolized medications, particularly in elderly patients or those with pre-existing liver disease [207, 217]. In light of this, clinicians must comprehensively assess patients' comorbidities and medication histories when designing treatment regimens, utilizing tools such as therapeutic drug monitoring (TDM) and genotypic testing to optimize dosing, ensure efficacy, and minimize toxicity [218, 219]. Future research directions should focus on developing more precise predictive models to quantify interaction risks in multidrug regimens and provide evidence for individualized dosing [220, 221].

#### Clinical management pathways for antituberculosis drug adverse effects: hepatotoxicity discontinuation thresholds and rechallenge decision-making

Clinical management pathways for antituberculosis drug adverse effects must strictly adhere to the principles of "early recognition, prompt discontinuation, and graded rechallenge" to balance antituberculosis efficacy with hepatic safety.Current international guidelines exhibit some controversy regarding the frequency of liver function monitoring, but a general consensus supports intensive monitoring during the initial intensive phase. The American Thoracic Society (ATS) recommends baseline liver function testing for patients with high-risk factors, with immediate repeat testing upon symptom onset [222]. However, multiple studies have demonstrated that reliance on symptom monitoring alone may delay the detection of asymptomatic liver enzyme elevations; consequently, routine testing of alanine aminotransferase (ALT) and aspartate aminotransferase (AST) at week 2 of treatment is recommended [223].

Regarding discontinuation thresholds, the following criteria are widely adopted in clinical practice. Symptomatic patients: Regardless of ALT levels, all potentially hepatotoxic drugs should be immediately discontinued if hepatitis symptoms—such as nausea, vomiting, abdominal pain, or jaundice—appear in conjunction with ALT exceeding the upper limit of normal (ULN) [222, 224]. Asymptomatic patients: Immediate discontinuation is warranted if ALT or AST levels exceed three times the ULN accompanied by clinical symptoms, or exceed five times the ULN even in the absence of symptoms [222, 224]. Some guidelines suggest that consideration should be given to suspending medication and closely monitoring patients with asymptomatic but persistently elevated ALT exceeding three times the ULN 16. Notably, isolated bilirubin elevation or alkaline phosphatase elevation may indicate cholestatic liver injury and warrants equal vigilance [225].

Emerging biomarkers such as interleukin-6 (IL-6), interleukin-10 (IL-10), and glutamate dehydrogenase (GLDH) may serve as sensitive indicators for early identification of antituberculosis drug-induced liver injury (AT-DILI), facilitating the detection of hepatic damage before abnormalities in conventional enzymatic markers become apparent [226].

##### Rechallenge decision-making and regimen reconstruction

Rechallenge decision-making following drug discontinuation represents a challenging aspect of clinical management. The objective of rechallenge is to preserve the most bactericidal drugs possible—while ensuring hepatic safety—to prevent drug resistance and relapse. Rechallenge is typically initiated after liver function has normalized (ALT declines to within two times the ULN or returns to baseline levels) and clinical symptoms have resolved [224, 227].

Three main rechallenge strategies have been evaluated for safety across multiple studies:

- Sequential Reintroduction: This is the most traditional and cautious approach. Drugs are reintroduced one at a time at intervals of 3–7 days, typically in the order of rifampicin, isoniazid, and pyrazinamide [224, 227]. Liver function is monitored after each drug is added; if liver enzyme elevation occurs, the most recently added drug is identified as the causative agent and permanently excluded [227].
- Simultaneous Reintroduction: A randomized controlled trial by Sharma et al. demonstrated that simultaneous full-dose or gradual dose-escalation regimens restoring isoniazid, rifampicin, and pyrazinamide showed no statistically significant difference in recurrent hepatotoxicity risk compared with the sequential approach [227, 228]. Simultaneous full-dose rechallenge shortens treatment interruption time but requires intensive medical supervision [227].
- Gradual Dose-Escalation Simultaneous Rechallenge: Multiple drugs are administered simultaneously starting at low doses and gradually increased to full doses. A study comparing these three regimens found that gradual dose-escalation simultaneous rechallenge performed well in terms of both safety and patient adherence.

If patients experience recurrent liver injury during rechallenge, hypersensitivity or intolerance to the most recently introduced drug or the entire combination is typically diagnosed. In such cases, non-standard second-line regimens must be constructed, usually comprising ethambutol, streptomycin (or amikacin), fluoroquinolones (e.g., levofloxacin, moxifloxacin), and para-aminosalicylic acid—drugs with relatively low hepatotoxicity [229]. For patients intolerant to pyrazinamide, treatment duration must be extended to 9 months; if both isoniazid and pyrazinamide are intolerable, the course may need to be extended to 18–24 months [224, 229].

##### Management considerations for special populations

In patients with HIV-associated tuberculosis, the incidence of AT-DILI is higher and the clinical course is more complex [230, 231]. Rechallenge decisions in this population must comprehensively consider drug interactions with antiretroviral therapy (ART) and the risk of immune reconstitution inflammatory syndrome (IRIS) [231]. Studies have shown that rechallenge is generally safe when appropriately managed in high HIV-prevalence cohorts, although more frequent liver function monitoring is required [232]. Additionally, N-acetylcysteine (NAC), an antioxidant, has been explored for the treatment of AT-DILI in certain clinical trials. Although its precise efficacy in accelerating ALT normalization remains debated, it may provide additional hepatic protection in severe cases [233].

The clinical management of antituberculosis drug hepatotoxicity is a dynamic decision-making process. Physicians should develop individualized monitoring plans based on baseline risk assessments, strictly enforce discontinuation threshold criteria, and select sequential or simultaneous rechallenge strategies according to patient-specific circumstances. Looking ahead, the integration of pharmacogenomics (e.g., *NAT2* genotyping) and novel biomarkers is expected to enable more precise prediction and management of hepatotoxicity [202, 226].

Drug-induced liver injury remains the most prevalent and severe adverse event associated with antituberculosis treatment, yet conventional aminotransferase biomarkers suffer from limited specificity, which restricts their capacity for reliable early warning. Future research in this domain will be highly dependent on integrated analyses of metabolomics and microbiomics. Recent prospective studies have demonstrated that urinary metabolite profiles and gut microbial signatures enable pre-treatment stratification of high-risk individuals, and machine learning-based models have yielded markedly improved accuracy for early prediction. Furthermore, longitudinal plasma metabolomics has delineated the dynamic metabolic trajectories underlying the progression of antituberculosis drug-induced liver injury (ATB-DILI), providing novel candidate targets for the development of non-invasive early diagnostic biomarkers.

The role of genetic polymorphisms in toxicity prediction is also gaining increasing prominence. Specifically, genetic variants associated with the PXR/ALAS1/FOXO1 axis are closely correlated with liver injury risk, and multidimensional predictive models constructed by integrating electronic medical record data will serve as a pivotal tool for personalized pharmacotherapy. Beyond hepatotoxicity, hematological toxicities such as linezolid-induced thrombocytopenia can also be precisely predicted via mining real-world electronic health record data. Given the significantly elevated DILI risk among elderly patients and those with comorbid metabolic disorders, differentiated monitoring protocols tailored to these specific populations are urgently required.

**Fig. 6:**
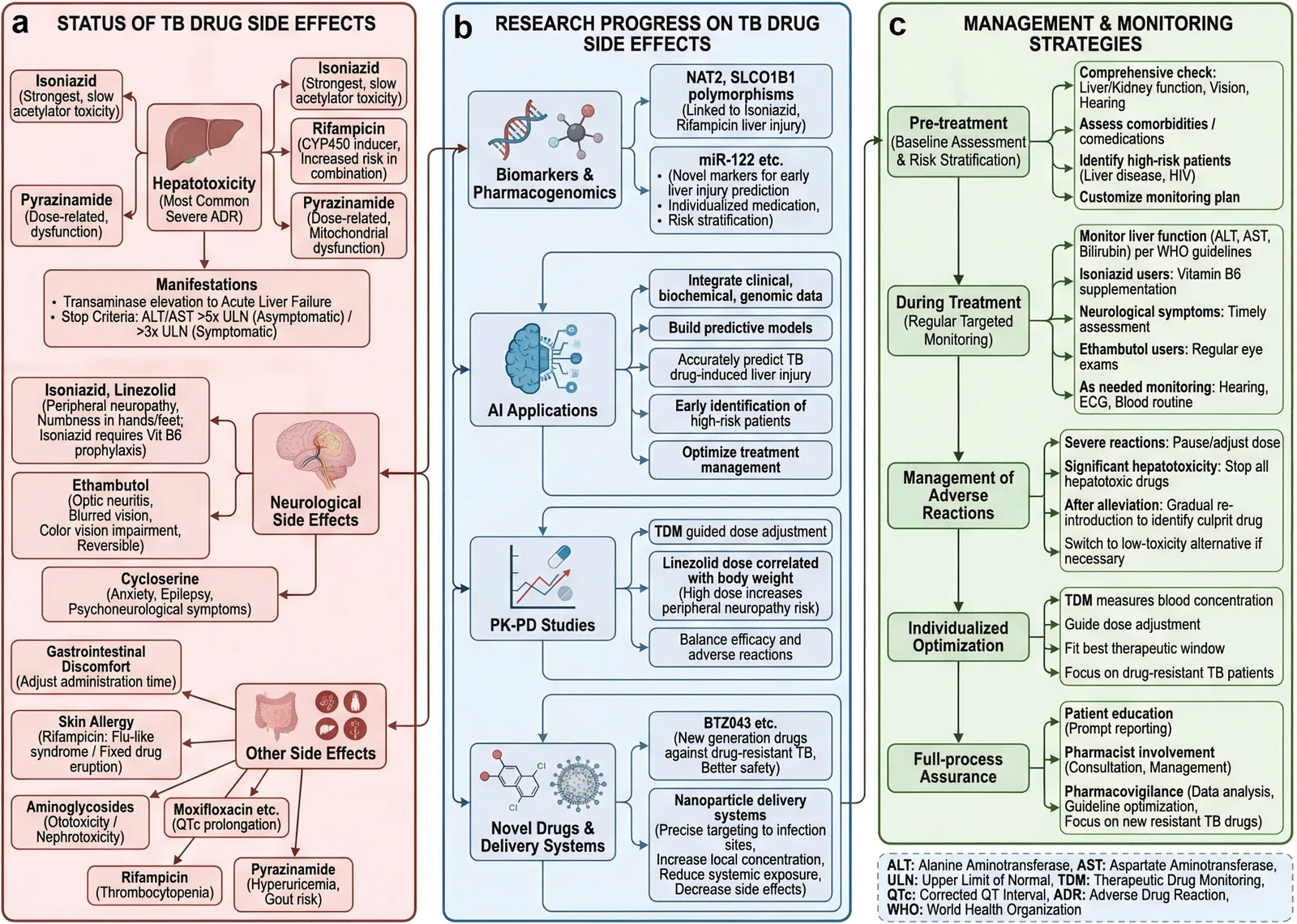
Profile of antituberculosis drug-related adverse events: status, mechanisms and management. Profile of antituberculosis drug adverse events, covering epidemiological status, mechanisms and clinical management. **a** Common adverse reactions. Hepatotoxicity (most prevalent, from isoniazid/rifampicin/pyrazinamide); other toxicities include neuropathy, optic neuritis, GI reactions, ototoxicity, QT prolongation. **b** Mechanisms & biomarkers. *NAT2/SLCO1B1* variants associate with liver injury risk; miR-122, HMGB1, GLDH are early biomarkers; PK/PD modeling and AI support precision prediction. **c** Clinical management. Baseline risk assessment, routine monitoring and TDM-guided dose adjustment form the core framework; nano-targeted delivery reduces systemic toxicity

In terms of formulation technology, nano-based drug delivery systems exhibit tremendous potential. By increasing drug concentrations at lesion sites through targeted delivery while reducing systemic exposure, these systems markedly attenuate hepatic and renal toxicity and improve patient treatment adherence. For refractory lesions such as osseous tuberculosis, antituberculosis drug-loaded bioscaffolds enable local sustained drug release, further minimizing systemic adverse effects. Regarding clinical management pathways, the integration of artificial intelligence with clinical decision support systems will drive the paradigm shift from experience-based medicine to data-driven medicine. Hybrid knowledge- and data-driven recommendation systems can automatically recommend optimal therapeutic regimens and provide early warnings of potential adverse reactions based on pathogen genotypic profiles and patient phenotypic characteristics. The full profile of antituberculosis drug-related adverse events, encompassing epidemiological status, mechanistic research progress and clinical management strategies, is illustrated in Fig. 6. The clinical development pipeline of anti-tuberculosis agents, stratified by mechanism of action and clinical trial phase, is systematically summarized in Table 1.

**Table 1:** Representative anti-tuberculosis therapeutic agents in phases I–III clinical trials

Drug/therapy name	Mechanism of action	Core NCT registration number
Phase III clinical		
Sudapyridine (WX-081)	Targeting ATP synthase, anti-tuberculosis activity comparable to bedaquiline, with lower risk of QT prolongation	NCT05824871
CC-11050 (Dovramilast)	Inhibiting phosphodiesterase 4, alleviating excessive pulmonary inflammation and granulomatous necrosis, improving lung function prognosis	NCT02968927
Metformin	Activating the AMPK pathway, enhancing macrophage autophagy and intracellular bactericidal capacity, regulating metabolic inflammation	NCT07136987
Recombinant human interleukin-2 (rhIL-2)	Promoting CD4 + T cell proliferation and Th1-type immune response, enhancing host cellular immune clearance	NCT03069534
Phase II clinical		
Sofequinolone (TBAJ-876)	Next-generation ATP synthase inhibitor with superior cardiac safety compared to bedaquiline	NCT06058299
Ganfeborole (GSK-656)	Inhibiting leucyl-tRNA synthetase (LeuRS), blocking protein synthesis	NCT03075410
Quabodepistat (OPC-167832)	Blocking mycobacterial cell wall arabinan synthesis	NCT05971602
BTZ-043	Benzothiazinone class, potently inhibiting cell wall synthesis	NCT05926466
Telacebec (Q203)	Targeting the cytochrome bc1-aa3 complex, a novel energy metabolism inhibition mechanism	NCT03563599
TBA-7371	Azaindole class, blocking cell wall synthesis	NCT04176250
Alpibectir (BVL-GSK098)	Inhibiting the EthR transcriptional repressor, enhancing bioactivation and antibacterial activity of ethionamide	NCT05473195
Pyrazofazimine (TBI-166)	Inducing reactive oxygen species accumulation and ATP depletion, with significantly lower skin pigmentation side effects than clofazimine	NCT04670120
Delpazolid (LCB01-0371)	Inhibiting ribosomal protein synthesis, with antibacterial activity superior to linezolid	NCT02836483
Sutezolid (PNU-100480)	Linezolid analog with stronger anti-tuberculosis activity	NCT01225640
N-acetylcysteine (NAC)	Clearing pulmonary oxidative stress damage, alleviating inflammation-mediated lung tissue destruction	NCT05686356
Azithromycin	Inhibiting matrix metalloproteinase activity, reducing pulmonary collagen degradation and tissue destruction, accelerating inflammation resolution	NCT03160638
Everolimus	Regulating macrophage autophagy and inflammatory balance, shortening sputum culture conversion time	NCT02968927
Auranofin	Enhancing macrophage oxidative bactericidal capacity, repurposing old drugs for tuberculosis treatment	NCT02968927
Chicoric acid	Upregulating macrophage HLA-DR and CD14 expression, promoting NO production, enhancing innate antibacterial immunity	NCT05077813
Mesenchymal stem cells (MSC)	Modulating immune-inflammatory responses, repairing lung tissue damage, reducing fibrosis and cavity formation	NCT04493918
RESP30TB	Direct pulmonary administration, increasing drug concentration in alveoli and macrophages, reducing systemic toxicity	NCT07073638
Phase I clinical		
TBAJ-587	Next-generation ATP synthase inhibitor, effective against bedaquiline-resistant strains	NCT04890535
TBI-223	Significantly reduced mitochondrial toxicity, with superior safety to existing similar drugs	NCT04865536
Macozinone (PBTZ169)	Potent benzothiazinone class, cell wall synthesis inhibitor	NCT03423030
MK-7762 (TBD09)	Highly selective inhibition of mycobacterial ribosomes, low human mitochondrial toxicity	NCT05824091
Allogeneic γδ T cells	Directly recognizing and killing intracellular/extracellular mycobacteria, enhancing innate immune clearance	NCT03575299
Invariant natural killer T cells (iNKT)	Activating innate and adaptive immunity, enhancing bactericidal effects within granulomas	NCT03551795
SQ109	Inhibiting the MmpL3 transporter, blocking mycolic acid transmembrane transport and cell wall synthesis	NCT01585636
Etoricoxib	Inhibiting inflammatory cascade reactions, alleviating lung tissue inflammatory damage	NCT02503839

## Innovative interventions and delivery technologies

While systemic chemotherapy remains the backbone of tuberculosis treatment, its efficacy is frequently limited by insufficient drug penetration at lesion sites, inter-individual pharmacokinetic heterogeneity, and severe systemic adverse effects. Precision medicine strategies and targeted delivery systems have emerged as critical complementary approaches to improve therapeutic outcomes, particularly for extrapulmonary tuberculosis and special populations. Genotypic detection, pharmacokinetic/pharmacodynamic modeling and artificial intelligence collectively form the technical foundation of precision tuberculosis medicine, while targeted delivery technologies for spinal tuberculosis, tuberculous meningitis and lymph node tuberculosis address key unmet needs for site-specific treatment. Lesion-responsive and mucosal-targeted preparations further expand the therapeutic toolkit, providing a technical framework for advancing precise, site-specific anti-tuberculosis therapy.

### Precision medicine strategies

#### Genotypic detection and molecular markers

Mutations in the *pncA* gene are the main cause of pyrazinamide resistance [234]. A study on clinical isolates of drug-resistant TB in Myanmar found that mutations in the *PncA* gene and its promoter region are highly correlated with pyrazinamide resistance. Detection of this gene mutation can be used as a genetic marker for early identification of pyrazinamide resistance [234]. Traditional phenotypic drug susceptibility testing is time-consuming and may delay the initiation of optimal treatment regimens [23, 235]. In contrast, genotypic resistance testing, such as GeneXpert MTB/RIF, can rapidly detect *Mycobacterium tuberculosis* and its resistance to rifampicin [236, 237]. Whole-genome sequencing can comprehensively identify gene mutations leading to resistance to multiple antituberculosis drugs, providing clinicians with a more detailed resistance profile to guide the selection of effective personalized treatment regimens [23, 235, 238]. For example, a multicenter study on genomic and phenotypic antimicrobial susceptibility testing of clinical isolates of drug-resistant *Mycobacterium tuberculosis* emphasized the importance of WGS in the diagnosis and management of drug-resistant TB [235].

#### Pharmacogenomics and PK/PD-guided dose optimization

In recent years, PopPK models have been used in dose optimization of various antituberculosis drugs, including isoniazid, pyrazinamide [237], and linezolid [239]. For example, one study developed a pyrazinamide PopPK model for Korean TB patients and found that old age and diabetes may affect pyrazinamide clearance, thereby guiding personalized treatment [237]. Another study evaluated the predictive ability of published PopPK models for linezolid dosing regimens in multidrug-resistant TB patients to optimize treatment and manage adherence [239]. The Model-Informed Precision Dosing (MIPD) strategy combines PopPK models and TDM to estimate patients' pharmacokinetic parameters through Bayesian prediction and adjust doses accordingly to maximize efficacy and minimize toxicity [240–243]. This method has been applied to dose individualization in the treatment of methicillin-resistant Staphylococcus aureus (MRSA) and is expected to play a greater role in TB treatment [240]. PBPK models predict the absorption, distribution, metabolism, and excretion of drugs in the body by integrating physiological and drug-specific parameters, and are particularly suitable for drug dose adjustment in pediatric and special populations [244–246]. PBPK models can simulate drug concentrations in different tissues, which is crucial for TB, a disease with limited drug penetration at lesion sites [245, 246]. For example, one study developed a PBPK model to predict the pharmacokinetics of antituberculosis drugs such as rifampicin, pyrazinamide, isoniazid, ethambutol, and moxifloxacin in healthy and infected tissues and extrapolated it to humans [246].

Automated, web-based TDM platforms are being developed to simplify implementation and provide model-based precision dosing, thereby optimizing antituberculosis drug doses [247]. Such platforms can reduce the time and professionals required, improving the accessibility of TDM in clinical practice [247]. The application of liquid chromatography-mass spectrometry (LC–MS) technology in TDM and drug metabolomics has also promoted the development of precision dosing of antituberculosis drugs [248, 249]. LC–MS can accurately measure the concentrations of antituberculosis drugs and their metabolites, providing more accurate data for dose adjustment [248].

#### Personalized regimens for special populations

A study evaluated the plasma concentrations of antituberculosis drugs in children and adolescents and found that subtherapeutic plasma concentrations of drugs are one of the main reasons for poor treatment outcomes [250]. The study emphasized the role of TDM in promoting personalized treatment and proposed that dose adjustment based on TDM results can optimize efficacy [250].

TB treatment in diabetic patients should focus on blood glucose control, as hyperglycemia can affect the absorption and metabolism of antituberculosis drugs and may increase drug toxicity [251]. Studies have pointed out that diabetes is one of the important comorbidities affecting treatment outcomes and mortality in a cohort of TB patients in Poland [252]. In addition, diabetic patients may have higher resistance to certain antituberculosis drugs [234]; Smokers may have a more severe course of pulmonary TB and reduced responsiveness to standard antituberculosis treatment [253]. For such patients, in addition to standard antituberculosis treatment, smoking cessation interventions and COPD management are needed to improve overall treatment outcomes [253].

##### Application potential of artificial intelligence in digital pathology diagnosis of tuberculosis

The application of artificial intelligence in digital pathology diagnosis of tuberculosis is undergoing a critical transitional phase from theoretical exploration to clinical practicality. Its core potential lies in the integration of whole-slide imaging technology with deep learning algorithms to address global challenges inherent in conventional microscopic examination—including high subjectivity, time-consuming procedures, and scarcity of expert resources [254, 255]. Tuberculosis, one of the world's leading infectious causes of death, relies on direct or indirect detection of *Mycobacterium tuberculosis* for definitive diagnosis. Digital pathology, by converting physical optical signals into quantifiable digital data, provides a high-fidelity analytical foundation for AI algorithms [256, 257]. Recent studies have demonstrated that hybrid approaches combining attention-mechanism residual U-Net segmentation with ensemble classification can significantly improve the accuracy of automated diagnosis, exhibiting superior performance particularly when processing complex histomorphological features [258]. This technology extends beyond pulmonary tuberculosis to complex cases involving multi-organ involvement such as lymph nodes and bone, further enhancing diagnostic robustness through multimodal data fusion [259, 260].

At the microscopic level, AI-driven analysis of Ziehl–Neelsen (ZN) stained images has emerged as a breakthrough point for improving the sensitivity of smear microscopy. Conventional ZN-stained smear screening relies on experienced microbiologists to manually identify minute acid-fast bacilli, a process highly prone to missed or misdiagnosed cases due to visual fatigue [261, 262]. By constructing large-scale training datasets comprising millions of positive and negative sample patches, AI models can automatically identify and localize mycobacteria in tissue sections with detection efficiency far exceeding human levels 1. For example, an AI development study for ZN-stained tissue sections, trained on over 260,000 positive patches and 700 million negative patches, significantly improved detection rates in samples with low bacterial load [254]. Furthermore, multivariant digital ZN staining analysis based on object detection algorithms such as Faster RCNN effectively addresses inconsistencies in color intensity caused by uneven staining thickness, making automated screening feasible in resource-limited settings [263]. Such automated systems not only reduce human error rates but also provide objective evidence for subsequent treatment decisions through standardized output results [264].

The integration of digital pathology and AI has also profoundly transformed predictive paradigms for tuberculosis drug resistance. Conventional drug susceptibility testing is time-consuming, whereas deep learning-based approaches can predict drug sensitivity in advance by analyzing subtle morphological differences in chest CT or pathological images [265]. Studies have shown that using transfer learning approaches, AI models can extract implicit features associated with drug resistance from routine imaging data, thereby enabling rapid subtyping of multidrug-resistant tuberculosis [266, 267]. This capability is critical for developing personalized treatment regimens; particularly against the backdrop of increasing drug-resistant strains, AI-assisted diagnosis can shorten the diagnostic time window and reduce the physiological, social, and economic burdens of ineffective treatment [268, 269]. Concurrently, multimodal AI models integrating genomic data with clinical parameters have further improved predictive precision, laying the foundation for precision medicine applications in tuberculosis prevention and control [270, 271].

Nevertheless, the comprehensive deployment of AI in digital pathology diagnosis faces numerous challenges, among which data standardization and model interpretability represent two core bottlenecks. Staining batch effects across different laboratories may cause shifts in image feature distributions, severely compromising model generalizability [272]. Although various stain normalization methods have been proposed, the achievement of cross-center data consistency in real clinical environments requires further validation [272]. Furthermore, the lack of transparency in "black box" models often undermines clinicians' trust in AI decisions, making the introduction of explainable artificial intelligence (XAI) technologies particularly urgent [273, 274]. By visualizing algorithm-attention regions and providing clear decision rationale, XAI not only enhances clinician-patient trust but also facilitates the discovery of potential biological markers, thereby advancing pathological knowledge [273].

#### Clinical implementation of AI in diagnosis and treatment

In the image acquisition and reconstruction stage of the MRI diagnostic pathway, AI can be used for noise reduction and artifact removal to improve image clarity [275]. A cascaded model based on incremental learning (ILCM) has been proposed to detect and locate TB lesions from chest X-ray images, and achieve precise localization of lesions through the Faster-RCNN (F-RCNN) algorithm [276]. For TB, AI can analyze subtle patterns in X-ray images to identify pneumonia or other lung abnormalities caused by *Mycobacterium tuberculosis*, improving the reliability of diagnosis [270, 277]. A robust TB diagnosis method combining Vision Transformer and principal component analysis (PCA) has achieved high-accuracy diagnosis through chest X-ray images [278]. XAI (Explainable AI) can help doctors understand the basis for specific diagnoses made by AI models, thereby enhancing doctors' trust in AI systems and promoting their clinical implementation [274, 279].

A pulmonary TB auxiliary diagnosis system based on Huawei MindSpore framework and Ascend 310 edge computing chip has achieved low-cost and high-accuracy edge-side implementation using the MobileNetV3 architecture. This deployment method enables large-scale active TB screening in communities and remote areas without relying on powerful cloud computing resources or professional radiologists [280–282]. These cases usually show radiological abnormalities on chest X-rays. For example, in a community active case finding campaign in northeastern Nigeria, ultra-portable X-ray equipment combined with AI technology was used to screen for TB symptoms and chest X-ray abnormalities, effectively identifying subclinical cases [282].

In addition to imaging applications, AI shows promising potential in digital pathological diagnosis of TB. Deep learning algorithms can automatically analyze granuloma morphology, necrosis patterns, and acid-fast bacilli (AFB) stained images in lung tissue biopsy slides, achieving high sensitivity and specificity in identifying Mtb infection and distinguishing TB granulomas from other granulomatous diseases. AI-assisted pathological analysis can reduce inter-observer variability among pathologists and improve diagnostic efficiency, particularly in resource-limited settings with limited access to experienced pulmonary pathologists. AI-assisted microscope systems have significantly improved detection efficiency and accuracy through automated identification of acid-fast bacilli (AFB) [261, 264]. A study evaluated an AI-assisted TB acid-fast bacilli smear screening system, which consists of a microscope scanner and an identification module, and showed good performance in daily practice [264].

Deep learning convolutional neural networks (CNNs) have been used to predict TB drug resistance and susceptibility based on genomic data [283]. A TB/non-TB detection and drug resistance classification diagnostic decision support system (TB-DRC-DSS) has been developed, which can detect TB, classify drug-resistant strains, and provide treatment recommendations [284]. Data-driven methods, especially machine learning models, have been used to automatically recommend personalized treatment regimens for refractory TB [285]. AI technology has also shown potential in predicting the treatment duration, adverse reactions, treatment outcomes, and drug resistance of antituberculosis treatment (ATT) [286].

While whole-genome sequencing and molecular diagnostic platforms such as GeneXpert have secured their central position in antimicrobial resistance prediction, future transformative breakthroughs will depend on the integrated deployment of spatial immunology, dynamic pharmacokinetic/pharmacodynamic modeling, and artificial intelligence-driven system-level interventions.

At the molecular diagnostic tier, the research emphasis is shifting from binary resistance categorization (susceptible versus resistant) toward quantitative prediction of minimum inhibitory concentrations. Conventional molecular assays frequently neglect heteroresistance and borderline resistance states. In contrast, emerging methodologies that combine multivariate linear mixed models with WGS data can establish more precise correlations between genetic mutations and drug susceptibility levels, enabling truly individualized dose optimization. For example, in-depth characterization of pncA mutations should not be confined to determining pyrazinamide resistance status; instead, it requires integrative assessment alongside bacterial burden and host metabolic profiles. Moving forward, targeted next-generation sequencing is poised for programmatic implementation in resource-limited settings through streamlined workflows, serving as a comprehensive tool for routine resistance prediction. Nonetheless, discrepancies between genotypic and phenotypic drug susceptibility testing—particularly for second-line agents—still necessitate calibration of algorithmic thresholds via large-scale clinical cohort studies.

Decoding the spatial immune landscape of granulomas marks a pivotal advance in understanding tuberculosis pathophysiology. The survival of *Mycobacterium tuberculosis* within granulomas relies on a sophisticated immune microenvironment, where cytokines such as TNF-α exert a critical regulatory role in balancing latent infection maintenance and bacterial replication control. Spatial biology technologies exemplified by multiplex immunofluorescence can unravel cellular heterogeneity and functional compartmentalization inside granulomas, pinpointing immunosuppressive niches that drive treatment failure. Future research will prioritize the integration of spatial transcriptomic and proteomic datasets into computational models, to predict patient responses to immunomodulatory therapies across distinct immune phenotypes. This line of investigation will not only clarify why certain patients experience relapse following standard-of-care treatment but also define actionable targets for the development of host-directed adjunctive therapies.

**Fig. 7:**
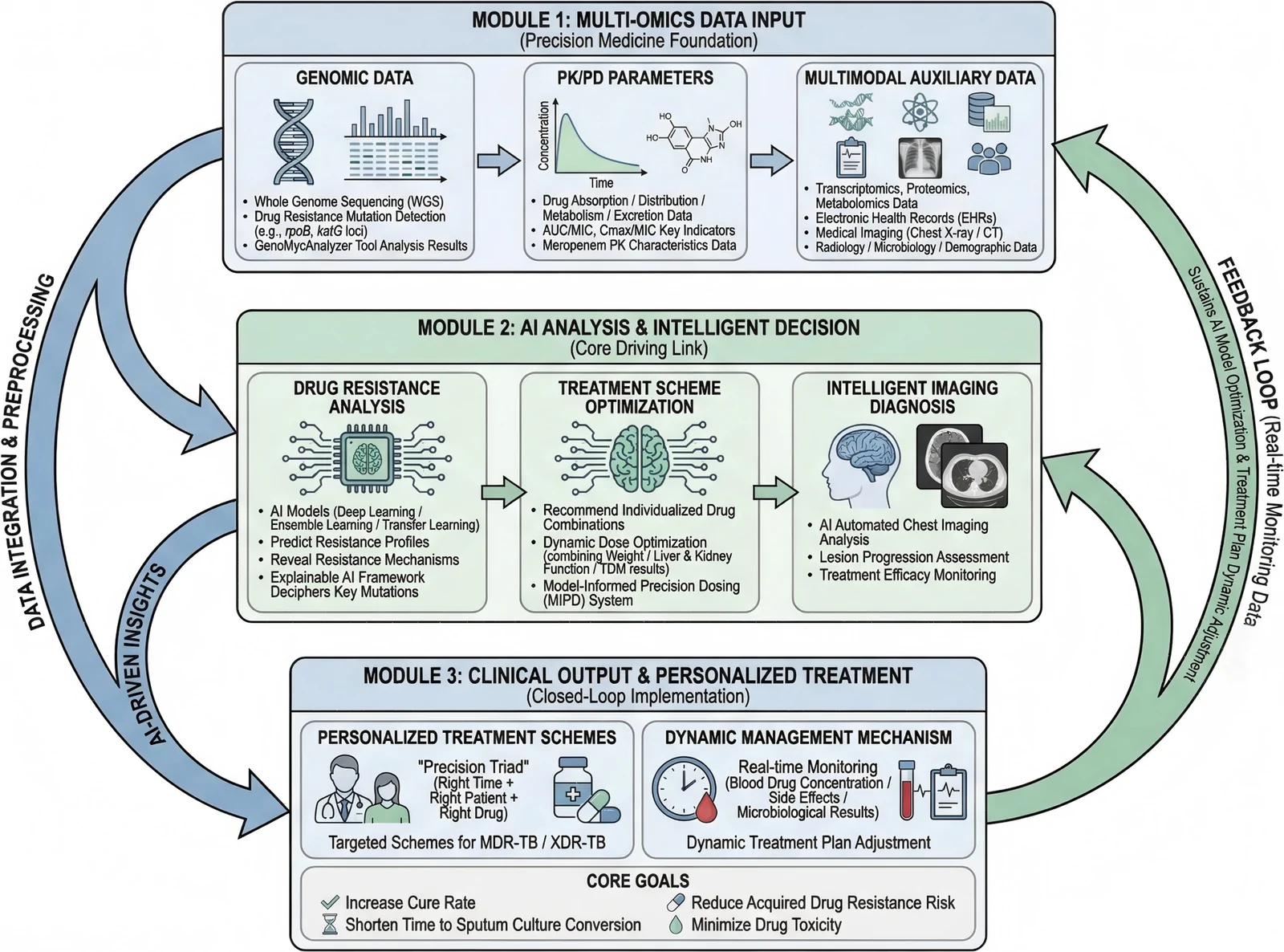
Closed-loop framework of precision tuberculosis medicine integrating artificial intelligence and multi-omics. Closed-loop precision tuberculosis medicine workflow integrating multi-omics, AI and personalized intervention. Module 1: Genomic, PK/PD and multimodal imaging/laboratory data form the precision stratification foundation. Module 2: AI enables automated resistance prediction, intelligent imaging interpretation and integrated efficacy/toxicity assessment, driving clinical decision support. Module 3: Individualized MDR/XDR-TB regimens and dynamic treatment adjustment, targeting shorter culture conversion, higher cure rate, lower toxicity and reduced acquired resistance

Future intervention strategies must prioritize the development of appropriate technologies—including portable sequencing devices and simplified AI-assisted diagnostic tools—to meet the demands of resource-poor settings. Concurrently, addressing the social determinants of tuberculosis, such as poverty, malnutrition, and residential overcrowding, represents a priority on par with biomedical interventions. The closed-loop workflow of precision tuberculosis medicine integrating multi-omics data, artificial intelligence analysis and personalized clinical intervention is presented in Fig. 7.

### Targeted delivery systems for extrapulmonary TB

#### Targeted therapeutic drugs for spinal TB

Direct delivery of drugs to spinal lesion sites can improve local drug concentrations and reduce systemic side effects. For example, biodegradable bone cement or implants can be used as drug delivery systems to continuously release antituberculosis drugs in the lesion area [287]. In patients with complex spinal TB, tumor necrosis factor-α antagonists are used to inhibit excessive inflammatory responses and improve prognosis [288]. However, the potential immunosuppressive risks need to be carefully evaluated [288]. Studies have found that the long non-coding RNA HCG18 affects the progression of spinal TB by regulating the hsa-miR-146a-5p/TGF-β1/SMADs pathway, suggesting that this pathway may become a potential therapeutic target for spinal TB [289]. In addition, elevated levels of IL-10 and TGF-β1 may impair the efficacy of preoperative antituberculosis treatment in elderly spinal TB patients [290]. Proteomic studies are identifying potential targets for rifampicin-resistant spinal TB, providing a basis for the development of innovative and effective drugs [291]. Integrative omics (genomics, transcriptomics, proteomics) approaches help achieve personalized diagnosis and treatment of TB, adjusting treatment regimens according to patients' genotypic and phenotypic characteristics [236].

Tetracycline (TC)-modified nanoparticles have been used to improve the targeted delivery potential of antituberculosis drugs in bone tissue [292]. Hydroxyapatite is the main inorganic component of bone, which is crucial for the design of bone-targeted nanodrugs [293]. Recent studies have shown that phosphazene-quercetin-alendronate composites can be used as pH-responsive bone-targeted nanodrugs for the treatment of osteoporosis, suggesting the application potential of similar strategies in bone TB [294]. Some nanopreparations can eliminate residual bacteria after chemotherapy by targeting dormant *Mycobacterium tuberculosis* in bone marrow mesenchymal stem cells, thereby reducing the recurrence rate [295]. Bone-targeted PEGylated liposomes are designed to eliminate dormant *Mycobacterium tuberculosis* in the bone marrow niche after chemotherapy, which is of great significance for preventing TB recurrence [295].

#### Targeted delivery systems for tuberculous meningitis

Dynamic imaging technology has been used to visualize the biodistribution of pretomanid in brain and lung tissues, showing preferential exposure in brain rather than lung tissues [296]. In a rabbit model of tuberculous meningitis, human equivalent doses of rifabutin achieved potential therapeutic exposure levels in relevant CNS tissues, supporting the need for further clinical evaluation [297]. The efficacy and safety of intrathecal injection of dexamethasone combined with isoniazid in the treatment of TBM are being evaluated [298]. TBM is associated with a severe inflammatory response, and adjuvant corticosteroids have been shown to have clear benefits [299, 300]. A systematic review and meta-analysis comparing steroid therapy combined with conventional therapy versus conventional therapy alone in TBM further confirmed its importance [301]. Nanotechnology shows potential in the management of pediatric TBM, such as the development of drug delivery systems with better taste-masking capabilities to promote sustained drug release and reduce dosing frequency, thereby improving patient and caregiver adherence [244].

Researchers have used dual-targeted nanoparticles loaded with tigecycline to treat intracranial infections caused by multidrug-resistant Acinetobacter baumannii; These nanoparticles can effectively cross the blood–brain barrier and enhance drug permeability in the brain [302]. Glucose transporter 1 (GLUT1) is an important receptor on blood–brain barrier endothelial cells; glucose-modified nanoparticles can cross the blood–brain barrier using the GLUT1 active transport mechanism [303]. In addition, integrins, transferrin receptors (TfR), low-density lipoprotein receptor family (LDLR), and insulin receptors are also used as targets for brain-targeted delivery [303, 304]. Cell-penetrating peptides (CPPs) are a class of short peptides that can promote transmembrane transport of drugs, and they can significantly improve the efficiency of nanodrugs in penetrating biological barriers [305]. Exosomes, as endogenous nanocarriers, are considered ideal drug delivery carriers for the treatment of brain diseases due to their unique advantages such as being able to penetrate physical barriers (including the blood–brain barrier), good biocompatibility, and low immunogenicity [306]. Biomimetic brain-targeted delivery systems (BBDDS) combine natural biomaterials and synthetic nanoparticles, which can simulate delivery mechanisms in organisms and effectively overcome the blood–brain barrier [307].

#### Targeted therapy for lymph node TB

For patients intolerant to antituberculosis drugs, lymphatic-targeted therapy aims to improve drug delivery efficiency at inflammatory sites and enhance treatment outcomes in advanced TB patients [308]. Lymphotropic therapy aims to increase drug concentrations at lesion sites, thereby enhancing efficacy and reducing systemic toxicity [308]. Clinical trials have shown that adding adjuvant therapy such as prednisolone to conventional antituberculosis treatment can significantly improve the complete response rate of lymph node TB [309]. Compared with the control group, the complete response rate was higher in lymph node TB patients who received conventional antituberculosis treatment plus prednisolone (75% [21 out of 54] vs. 26.9%) [309].

#### Lesion-responsive and mucosal-targeted preparations

The expression of specific enzymes may be upregulated at TB lesions; using these enzymes as trigger signals, carriers that can be degraded by enzyme cleavage are designed to achieve site-specific drug release [310]. Inflammatory responses at infection sites produce a large amount of reactive oxygen species. Studies have developed ROS-degradable polyethylene glycol (PEG) hydrogels for stem cell delivery and antioxidant protection [310].

Studies are developing inhaled dry powder composite preparations of moxifloxacin-metformin as a new option for pulmonary TB treatment to address the challenges posed by drug-resistant *Mycobacterium tuberculosis* strains [311]. Studies are exploring novel granulated BCG carrier delivery systems to achieve mucosal immunity, thereby enhancing vaccine efficacy.

The central focus of future advances in spinal tuberculosis management lies in the development of smart biomaterials that integrate structural support with long-acting sustained drug release. Degradable drug-loaded bone cement systems must not only mitigate local toxicity arising from initial burst drug release, but also incorporate engineered mechanisms for inflammatory cytokine regulation. Furthermore, the penetrability of nanocarriers across the granuloma microenvironment warrants dedicated investigation; size-tunable multistage delivery systems can be deployed to surmount fibrotic barriers and achieve comprehensive drug coverage of deep-seated lesions.

Tuberculous meningitis is associated with persistently high mortality. Moving beyond simple physical penetration of biological barriers, future interventional technologies will shift toward receptor-mediated active transport and biomimetic delivery strategies. Surface functionalization of nanocarriers with transferrin, lactoferrin, or targeted peptides can markedly elevate intracerebral drug concentrations by exploiting receptor-mediated endocytosis across the blood–brain barrier. For adjuvant corticosteroid therapy, robust clinical evidence corroborates its efficacy in mitigating inflammatory responses, yet future research will prioritize the development of nanoscale co-delivery systems that co-encapsulate antituberculosis agents and anti-inflammatory corticosteroids within a single vector. This platform enables spatiotemporally synchronized drug release at lesion sites, thereby maximizing synergistic therapeutic effects while minimizing the risk of systemic immunosuppression. Biomimetic membrane-coated nanoplatforms further highlight the potential to evade immune clearance and enhance brain-targeting efficiency by camouflaging as endogenous cellular components.

For lymphatic-targeted therapy, future development will capitalize on the particle size effect (typically 10–100 nm) and precise surface charge modulation to facilitate preferential accumulation of nanocarriers in draining lymph nodes via interstitial fluid flow. Stimuli-responsive carriers engineered to trigger drug release in response to the specific enzymatic or pH microenvironment of lymph nodes will be developed to improve local drug bioavailability. Regarding adjuvant prednisolone therapy, although it effectively relieves compressive symptoms, its clinical utility is constrained by adverse effects associated with long-term systemic administration. Future studies should explore locally sustained-release corticosteroid nanoformulations or nanocarrier-mediated co-delivery of immunomodulators as alternatives to systemic corticosteroid therapy; such strategies can achieve inflammatory control while preserving host immune surveillance against *Mycobacterium tuberculosis*.

**Fig. 8:**
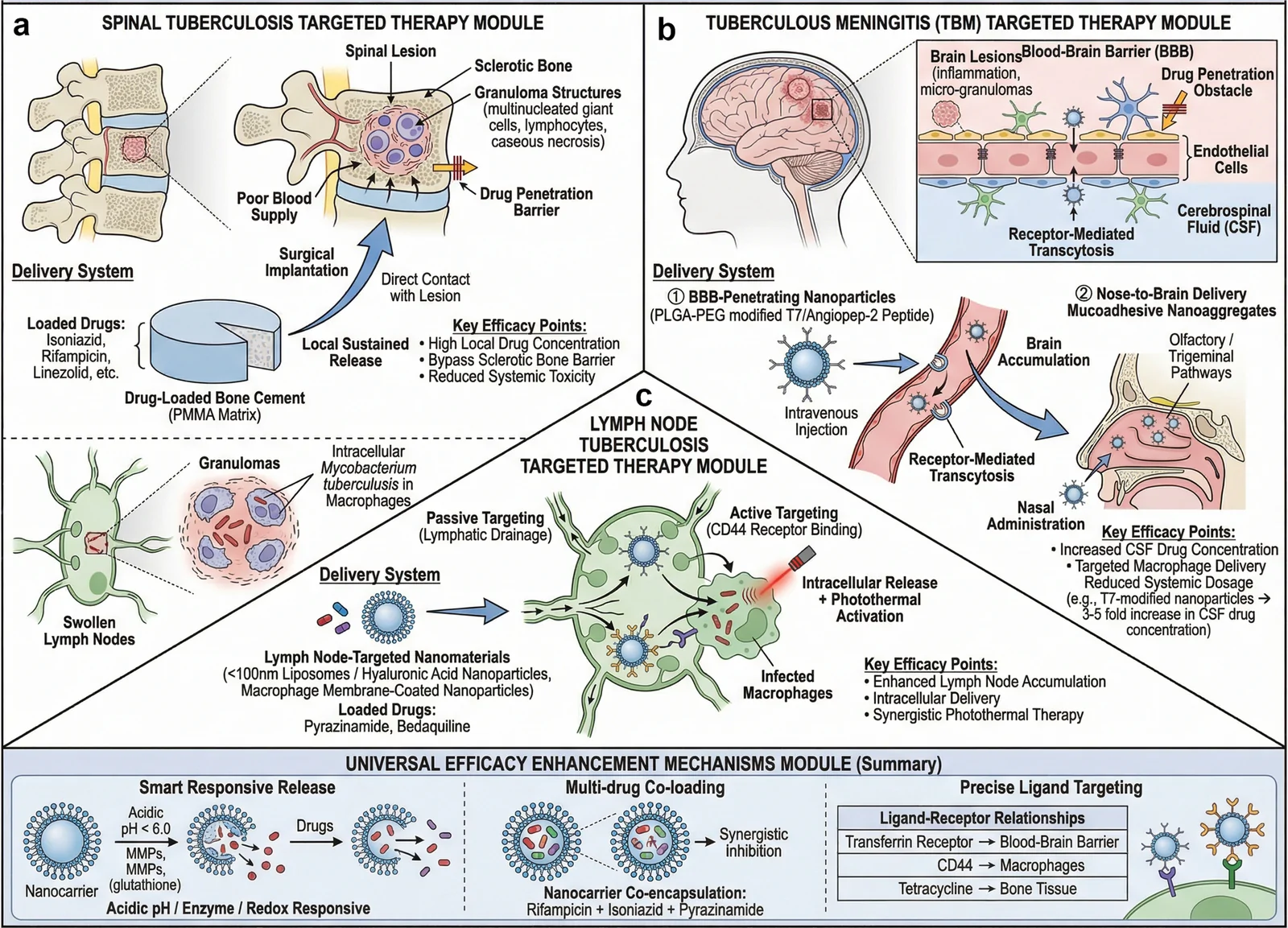
Targeted therapy mechanisms and delivery systems for extrapulmonary tuberculosis. Targeted delivery strategies for three extrapulmonary TB forms, plus universal efficacy enhancement mechanisms. **a** Spinal TB. Drug-loaded bone cement/implants enable sustained local release; bone-targeted nanoparticles enhance lesion drug accumulation. **b** Tuberculous meningitis. Functionalized nanocarriers cross the blood–brain barrier; adjuvant corticosteroids mitigate excessive inflammation. **c** Lymph node TB. Lymphotropic delivery systems enable preferential lymph node drug accumulation; adjuvant prednisolone improves response rates. Universal mechanisms: Multi-drug co-loading, ligand targeting and stimuli-responsive release collectively improve targeting precision and efficacy

Lesion-specific drug delivery vehicles represent a critical solution to the dual challenges of drug resistance and systemic toxicity. Enzyme-responsive and reactive oxygen species (ROS)-responsive nanocarriers can selectively recognize upregulated enzymes or the oxidative stress microenvironment within tuberculous granulomas, enabling precise on-demand drug release. In the domain of mucosal targeting, inhalable dry powder formulations are suitable not only for pulmonary tuberculosis treatment but also for the prevention of early extrapulmonary tuberculosis disseminated via the respiratory tract. Future prospects include the development of liposome- or polymer-based mucosal vaccine delivery systems, which can elicit dual protective mucosal and systemic immunity via dendritic cell targeting. Targeted therapy mechanisms and delivery systems for three major forms of extrapulmonary tuberculosis, alongside universal efficacy enhancement strategies, are schematically shown in Fig. 8.

## Host-directed therapy and biotherapies

Conventional antibacterial therapies that directly target *Mycobacterium tuberculosis* face inherent limitations in eliminating persistent bacteria and overcoming drug resistance, driving a paradigm shift toward host-directed therapeutic strategies that modulate host immune responses and restore immune clearance capacity. Four categories of host-directed and biological therapies have emerged as promising complementary approaches: inflammasome and autophagy regulation, immune cell subset modulation, cell therapy, small nucleic acid drugs and phage therapy. The immunopathological basis of host-directed therapy and representative pharmacological agents are elaborated first, followed by assessment of the clinical progress and translational challenges of cell-based therapies, nucleic acid therapeutics and phage therapy, with explicit stratification of developmental maturity. Core challenges in clinical translation are further outlined, alongside optimized combination strategies with conventional antibacterial agents.

### Clinical exploration of host-directed therapy

Inflammasomes are protein complexes assembled by intracellular pattern recognition receptors (PRRs), playing key roles in the host's immune response to Mtb by activating Caspase-1 to mature pro-inflammatory cytokines IL-1β and IL-18 [312]. Studies have shown that host immune responses can be regulated by targeting inflammasomes. For example, baicalein has been found to inhibit NLRP3 and AIM2 inflammasome-mediated pyroptosis in Mtb-infected macrophages by inducing autophagy [313]. In the future, inflammasomes can be used as important targets for HDT to enhance the host's antituberculosis immunity [312]. Autophagy is an important mechanism for cells to clear Mtb by forming autophagosomes to encapsulate bacteria and deliver them to lysosomes for degradation [314–316]; Ubiquitin ligase TRIM27 exerts protective immunity against TB by activating TFEB-mediated autophagic flux [317]; In preclinical models, amiodarone has been identified as a potential HDT candidate drug that can inhibit intracellular Mtb growth and enhance autophagy [318]; Ursolic acid promotes autophagy by inhibiting the Akt/mTOR and TNF-α/TNFR1 signaling pathways, thereby reducing pyroptosis and necroptosis in Mtb-infected macrophages [319]. Berberine promotes macrophage autophagy by regulating the ROS/Ca2 + axis, thereby clearing Mtb, which is particularly effective against drug-resistant TB [320]. Herp regulates the survival of H37Ra in macrophages by regulating ROS-mediated autophagy [321].

MDSCs are increased in TB patients and promote Mtb escape by inhibiting T cell responses [322, 323]. HDT can counteract the immunosuppressive effect of MDSCs and restore T cell reactivity by targeting MDSCs, such as using all-trans retinoic acid (ATRA) [322]. ATRA-loaded PLGA nanoparticles as inhaled HDT are expected to be used for targeted treatment of MDR-TB [324]. In clinical trials of TB patients, COX-2i as adjuvant therapy can reduce plasma LOX products and monocyte signals, and affect the frequency of monocyte MDSCs, which is related to TB severity [323, 325]. An important goal of HDT is to enhance the intrinsic resistance of macrophages, such as regulating macrophage function to resist Mycobacterium avium complex infection [326]. Vitamin D has potential in TB HDT, especially the pulmonary delivery of 1,25-dihydroxyvitamin D3 (calcitriol), which has shown bacteriostatic effects and reduced histopathology in mouse models, especially when used in combination with standard antituberculosis treatment [327]. However, its benefits may be limited to vitamin D-deficient patients [327]. Tamoxifen has shown promise in the treatment of TB because it acts on host immune cells, especially macrophages [328]. Some natural products are also being studied as sources of HDT, which may exert effects through multiple mechanisms such as interfering with infection, enhancing protective immune responses, and inhibiting inflammation and tissue damage [329, 330]. CXCL14 plays a key role in regulating host anti-pathogen immune responses. Studies have found that CXCL14 inhibits the survival of Mtb in macrophages by upregulating A20 to promote the production of reactive oxygen species (ROS) [331].

#### Humoral immune characteristics and BCR recognition mechanisms in tuberculosis

Although cellular immunity plays a dominant role in tuberculosis defense, humoral immunity is by no means a passive bystander. Studies have demonstrated that the B cell compartment undergoes significant alterations in patients with active tuberculosis, characterized by increased frequencies of memory B cells and decreased frequencies of naive B cells [332]. As the specific antigen recognition receptor on the surface of B cells, the B cell receptor generates diversity through V(D)J recombination and can directly recognize natively folded antigens on the *Mycobacterium tuberculosis* surface, such as lipoarabinomannan and early secreted antigenic target 6 (ESAT-6) [332, 333]. During Mtb infection, antigen binding to the BCR initiates downstream signal transduction, leading to B cell activation, proliferation, and initial differentiation into plasmablasts [332]. However, BCR signaling alone is insufficient to generate high-affinity, long-lived antibody responses; this process is highly dependent on T cell help [334]. Notably, Mtb possesses sophisticated immune evasion mechanisms that may undermine the effectiveness of humoral immunity by interfering with BCR signaling pathways or altering B cell metabolic status [335, 336]. Accordingly, one HDT strategy involves restoring the functional integrity of B cells by modulating host signaling pathways—for example, using specific kinase inhibitors or metabolic modulators to enhance the efficiency of BCR signal transduction [337, 338].

#### Central regulatory role of Tfh cells in germinal center reactions

T follicular helper cells are a specialized subset of CD4^+^ T cells characterized by the expression of CXCR5, PD-1, ICOS, and the transcription factor Bcl-6 [334, 339, 340]. In the context of tuberculosis infection, Tfh cells migrate to lymphoid follicles and interact with activated B cells to form germinal centers [334]. Within germinal centers, Tfh cells drive B cell affinity maturation and class switching through two key molecular mechanisms: first, by providing essential co-stimulatory signals through the interaction of cell surface CD40L with CD40 on B cells, and second, by secreting the cytokine IL-21 [334, 339, 340]. IL-21 not only promotes B cell proliferation but also induces the expression of activation-induced cytidine deaminase, thereby initiating somatic hypermutation and class switch recombination [334, 340]. Studies in Mtb-infected mouse models have shown that the quantity and functional status of Tfh cells are positively correlated with serum-specific IgG antibody levels [332]. Furthermore, Tfh cells ensure antibody specificity and efficiency through a "linked recognition" mechanism, whereby only B cells whose BCR specifically recognizes Mtb antigens and effectively internalizes, processes, and presents peptide antigens receive Tfh help [334, 340].

#### Intervention potential of targeting the Tfh-B cell axis in HDT strategies

Given the central role of Tfh cells in humoral immunity, HDT strategies targeting this axis hold considerable therapeutic promise. First, enhancing Tfh cell differentiation and function is critical for improving tuberculosis vaccine efficacy [334, 341]. For instance, all-trans retinoic acid (ATRA), an immunomodulatory agent, has been developed as an inhalable nanoparticle HDT formulation. Studies have found that ATRA not only modulates macrophage function but may also indirectly support Tfh cell differentiation and maintenance through effects on vitamin A metabolic pathways [324]. Second, regulating the balance between Tfh and follicular regulatory T (Tfr) cells is essential for preventing excessive inflammatory responses [339]. Tfr cells, which express FoxP3 and Bcl-6, can suppress Tfh cell function, thereby limiting the magnitude of germinal center reactions [339]. In the chronic inflammatory state of tuberculosis, moderate Tfr activity may help reduce pulmonary tissue damage, whereas excessive suppression may lead to inadequate antibody responses [342]. Consequently, the development of small-molecule drugs or biologics that precisely regulate the Tfh/Tfr ratio represents an important direction for HDT [316, 337]. Additionally, modulation of the BCR signaling pathway—for example, using specific tyrosine kinase inhibitors—may enhance B cell sensitivity to antigens and thereby increase antibody titers [333, 338].

Despite the promising outlook for HDT strategies targeting the humoral immune network, numerous challenges remain. First, the high complexity of Mtb antigens makes identification of optimal BCR targets difficult [332]. Second, the functional heterogeneity of Tfh cells across different stages of infection has not been fully elucidated, requiring more sophisticated single-cell sequencing technologies to dissect their dynamic changes [236, 343]. Furthermore, how to enhance humoral immunity without triggering autoimmune reactions remains a pressing issue [339]. Future research should focus on integrating multi-omics data to construct regulatory network models of tuberculosis humoral immunity, thereby identifying key regulatory nodes [236]. Concurrently, the integration of artificial intelligence algorithms to predict optimal HDT targets will accelerate the development of novel therapeutic strategies [343]. A deeper understanding of BCR recognition and the role of Tfh cells in the tuberculosis humoral immune network, coupled with the development of targeted HDT strategies, is expected to provide new solutions for overcoming drug-resistant tuberculosis and improving vaccine efficacy [344–346].

Novel gene expression profiles, metabolomic signatures, and host immune response-related proteins are used to assess TB infection risk and diagnose active TB [347, 348]. For example, specific biomarkers in plasma can help predict the treatment outcome and recurrence risk of pulmonary TB patients [349]. Studies are underway to validate the effectiveness of inflammatory proteins as potential biomarkers for TB and evaluate their potential as HDT targets [350].

At the core of innate immune effector mechanisms, the regulation of inflammasome-mediated pyroptosis and the enhancement of autophagic clearance constitute the dual strategic pillars of host-directed therapy. Intracellular pathogens such as *Mycobacterium tuberculosis* evade host defense by inducing NLRP3 inflammasome activation and subsequent pyroptosis in macrophages. Baicalein has been validated to markedly attenuate pyroptosis in Mtb-infected macrophages while inducing protective autophagy via inhibition of the PERK/TXNIP/NLRP3 axis. This dual regulatory mechanism underscores the unique advantages of natural products in balancing pro-inflammatory responses and cell survival. Looking forward, a key research priority is to develop small-molecule agents capable of specifically uncoupling inflammasome activation from autophagy induction, with the aim of restricting the replicative niche of pathogens while avoiding excessive tissue damage. Furthermore, amiodarone, a prototypical example of drug repurposing, has demonstrated preclinical potential to constrain mycobacterial infection by enhancing autophagy. This highlights that future HDT development should delve deeper into the immunomodulatory off-target effects of existing approved drugs, particularly those capable of penetrating the blood–brain barrier or pulmonary tissue and modulating lysosomal function, to achieve effective clearance of latent infection foci.

Phenotypic reprogramming of macrophage function and modulation of myeloid-derived suppressor cells represent another critical dimension of HDT. In chronic infections such as tuberculosis, MDSCs typically promote pathogen persistence by suppressing T cell function, making it imperative to reverse this immunosuppressive microenvironment via HDT interventions. As a classic immunomodulator, vitamin D—when delivered via macro-targeted systems such as nanoparticle-loaded all-trans retinoic acid or calcitriol—exhibits considerable potential in augmenting the bactericidal activity of macrophages while mitigating histopathological damage. Future research will focus on developing smart delivery systems that enable specific targeting of HDT agents to macrophages at lesion sites, thereby maximizing local immune effects while minimizing systemic toxicity. Meanwhile, the development of specific inhibitors or differentiation inducers targeting MDSCs holds promise for breaking the state of immune tolerance in chronic infection and restoring the efficacy of adaptive immune responses.

At the adaptive immune level, the regulation of BCR recognition and the Tfh-B cell axis in humoral immunity provides a novel interventional perspective for HDT. While conventional HDT has largely focused on innate immunity, recent studies have emphasized the importance of optimizing antibody responses by modulating the interaction between T follicular helper (Tfh) cells and B cells. Fine-tuning of BCR signaling pathways not only influences antibody affinity maturation but also determines the quality of memory B cell generation. Future HDT strategies may involve the use of small-molecule modulators to enhance Tfh cell responses to specific antigen presentation, or optimization of B cell differentiation trajectories via regulation of cytokine networks such as IL-21 and IL-6. Such a vaccine-HDT combination strategy can induce more durable and broader-spectrum protective antibody responses, and is particularly applicable to infections caused by complex pathogens for which long-term immune protection is difficult to achieve with a single vaccine.

The discovery and application of inflammatory biomarkers form the cornerstone of precision medicine implementation for HDT. Owing to the substantial interindividual heterogeneity in host responses, conventional "one-size-fits-all" therapeutic regimens often result in inefficacy or even severe adverse events in subsets of patients. Screening for dynamic inflammatory biomarkers predictive of HDT efficacy—such as specific cytokine profiles, metabolite levels, or gene expression signatures—using multi-omics technologies will be critical for future clinical translation. For instance, real-time monitoring of changes in specific inflammatory protein levels in serum enables quantitative assessment of the inhibitory effect of HDT agents on excessive inflammatory responses, thereby guiding dose adjustment. Additionally, simulation of individual host responses to HDT via digital health twin technology holds promise for identifying the optimal beneficiary population prior to clinical trials, substantially improving the success rate of research and development.

### Mechanism innovation and clinical progress of cell therapy

CAR-T cell therapy is not limited to cancer treatment but is also expanding to fields such as autoimmune diseases and viral infections [351]. For TB, it is theoretically possible to design CARs that recognize *Mycobacterium tuberculosis*-specific antigens to guide T cells to clear infected cells. Currently, CAR-T cell therapy for TB remains predominantly at the preclinical and conceptual validation stage, with no completed large-scale clinical trials for TB indication. For example, studies have explored the production of D-cycloserine (D-CS), an antibiotic that inhibits bacterial cell wall synthesis, through cell therapy to combat TB infection [352]. This suggests that genetically engineered cells can not only serve as direct killer cells but also as production and delivery platforms for biopharmaceuticals. Since Mtb mainly survives intracellularly in host cells [353, 354], TCR-T cells can recognize Mtb antigens presented on the surface of Mtb-infected cells, thereby mediating specific cytotoxic responses and clearing infected cells [355, 356]. This gives TCR-T cells unique advantages in the treatment of TB because they can directly target intracellular pathogens. For example, gene therapy using viral vectors to introduce genes encoding Mtb-specific TCRs into T cells is expected to enhance the host's immune response to Mtb [357]. An open-label, single-arm preliminary clinical study evaluated the safety and efficacy of allogeneic Vγ9Vδ2 T cell therapy in patients with multidrug-resistant tuberculosis [358]. The study enrolled 8 MDR-TB patients, and the results showed that the therapy could promote the repair of lung lesions [358]. Vγ9Vδ2 T cells are unconventional T cells that play important roles in anti-infection immunity and can recognize Mtb-infected cells without MHC presentation. This study provides preliminary clinical evidence for the application of genetically engineered T cells in TB treatment and suggests the potential of allogeneic cell therapy in MDR-TB patients [358]. Patients after CAR-T cell therapy are at risk of active TB infection [359]. A retrospective case series study described 5 patients who developed active TB after receiving CD19/CD22-targeted CAR-T cell therapy [359]. This suggests that when applying genetically engineered cell therapy to TB patients, it is necessary to fully evaluate its impact on the host immune system and potential infection risk management.

CIK cells are heterogeneous T cell populations expanded in vitro, possessing characteristics of T lymphocytes and natural killer cells, and have MHC-unrestricted ability to kill tumor cells and pathogen-infected cells. In TB treatment, CIK therapy aims to enhance Mtb-specific T cell immune responses, thereby improving the efficacy of chemotherapy [360]. For example, one study evaluated the enhancing effect of autologous CIK cell immunotherapy on the chemotherapy efficacy of MDR-TB patients. The study found that CIK therapy can improve the efficacy of conventional chemotherapy by enhancing patients' immune response to Mtb, suggesting the potential of CIK cells in addressing the treatment dilemma of MDR-TB [360]. Dendritic cells play a central role in initiating T cell immune responses, especially in inducing Th1-type immune responses to combat intracellular pathogens such as Mtb [361]. DC vaccines loaded with Mtb antigens can enhance the efficacy of BCG vaccines and support antituberculosis drug treatment [361]. One study evaluated the adoptive transfer of DC vaccines pulsed with Mtb culture filtrate antigen (CFA) in BCG-vaccinated mice to enhance BCG efficacy and support antituberculosis drug treatment. The results showed that this strategy can effectively enhance Th1 immune responses and promote the clearance of Mtb, providing new ideas for preventive and immunotherapeutic vaccine strategies [361].

MSCs therapy has advanced to phase II clinical trials, with preliminary evidence of safety and adjunctive efficacy in drug-resistant TB cohorts. MSCs can reduce the production of pro-inflammatory cytokines and increase the secretion of anti-inflammatory cytokines, thereby alleviating inflammatory damage caused by TB [362, 363]. During TB infection, MSCs can regulate the polarization of macrophages from pro-inflammatory M1 type to anti-inflammatory M2 type, which helps in the resolution of inflammation and tissue repair [364]. MSCs can inhibit the cytotoxicity of NK cells, which may help avoid excessive immune damage in some cases, but their specific role in TB needs further clarification [365]. *Mycobacterium tuberculosis* infection leads to the formation of granulomas; MSCs can help the repair and functional recovery of granulomas by promoting angiogenesis, inhibiting apoptosis, and reducing fibrosis [366, 367], and can reduce lung inflammation and fibrosis, improving lung function [368]. MSCs can secrete antimicrobial peptides and proteins, or inhibit the growth of *Mycobacterium tuberculosis* by enhancing the antibacterial ability of host immune cells [369]. However, it is worth noting that studies have also shown that MSCs may provide a "drug-tolerant and immune-privileged niche" for *Mycobacterium tuberculosis*, making the bacteria significantly tolerant to antituberculosis drugs inside MSCs [370]. One study conducted on 120 patients with multidrug-resistant pulmonary tuberculosis found that 60 patients received autologous MSCs treatment on the basis of standard antituberculosis treatment. The results showed that the treatment effect of patients receiving MSCs treatment was improved compared with the control group [371–373]. Another study also explored the application of autologous MSCs in patients with drug-resistant pulmonary TB and found that it can improve the clinical outcomes of patients [298]. A clinical study on 120 drug-resistant pulmonary TB patients aged 18 to 61 years admitted between 2009 and 2018 showed that 60 patients received MSCs treatment while receiving antituberculosis treatment, and the results indicated that the efficacy of the MSCs treatment group was better than that of the control group [371]. For extrapulmonary TB lesions such as renal TB, MSCs and their derived extracellular vesicles (EVs) also show therapeutic potential [374, 375]. An experimental study evaluated the effectiveness of MSCs in the complex treatment of experimental rabbit renal TB and found that cell therapy can promote the repair process [376].

Autologous cytokine-induced killer cell immunotherapy significantly augments the chemotherapeutic efficacy against multidrug-resistant tuberculosis. By boosting *Mycobacterium tuberculosis*-specific T cell immune responses, it improves bacteriological sputum conversion rates and accelerates the resolution of pulmonary lesions. This therapeutic modality features relatively straightforward manufacturing protocols, favorable cost-effectiveness, and avoidance of potential safety hazards associated with gene editing, rendering it particularly suitable as adjunctive therapy for MDR-TB in resource-limited settings. Dendritic cell vaccines exhibit pronounced interindividual variability, and their clinical performance is highly dependent on antigen presentation efficiency as well as the patient’s baseline immune competence; future optimization should incorporate novel adjuvants or nano-delivery systems to enhance antigen presentation efficacy. Mesenchymal stem cell-derived extracellular vesicles, as a cell-free therapeutic platform, retain the immunomodulatory properties of MSCs while circumventing the potential tumorigenic or embolic risks linked to live cell transplantation, thus representing a highly promising developmental direction. Nevertheless, the dual-edged nature of MSCs cannot be overlooked: their inherent immunosuppressive activity may precipitate reactivation of latent tuberculosis under specific circumstances, underscoring the critical importance of rigorous pre-treatment screening and judicious selection of therapeutic timing.

Chimeric antigen receptor T cells exert cytotoxicity against infected cells or secrete effector cytokines to activate macrophages via recognition of specific Mtb surface antigens. However, the pathophysiological complexity of tuberculosis—characterized by the predominantly intracellular residence of Mtb within macrophages, alongside antigenic heterogeneity and an immunosuppressive lesional microenvironment—imposes substantial constraints on the tissue infiltration and in vivo persistence of CAR-T cells.

In contrast, T cell receptor-engineered T cells recognize Mtb peptide epitopes presented in the context of major histocompatibility complex molecules, endowing them with broader antigen recognition breadth and particular suitability for targeting intracellular bacterial infections. Cutting-edge synthetic biology strategies are directed toward engineering "smart" CAR-T cells capable of sensing inflammatory microenvironmental cues and releasing cytokines in a spatiotemporally controlled manner, to reduce systemic toxicity while amplifying local bactericidal effects. Despite prevailing challenges including high manufacturing costs, potential off-target toxicity, and cytokine release syndrome, the deployment of CRISPR-based gene editing technology is expected to refine the functional potency, safety profile, and clinical accessibility of engineered T cells through multiplex genetic modifications.

No single cell-based therapy is sufficient to address the multifaceted pathophysiology of tuberculosis. The evolving paradigm favors combinatorial regimens that integrate cell therapies with novel antituberculosis agents, immune checkpoint inhibitors, or vaccines to achieve synergistic therapeutic outcomes. For instance, MSCs may function as "immune buffers" to attenuate inflammatory cascades triggered by intensive chemotherapy or CAR-T therapy, while concurrently facilitating tissue repair. Single-cell sequencing and spatial transcriptomic technologies will be leveraged to dissect the heterogeneity of the immune microenvironment in tuberculosis patients at high resolution, enabling stratification of patient subpopulations most likely to derive clinical benefit from specific cell therapies. Biomarker-driven efficacy prediction models will further enable precision tailoring of individualized therapeutic regimens.

To mitigate the long manufacturing cycle and prohibitive costs associated with autologous cell therapies, the development of allogeneic "off-the-shelf" CAR-T/natural killer cells derived from healthy donors, or induced pluripotent stem cell-derived cellular products, will emerge as a central research focus. Gene editing-mediated ablation of rejection-associated genes will enable readily available, off-the-shelf therapeutic products and substantially expand clinical accessibility. In response to the potential risks of tuberculosis reactivation and immune-related adverse events inherent to cell therapy, a robust post-infusion monitoring framework must be established. The development of controllable genetic circuits—namely safety switches that enable rapid depletion or functional suppression of infused cells upon the onset of severe adverse events—represents a pivotal technical safeguard for secure clinical translation.

### Potential of small nucleic acid drugs

siRNA therapies for TB remain at the preclinical proof-of-concept stage, with no clinical trial data available. Studies have shown that siRNA can silence TB-related genes, such as overexpressed transforming growth factor-β1 (TGF-β1), thereby improving immune regulation function [377], and nanoliposomes loaded with antituberculosis drugs and TGF-β1 siRNA have been developed to deliver drugs to macrophages infected with *Mycobacterium tuberculosis* and evaluate their therapeutic effect on TB [377]. Co-delivery of siRNA with traditional antituberculosis drugs or other small molecule drugs can achieve multi-target intervention, improve treatment effect, and delay the development of drug resistance [378].

There is a differentially expressed circRNA profile in macrophages infected with *Mycobacterium tuberculosis* [379]. For example, circ-WDR27 has been found to regulate the viability and inflammatory cytokine secretion of *Mycobacterium tuberculosis*-infected macrophages [380]. Construction of a circRNA-miRNA-mRNA competing endogenous RNA (ceRNA) regulatory network helps screen diagnostic targets for TB [381]. circRNA can regulate gene expression and immune responses by acting as a microRNA sponge, binding to proteins, or translating into proteins [382–385]. For example, cirCTRAPPC6B has been shown to enhance autophagy and M1 polarization of *Mycobacterium tuberculosis*-infected macrophages, thereby promoting the clearance of intracellular *Mycobacterium tuberculosis* [386, 387]. The development of novel vaccines based on circRNA may help improve the effectiveness and durability of vaccines, especially in cases where BCG has limited protective efficacy in adults [121, 388].

Microparticles/nanoparticles formed by polymers such as polylactic-co-glycolic acid (PLGA) have been extensively studied for antituberculosis drug delivery. PLGA nanoparticles can promote sustained drug release, reduce dosing frequency, and improve patient adherence [180, 389]. For example, PLGA nanoparticles containing first-line antituberculosis drugs such as rifampicin and isoniazid can increase lung drug concentrations and reduce systemic toxicity [189]. Solid lipid nanoparticles (SLN) have also been developed for delivering antituberculosis drugs to overcome the challenge of drug-resistant strains [390, 391]. Macrophage-targeted nanocarriers help deliver drugs directly into host cells where *Mycobacterium tuberculosis* resides [392, 393]. In TB treatment, EVs can serve as natural delivery carriers for small nucleic acid drugs to deliver drugs to infected macrophages [394].

The convergence of bioengineered exosomes and inhalable spray technology represents a cutting-edge frontier in pulmonary mucosal delivery systems, marking an evolutionary transition from conventional small-molecule drugs to precise, cell-free biotherapeutics. This strategy harnesses mesenchymal stem cell (MSC)-derived exosomes as natural nanocarriers, combined with engineered modifications and advanced nebulization formulation techniques, to overcome the complex physiological barriers of the lung—including mucociliary clearance and phagocytosis by alveolar macrophages—thereby enabling highly effective treatment of high-burden respiratory diseases such as pulmonary fibrosis, acute lung injury, and chronic obstructive pulmonary disease [395, 396].

In the therapeutic landscape of pulmonary fibrosis, bioengineered exosome sprays have demonstrated the potential to reverse pathological progression. The core pathogenesis of pulmonary fibrosis involves aberrant activation of fibroblasts, excessive collagen deposition, and dysregulation of the immune microenvironment [395, 397]. Engineered MSC exosomes can inhibit the TGF-β signaling pathway by delivering specific microRNAs, such as the miR-let-7 family or miR-29 family, thereby blocking the transformation of fibroblasts into myofibroblasts [398]. A recent study developed a sprayable bioadhesive hydrogel-loaded exosome system; although the investigation focused primarily on liver fibrosis, its underlying principle is equally applicable to pulmonary delivery. By forming an in situ gel depot, the system enables sustained release and prolonged retention of exosomes, resulting in significant improvements in histopathological parameters [399]. When translated to pulmonary applications, this approach could form a protective layer on the alveolar surface via inhalation, continuously releasing therapeutic exosomes to counteract chronic inflammation and fibrosis [400].

Furthermore, targeted reprogramming of the immune microenvironment constitutes another major application direction. Alveolar macrophages play a pivotal role in pulmonary homeostasis and disease progression [401]. Engineered exosomes can be designed to be specifically internalized by macrophages, thereby modulating their polarization state from the pro-inflammatory M1 phenotype toward the anti-inflammatory and pro-reparative M2 phenotype [402, 403]. For instance, exosomes loaded with mitochondrial protective agents can restore energy metabolism in macrophages and attenuate oxidative stress, consequently suppressing the secretion of pro-fibrotic factors [402]. This therapeutic strategy, based on metabolic reprogramming, integrates the delivery advantages of exosomes with the local high-concentration benefits of spray administration, offering a novel solution for refractory pulmonary diseases [404].

Despite its promising outlook, the clinical translation of bioengineered exosome sprays faces substantial challenges. First, scalable production and quality control remain critical issues—the extraction purity of exosomes, batch-to-batch consistency, and the efficiency of engineered modifications all require further standardization [405, 406]. Second, the stability of exosomes during the nebulization process demands more comprehensive evaluation, including the impact of nebulizer types (jet, ultrasonic, or vibrating mesh) on vesicle integrity [407, 408]. Additionally, long-term inhalation safety assessment is indispensable; the clearance mechanisms of exosomes in the lung and their potential immunogenicity risks must be clearly elucidated [396, 409].

Future research directions will focus on developing more intelligent engineering platforms, such as smart exosomes responsive to changes in the pulmonary microenvironment (e.g., pH levels or enzyme concentrations), as well as integrating microfluidic technologies to achieve efficient encapsulation and modification of exosomes [410, 411]. Meanwhile, preclinical evaluation using lung organoids and lung-on-a-chip models will accelerate the translational process of this frontier therapy [411]. By integrating the latest advances in nanomedicine, bioengineering, and pharmaceutics, bioengineered exosome-loaded spray technology is reshaping the therapeutic landscape of pulmonary diseases and providing a powerful tool for achieving precise and efficient pulmonary mucosal delivery [412, 413].

The core advantage of small interfering RNA (siRNA) in tuberculosis therapeutics lies in its capacity to precisely silence the expression of key pathogenic genes derived from either the host or *Mycobacterium tuberculosis*. The current research focus has shifted from single-gene knockdown to synergistic co-delivery strategies combined with conventional antituberculosis agents. For instance, the simultaneous delivery of siRNA and first-line antituberculosis drugs such as bedaquiline or PBTZ169 via nanocarriers enables a dual assault of "chemical bactericidal activity plus gene regulation". This multi-target intervention strategy not only directly inhibits genes essential for Mtb survival, but also markedly reverses multidrug resistance by silencing host genes encoding negative immune regulators or drug efflux pump components. Key priorities for improving therapeutic consistency include developing siRNA sequences specifically targeting the unique metabolic state of Mtb persisters, and integrating microfluidic technologies to enable high-throughput screening and assembly of lipid nanoparticles (LNPs). Furthermore, optimizing LNP formulations with ionizable lipids to enhance endosomal escape within the acidic phagolysosomal microenvironment—thereby ensuring efficient release of functional siRNA into the cytoplasm—represents a critical technical pathway to overcome the therapeutic bottlenecks posed by intracellular pathogens.

Circular RNAs (circRNAs), characterized by their unique covalently closed circular structure, exhibit exceptional stability and resistance to degradation by exonucleases, endowing them with important regulatory roles in the complex pathophysiology of tuberculosis. Future investigations should focus on constructing comprehensive circRNA regulatory networks to identify key nodal molecules, and developing novel vaccine adjuvants based on circRNA mimics or inhibitors to strengthen Mtb-specific immunological memory.

Cutting-edge engineered exosome nebulization technology represents a major breakthrough in the treatment of pulmonary diseases. Moving forward, the core tasks for clinical translation will involve the large-scale production of highly homogeneous engineered exosomes via microfluidic platforms, and the optimization of nebulization parameters to preserve the structural integrity of exosomes. Additionally, the development of smart release systems responsive to the pulmonary microenvironment (e.g., fluctuations in pH or enzyme levels) will further accelerate the bench-to-bedside translation of nucleic acid therapeutics for precision tuberculosis treatment.

### Mechanisms and preclinical breakthroughs of phage therapy

Phage therapy for TB is currently at the preclinical and early compassionate use stage, with limited clinical case reports. Phages D29 and DS6A have been shown to effectively lyse Mtb H37Rv strains [414] and exhibit the ability to clear intracellular Mtb in vitro and humanized mouse models [414, 415]. Phages can penetrate biofilms and lyse bacteria within them; Even phage-derived enzymes, such as depolymerases, can degrade biofilm matrices, thereby enhancing antibacterial effects [416]. Combined use of phages with antibiotics can overcome antibiotic resistance and enhance overall antibacterial effects [416]. Phages can be engineered to have higher lytic efficiency against Mtb or to express lytic enzymes, thereby more effectively killing bacteria [416, 417]. Some studies have also explored encapsulating phages in microparticles or hydrogels to achieve sustained release and targeted delivery [416]. In a review of preclinical studies on phage therapy for mycobacterial infections, 91% (20 out of 22 studies) reported a reduction in mycobacterial counts [418]. In mouse models, phage therapy has also been proven effective in treating infections caused by Mtb [414, 418].

Phage cocktails have bactericidal effects against a variety of Mtb strains, including those resistant to commonly used antibiotics [419]. By combining different phages, a wider range of Mtb strains can be covered, thereby improving the universality of treatment [419–421]. Combined use of phages with traditional antibiotics can produce synergistic effects, enhancing the killing of Mtb. For Mtb strains that have developed resistance to antibiotics, the addition of phages can reactivate the effectiveness of antibiotics [422]. For example, one study immobilized rifampicin and phage LysB D29 lyase on a novel UiO-66 nanocomposite for the treatment of MDR-TB infection; this "nanozyme biotherapeutic" strategy is expected to enhance the efficacy of inhaled antituberculosis treatment [422]. Phages can reduce the required dose of antibiotics, thereby reducing antibiotic-related side effects [423].

Despite its broad therapeutic promise, bacteriophage therapy for tuberculosis remains constrained by multiple bottlenecks, including the lack of standardized regulatory frameworks, technical hurdles in large-scale production, and the high cost of personalized treatment regimens. Future research priorities should be focused on the following three directions. First, standardized phage biobanks and unified quality control systems should be established to streamline the regulatory approval process. Second, in-depth characterization of the interactions between bacteriophages and the host immune system will inform optimized dosing strategies, with the goal of maximizing therapeutic efficacy while minimizing inflammatory reactions. Third, synthetic biology approaches should be deployed to engineer phages with expanded host ranges or enhanced biofilm-degrading capacity. With the deepening of cross-disciplinary integration, bacteriophage therapy is expected to gradually evolve from its current status of compassionate use into a routine clinical intervention for drug-resistant tuberculosis infections.

### Core challenges and countermeasures in translational application

Notably, biotherapeutic approaches for TB vary widely in translational maturity. Mesenchymal stem cell (MSC) therapy, primarily exerting immunomodulatory and tissue-repair effects as an adjunctive strategy, has progressed to early-phase clinical trials (phase I/II), with preliminary evidence of safety and improved sputum conversion rates in drug-resistant TB cohorts. In contrast, CAR-T cell therapy, small nucleic acid therapeutics (e.g., siRNA and antisense oligonucleotides), and engineered mycobacteriophage therapies remain predominantly at the preclinical or proof-of-concept stage, with minimal to no human clinical data available for TB specifically. Clear stratification of translational maturity is critical to avoid overestimating the near-term clinical applicability of emerging technologies.

Biotherapies, especially those based on bacterial or viral vectors, may trigger strong host immune responses and even lead to septic shock [424]. For example, bacteria-driven cancer therapy has advantages such as high specificity for targeting tumor cells, permeability, and enrichment in tumor regions, but it also has risks of high immunogenicity, potential septic shock, and inability to completely lyse cancer cells [424]. Gene editing and cell therapy also need to be wary of off-target effects and long-term safety issues [425, 426]. For example, circular RNA may have low copy number, off-target effects, and delivery difficulties in cancer treatment [427]. To address these challenges, it is necessary to optimize vector design, improve targeting specificity, and conduct rigorous preclinical and clinical safety evaluations [428]. Many novel biotherapies perform well in in vitro experiments, but their effectiveness is greatly reduced in vivo due to biological barriers, degradation, and non-specific distribution [428, 429]. For example, mRNA therapy faces challenges of poor stability and limited delivery efficiency [430, 431]. Although nanodrug delivery systems are expected to improve drug concentrations at *Mycobacterium tuberculosis* infection sites, they still need to solve the problems of biocompatibility, in vivo stability, and precise delivery in complex biological environments [429, 432, 433].

The core breakthroughs in tuberculosis biotherapeutics and host-directed therapy in the future will hinge on precise dissection of the pulmonary lesion microenvironment, intelligent reengineering of delivery systems, and adaptive evolution of regulatory paradigms. Future HDT strategies will transition from a one-size-fits-all model to precision medicine tailored to host immune phenotypes. Accordingly, integration of host transcriptomic and proteomic signatures with high-resolution lesion imaging data will serve as the cornerstone of patient stratification. By delineating disparities between pro-inflammatory and immunosuppressive phenotypes, researchers can select biotherapeutic modalities in a targeted manner. For instance, the immunomodulatory properties of mesenchymal stem cells may be preferentially indicated for patients with tissue damage driven by excessive inflammation, whereas strategies aimed at enhancing T cell infiltration or restoring macrophage bactericidal function hold greater promise for cases of bacterial persistence resulting from blunted immune responses. This biomarker-guided stratification strategy not only improves therapeutic specificity but also minimizes the potential toxic risks arising from non-specific immune activation.

In the domain of cell-based therapies, next-generation chimeric antigen receptor (CAR)-T cells or CAR-macrophages will be engineered with logic-gated circuits (e.g., AND/NOT gating) to ensure activation only upon recognition of specific pathogen antigens within a defined inflammatory microenvironment, thereby markedly reducing the risks of fratricide and off-target tissue injury. For nucleic acid-based therapeutics, CRISPR-derived antibacterial agents and antisense oligonucleotides will be optimized via macro-targeted delivery vectors to achieve efficient gene silencing of intracellular *Mycobacterium tuberculosis*, coupled with innate immune stealth designs to mitigate Toll-like receptor-mediated non-specific inflammatory responses. Bacteriophage therapy will advance toward standardized, genomically vetted lysin cocktails, delivered directly to the respiratory tract via inhalable formulations, with bioengineering strategies employed to eliminate the risk of potential horizontal gene transfer from lysogeny.

**Fig. 9:**
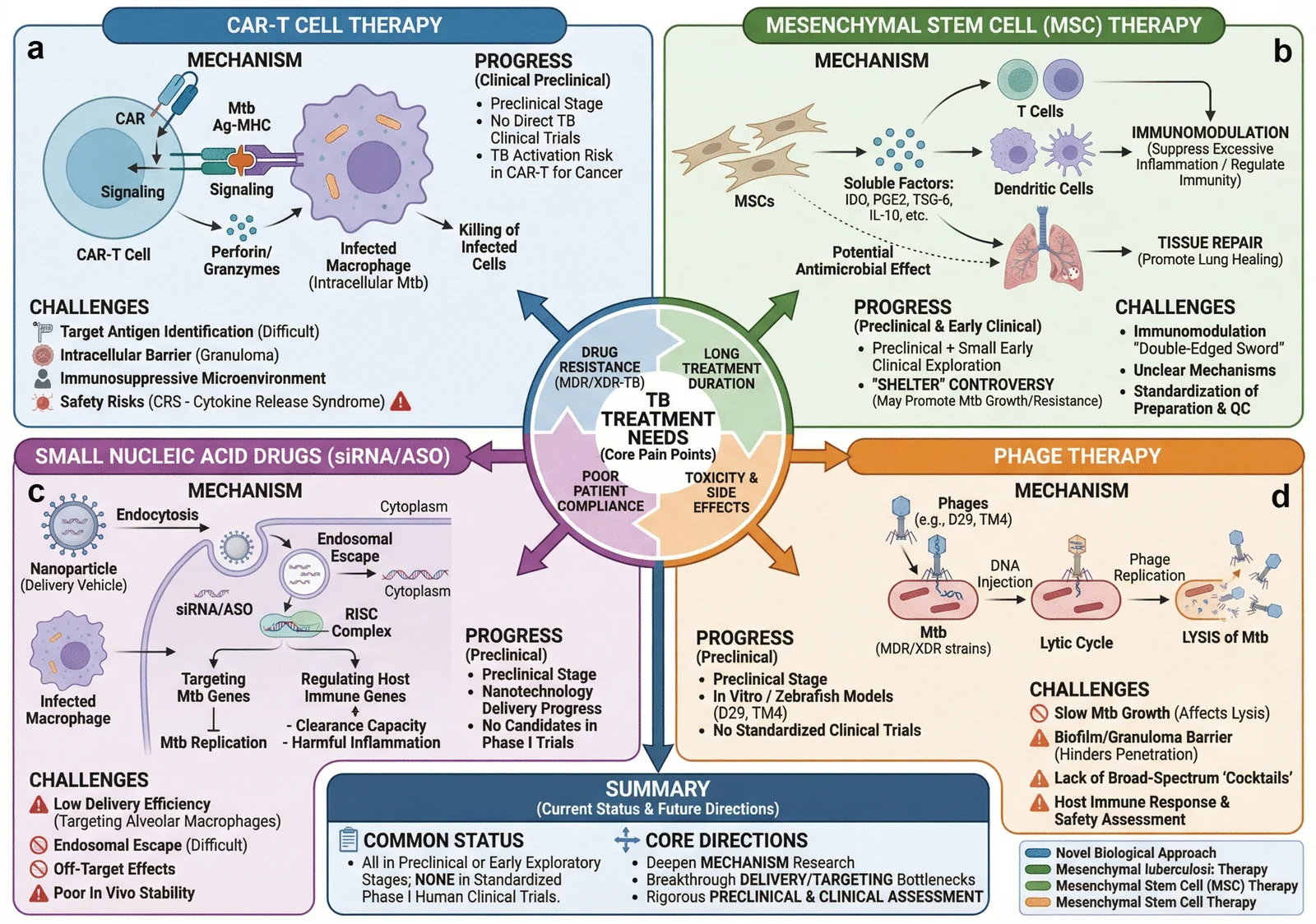
Mechanisms, development status and challenges of novel biological therapies for tuberculosis. Mechanisms, development status and core challenges of four emerging TB biotherapies. **a** CAR-T cell therapy. CAR-T cells recognize Mtb-specific antigens for targeted cytotoxicity; challenges include target identification, granuloma penetration and cytokine release risk. **b** MSC therapy. MSCs modulate macrophage polarization, suppress inflammation and promote tissue repair; challenge: potential niche effects promoting Mtb persistence. **c** Small nucleic acid drugs. siRNA/ASO silence key Mtb or host genes via macrophage delivery; challenges: endosomal escape efficiency and in vivo stability. **d** Phage therapy. Mycobacteriophages lyse Mtb and penetrate biofilms; limitations: narrow host range and limited clinical evidence

To bridge the gap between preclinical findings and clinical trial outcomes, future investigations will extensively leverage human lung organoids, granuloma-on-a-chip models, and high-resolution in vivo imaging in non-human primates. These models recapitulate the complex pathophysiological features of human tuberculosis, providing more reliable predictive platforms for evaluating the lesion penetrance and long-term safety of biotherapeutics. Meanwhile, integration of artificial intelligence with quantitative systems pharmacology models enables researchers to simulate the dynamic interactions among the host, pathogen, and therapeutic agents, and to optimize the scheduling and dosing of combination regimens. This model-guided development paradigm facilitates early identification of potential antagonistic effects or additive toxicity, thereby improving the success rate of clinical trials. Mechanisms, clinical development status and core translational challenges of four major classes of novel biological therapies for tuberculosis are comprehensively illustrated in Fig. 9.

## Novel vaccines and latent TB intervention

Therapeutic innovation alone is insufficient to curb the global tuberculosis epidemic; primary prevention through vaccination and latent tuberculosis infection intervention represents the fundamental strategy to interrupt transmission chains and reduce disease burden at source. Next-generation tuberculosis vaccines across four major technical platforms have advanced substantially in clinical development, alongside growing translation of latent tuberculosis infection prevention and intervention technologies. Recombinant BCG, protein subunit, nucleic acid and viral vector vaccines are first evaluated in terms of antigen design, immunogenic characteristics and clinical development status, followed by discussion of mucosal vaccine strategies and their potential for preventing reactivation of latent infection. The workflow of latent tuberculosis infection screening, preventive treatment and outcome assessment is further presented, alongside exploration of digital health tools for improving treatment adherence and population-level prevention efficiency.

### Clinical progress of therapeutic vaccines

Multi-stage fusion protein antigens have advantages in simulating antigen expression of *Mycobacterium tuberculosis* under different metabolic states and can induce broader immune responses [434–437]. For example, recent studies have constructed and expressed the multi-stage fusion protein SHR3 and analyzed its immunogenicity [435]. Another study developed a novel multi-stage subunit vaccine ERA005f, which provides protection against *Mycobacterium tuberculosis* by driving Th-1 and Th-17 type T cell immune responses [434]. In addition, studies have constructed novel multi-component vaccines such as EPC002, ECA006, and EPCP009 by combining multiple immunodominant antigens, which have shown good immunogenicity and protective effects in mouse models [436, 437]. Chinese research teams have also contributed to vaccine development, including clinical evaluation of the M72/AS01E subunit vaccine and exploration of BCG booster immunization strategies and novel attenuated recombinant BCG candidates [438].

ChAdOx1 85A-MVA85A is a viral vector vaccine that has entered phase II clinical trials for Prevention of Disease (POD) [439]. AdHu5Ag85A is another viral vector vaccine candidate developed by McMaster University and CanSino Biologics for Prevention of Infection (POI), currently in phase I clinical stage [439].

The B21 DNA vaccine (expressing ag85b, rv2029c, and rv1738 fusion antigens) has been shown to have significant therapeutic effects on latent TB infection, which is crucial for accelerating the decline in TB incidence in LTBI patients, especially targeting high-risk populations [440, 441]. A recent study showed that an intranasally administered DNA vaccine containing the fusion of Mtb stringent response gene relMtb and immature dendritic cell-targeting chemokine MIP-3α/CCL20 can shorten the duration of curative TB drug treatment and prevent recurrence [442]. The mRNA vaccine candidate BNT164 (developed by BioNTech SE) has entered phase IIA clinical trials, aiming to prevent infection (POI), demonstrating the application of mRNA technology in the field of preventive TB vaccines [439]. In addition, viral vector and protein/adjuvant vaccines such as ID93 + GLA-SE (developed by the US National Institutes of Health/NIAID and Qurateis) are also in phase IIA clinical trials.

VPM1002 is a recombinant BCG vaccine designed to address the limitations of traditional BCG in protecting against pulmonary TB in adults [443]. A randomized, phase 2 non-inferiority double-blind controlled trial in neonates in South Africa showed that VPM1002 is comparable to traditional BCG in terms of safety and immunogenicity, and shows good tolerability and immunogenicity in both HIV-exposed and unexposed neonates [443]. In another study, researchers selected the membrane-associated antigen Rv1513 of *Mycobacterium tuberculosis* and combined it with BCG vaccine to induce Th1-type immune responses [444].

#### Mucosal vaccines

Intranasal vaccine platforms induce immune responses directly at the respiratory mucosa—the primary portal of pathogen entry—by mimicking natural infection routes, thereby establishing multilayered long-term protective mechanisms encompassing secretory IgA (sIgA) and tissue-resident memory T cells (TRM). Unlike conventional intramuscular vaccines that primarily induce systemic humoral immunity, intranasal vaccination activates nasopharynx-associated lymphoid tissue (NALT), driving antigen-specific B cells to undergo class switching under the influence of local microenvironmental signals such as transforming growth factor-β and retinoic acid, and differentiate into sIgA-secreting plasma cells [445]. These sIgA molecules not only neutralize viruses at the mucosal surface to prevent epithelial cell entry but also restrict pathogen colonization and dissemination through immune exclusion [446, 447]. Concurrently, intranasal immunization induces CD4 + and CD8 + TRM cells that persist long-term in the nasal cavity and lung parenchyma as frontline sentinels, capable of rapidly releasing cytokines and exerting cytotoxic effects upon re-encountering pathogens without relying on lymphocyte recruitment from the circulation [448–450]. This "location is everything" immune strategy confers intranasal vaccines with distinct advantages over systemic immunity in blocking initial respiratory virus infection and subsequent transmission [451, 452].

The core mechanism by which intranasal vaccines induce durable mucosal immunity lies in the effective activation of naive T and B cells within NALT and the subsequent precise homing of effector cells to mucosal effector sites. Studies have shown that following intranasal inoculation, antigens are taken up and presented by dendritic cells in NALT, thereby activating naive CD4 + T cells—a process that shares mechanistic similarities with activation in secondary lymphoid organs but exhibits unique local characteristics [453]. Following activation, T and B cells migrate from induction sites to glandular structures and turbinate regions beneath the nasal mucosa, guided by specific chemokine receptors such as CCR10 and α4β7 integrin; these regions have been confirmed as key immune niches for antigen-specific IgA-secreting cells [445]. During this process, locally generated molecular signals such as retinoic acid and TGF-β are critical, directing B cells to undergo IgA class switching, ensuring that the resulting antibodies exist as dimers or polymers, and are transported to the mucosal surface via the polymeric immunoglobulin receptor (pIgR) to form sIgA [454]. Furthermore, state-of-the-art nanotechnology platforms—through antigen modification or the use of fluorinated nanoadjuvants—can overcome the mucus barrier and promote antigen transmucosal transport, further enhancing the efficiency of this process [455–457]. For example, fluorinated nanoadjuvants recruit precursor dendritic cells (pre-DCs) through a CCL2-driven mechanism, thereby significantly enhancing antigen presentation and subsequent immune responses [455].

The formation and maintenance of tissue-resident memory T cells represent another critical pillar of long-term protection conferred by intranasal vaccines. TRM cells reside in barrier tissues and can respond rapidly to local infection independently of the circulation, with phenotypic characteristics including high expression of markers such as CD69 and CD103 [458–460]. Intranasal vaccination induces substantial populations of antigen-specific CD4 + and CD8 + TRM cells in the nasal cavity and lungs; upon re-infection, these cells rapidly produce effector molecules such as interferon-γ (IFN-γ) and tumor necrosis factor-α (TNF-α), directly inhibiting viral replication and recruiting additional immune cells [448, 450, 461]. TRM cells induced by intranasal vaccination differ transcriptomically from those induced by natural infection, with the former typically exhibiting greater persistence and stability [460]. Moreover, CD4 + TRM cells in nasal tissue not only participate directly in antiviral defense but also play a vital coordinating role by assisting B cells in sIgA production and maintaining CD8 + TRM cell function [448, 450]. The establishment of this local memory reservoir enables the host to maintain effective cross-protective capacity against homologous and even heterosubtypic influenza viruses or SARS-CoV-2 variants [450, 462, 463].

Despite the considerable potential of intranasal vaccines, their development faces challenges including mucus barrier obstruction, antigen degradation, and the lack of safe and effective adjuvants. To address these issues, multiple novel delivery platforms have emerged. For instance, albumin-binding technology or FcRn-mediated transport mechanisms can significantly improve the efficiency of antigen transepithelial absorption [457, 464]. Multivalent antigen display platforms—including nanoparticles, liposomes, and self-assembling proteins—not only protect antigens from enzymatic degradation but also enhance immunogenicity through multivalent B cell receptor cross-linking [465–467]. Additionally, pre-existing immunity-boosting strategies—such as fusing the receptor-binding domain (RBD) with hemagglutinin, or using unadjuvanted proteins for mucosal booster immunization—can reactivate memory B cells in peripheral lymph nodes, converting them into mucosal effector cells and thereby inducing high levels of sIgA without provoking strong inflammatory responses [468, 469]. Preclinical and early clinical studies have shown that intranasal vaccines based on adenoviral vectors, live attenuated viruses, or recombinant proteins are significantly superior to intramuscular vaccines in inducing mucosal neutralizing antibodies and TRM responses. Particularly against highly transmissible SARS-CoV-2 variants such as Omicron, intranasal booster immunization effectively reduces viral load and blocks transmission [452, 454, 463]. Looking ahead, precise spatiotemporal regulation of vaccine components, combined with deeper understanding of the mucosal immune microenvironment, is expected to enable the development of next-generation intranasal vaccines with broader specificity and durability [447, 451].

Intranasally administered nanoparticle vaccines can effectively elicit protective immunity against Mtb by co-delivering antigens and mucosal adjuvants, with demonstrated efficacy in reducing bacterial load in mouse models of pulmonary TB [470, 471]. Recombinant Lactiplantibacillus plantarum-vectored vaccines carrying Mtb antigens leverage the mucosal colonization ability of probiotics to induce sustained mucosal and systemic immune responses, providing a promising oral mucosal vaccine platform [472]. Researchers are also exploring mucosal delivery devices for live attenuated TB vaccines such as BCG, aiming to optimize respiratory mucosal immune priming [473]. Despite promising preclinical data, clinical translation of mucosal vaccines faces challenges including standardization of delivery devices, durability of mucosal immune memory, and safety assessment in immunocompromised populations.

The future development of tuberculosis vaccines will no longer be confined to the simple concatenation of conventional antigens. Instead, the field is evolving toward the precise induction of long-lived memory T cells, breaching of mucosal immune barriers, and the implementation of personalized immune modulation via novel delivery technologies.A core challenge in the development of multistage fusion protein subunit vaccines lies in overcoming the insufficient immunogenicity of single antigens while avoiding pathological damage caused by excessive immune responses. Accumulating evidence indicates that multistage combination vaccines incorporating antigens expressed during the early, latent, and reactivation phases of infection can elicit more comprehensive activation of the host immune system. For instance, a recombinant BCG vaccine (rBCG-ECD003) composed of defined antigens including ESAT-6, CFP-10, and nDna7 has conferred remarkable long-term immune protection in BALB/c mouse models, mediated by the induction of robust Th1-type immune responses and sustained stability of the memory T cell pool. Furthermore, multi-epitope vaccines (e.g., PP13138R) constructed via bioinformatics-based reverse vaccinology, which integrate epitopes targeting B cells, cytotoxic T lymphocytes (CTLs), and helper T lymphocytes (HTLs), have demonstrated the potential to trigger comprehensive immune responses in in vitro immunological assessments. Another study has shown that the fusion protein SHR3, when formulated with a specific adjuvant, markedly enhances immunogenicity, highlighting that the synergistic optimization of antigen combinations and adjuvant systems is critical to improving the efficacy of subunit vaccines. Nevertheless, subunit vaccines generally suffer from relatively weak capacity to induce cellular immunity. Future research should prioritize the exploration of nanoparticle-based adjuvant technologies to improve antigen delivery efficiency and prolong the duration of immune stimulation.

**Fig. 10:**
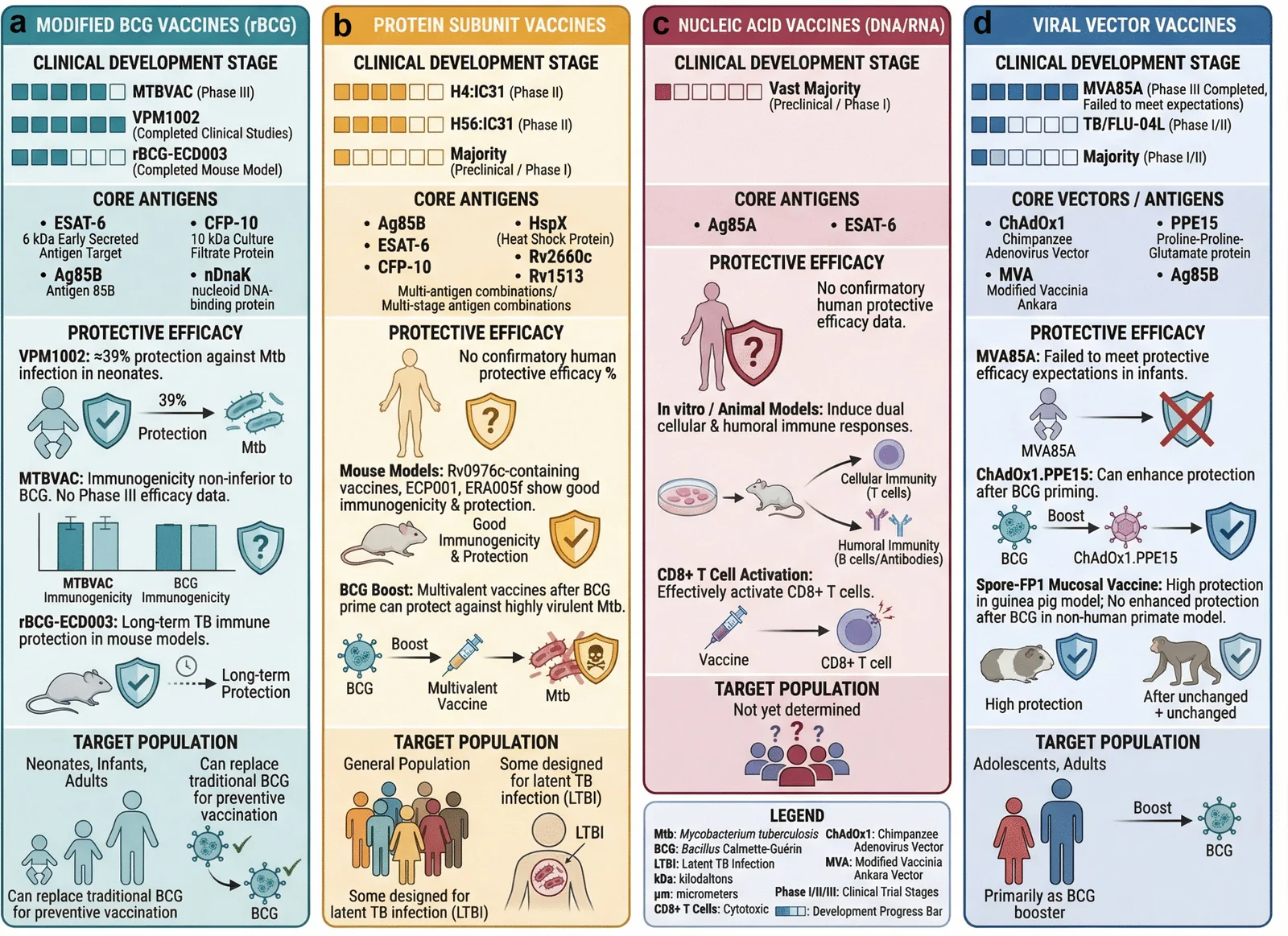
Clinical development and protective characteristics of next-generation tuberculosis vaccine candidates. Clinical status, antigen design and protective features of four next-generation TB vaccine classes. **a** Recombinant BCG. Engineered BCG expressing multistage antigens preserves safety while enhancing adult pulmonary TB efficacy; VPM1002 is a late-stage candidate. **b** Protein subunit. Multistage fusion proteins with adjuvants induce balanced Th1/Th17 responses; M72/AS01E and ERA005f are in mid-to-late clinical stages. **c** Nucleic acid. DNA/mRNA vaccines encode conserved Mtb antigens for robust CD8⁺ T cell activation; B21 and BNT164 are in early-to-mid development. **d** Viral vector. Adenoviral/MVA vectors induce strong cellular and humoral immunity; ChAdOx1 85A-MVA85A is in phase II trials

As an optimized iteration of the conventional Bacillus Calmette–Guérin (BCG) vaccine, recombinant BCG vaccines are designed to preserve the favorable safety profile of BCG while enhancing protective efficacy against adult pulmonary tuberculosis and latent infection. Going forward, the research and development of recombinant BCG will place greater emphasis on defining correlates of protection, to enable more accurate prediction of vaccine efficacy in clinical trials.The rise of mRNA vaccine technology has brought revolutionary opportunities for tuberculosis vaccine development. Nonetheless, the intrinsic instability of mRNA molecules and their limited in vivo delivery efficiency remain major technical hurdles, and optimization of lipid nanoparticle (LNP) delivery systems will be a critical determinant of successful clinical translation. With the advancement of delivery technologies, mRNA vaccines are expected to become the platform of choice for rapid immunization interventions in high-risk populations.Mucosal vaccines are regarded as a promising strategy for preventing the reactivation of latent infection, as they can establish immune barriers directly in the respiratory tract—the primary portal of entry for *Mycobacterium tuberculosis*. However, the clinical translation of mucosal vaccines faces substantial challenges, including antigen degradation at mucosal surfaces, the biocompatibility of delivery systems, and potential safety risks such as the induction of adverse pulmonary inflammation. Future investigations should focus on developing safe and efficacious mucosal adjuvants and novel nanocarriers to prolong antigen retention time and enhance uptake efficiency in mucosal tissues, while rigorously evaluating their long-term safety profiles. Clinical development status, antigen composition and protective characteristics of four major classes of next-generation tuberculosis vaccine candidates are summarized in Fig. 10.

### Clinical translation of preventive intervention technologies

A 2021 study published in a Lancet sub-journal showed that DNA of the *Mycobacterium tuberculosis* complex can be detected in CD34-positive peripheral blood mononuclear cells of asymptomatic TB contacts [474]. This cross-sectional study conducted in Ethiopia suggests that CD34-positive hematopoietic stem cells may serve as a niche for MTBC during latent infection, providing a new direction for understanding the mechanism of LTBI and developing novel diagnostic methods [474].

Long-acting products have great potential in TB preventive treatment. For example, an ideal long-acting treatment regimen may include a single dose to achieve “one-shot cure” for latent TB. For the treatment of LTBI, the US Centers for Disease Control and Prevention (CDC) has recommended shorter regimens since 2011, such as 12 weeks of combined isoniazid and rifapentine therapy, or 4 months of rifampicin monotherapy [177]. These short-course regimens have similar efficacy to the 6–9 month isoniazid regimen but better tolerability and higher treatment completion rates [177]. In rural communities in China, LTBI screening and treatment targeting key populations (≥ 50 years old) have shown potential cost-effectiveness advantages, contributing to the achievement of the “End TB” strategy goals [8].

Interventions combining electronic pillboxes and remote adherence monitoring have significantly improved the treatment success rate of TB patients in Tibet, which is a promising strategy for resource-poor regions [475]. In Wuhan, China, the implementation of the digital TB treatment monitoring system (e-PSS) has significantly improved patients' medication adherence and treatment success rate [476]. In community management in Yiwu City, digital tools have also been proven to improve medication adherence in pulmonary TB patients [477]. Mobile health applications are widely used in the health management of TB patients, including medication reminders, health education, and follow-up support [478]. These tools can help patients better adhere to treatment regimens and reduce loss to follow-up [479]. One study aims to evaluate the impact of “m-teaching” on the knowledge, attitudes, and practices of digital health utilization in TB prevention and control among active TB cases and their close contacts [480]. Telemedicine platforms help improve the continuity of care for TB patients by providing remote consultation, monitoring, and support, especially in geographically remote or medically underserved areas [478]. Digital health technologies are also used for TB monitoring to assess their consistency with World Health Organization recommendations [481].

Future research and translational priorities should be centered on three core directions: the clinical translation of non-invasive diagnostic technologies based on stem cell-associated mechanisms, regulatory approval of nanocarrier-mediated long-acting vaccine and drug delivery systems, and the construction of an artificial intelligence-driven closed-loop digital management system covering the entire disease course.

Currently, widely adopted diagnostic modalities, including the tuberculin skin test (TST) and interferon-gamma release assays (IGRAs), only reflect systemic immunological memory. They fail to discriminate between latent tuberculosis infection and active tuberculosis, nor can they predict individual risk of disease progression. Molecular detection assays targeting CD34 + cells are poised to address this unmet clinical need. For example, optimized polymerase chain reaction techniques or targeted probes against specific bacterial gene sequences within CD34 + cells enable quantitative assessment of the latent bacterial reservoir burden. Furthermore, machine learning-aided identification of LTBI-associated host gene expression profiles—represented by novel biomarkers such as MORN3 and LLGL2—can further improve diagnostic sensitivity and specificity. Future investigations should prioritize resolving challenges related to the accessibility of sample collection (e.g., development of microsample blood detection kits) and assay standardization, to promote their transition from laboratory research to routine clinical screening, particularly for immigration health screening and surveillance of high-risk populations.

**Fig. 11:**
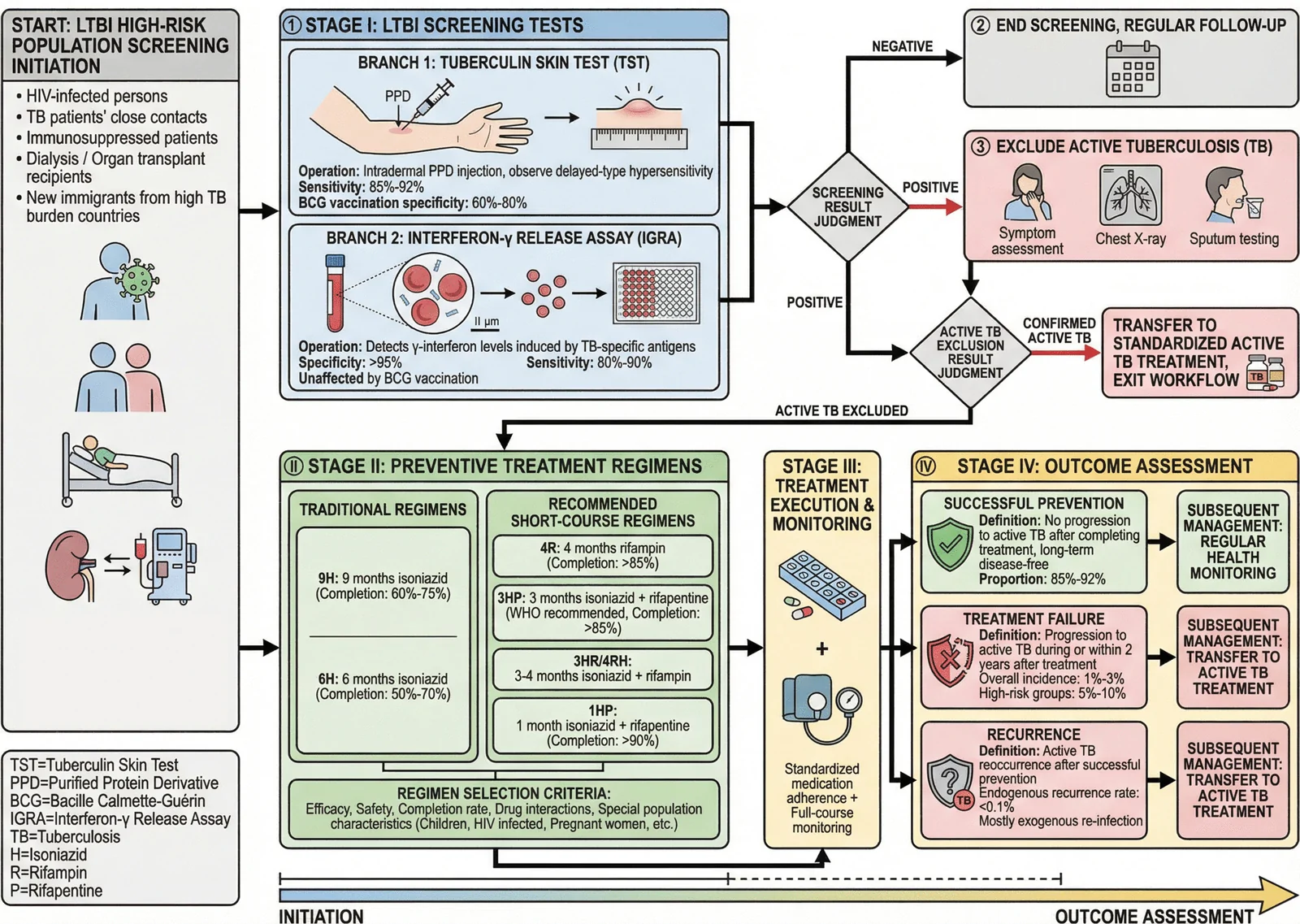
Standardized clinical workflow for latent tuberculosis infection management: screening, treatment and outcome assessment. Standardized clinical workflow for latent tuberculosis infection (LTBI) management. Stage I: High-risk populations screened via tuberculin skin test (TST) or interferon-gamma release assay (IGRA). Stage II: Positive screens followed by imaging, sputum testing and clinical assessment to rule out active TB. Stage III: Eligible patients receive standardized short-course (3HP, 4R) or long-course isoniazid regimens. Stage IV: Regular monitoring evaluates completion rate, adverse events and long-term protective efficacy

The major challenges in current LTBI treatment lie in its prolonged treatment course (typically 6–9 months) and frequent adverse events, which collectively result in suboptimal patient adherence. Short-course preventive regimens, such as 3HP (weekly isoniazid plus rifapentine for 3 months) and 3HR (daily isoniazid plus rifampicin for 3 months), have exhibited higher completion rates and more favorable safety profiles relative to conventional long-course regimens. However, adherence constraints persist even for 3-month regimens, highlighting the development of long-acting formulations as a pivotal direction for future clinical translation. Micro-nano emulsions and nanocarrier systems enable sustained release of antituberculosis agents, thereby reducing dosing frequency. Long-acting injectable formulations, such as monthly or quarterly subcutaneous dosage forms drawing on successful experience in cancer therapy and HIV prevention, are expected to fundamentally reshape the treatment paradigm of LTBI. For clinical translation, priority should be given to characterizing the pharmacokinetic properties, long-term safety, and large-scale manufacturing feasibility of these novel formulations in humans, with particular focus on optimizing their accessibility in resource-poor settings. The standardized clinical workflow for latent tuberculosis infection management, from high-risk population screening and active disease exclusion to preventive treatment and outcome assessment, is presented in Fig. 11. The clinical development pipeline of tuberculosis vaccine candidates across different technology platforms and indication types is presented in Table 2.

**Table 2:** Representative next-generation tuberculosis vaccine candidates in phases I–III clinical trials

Vaccine name	Mechanism of action/technology platform	Core NCT registration number
Phase III Clinical		
M72/AS01E	Mtb32A + Mtb39A fusion protein combined with AS01E adjuvant induces strong CD4 + T cell immunity for preventing disease in latently infected population	NCT06062238
MTBVAC	Attenuated human-type Mycobacterium tuberculosis with knockout of phoP and fadD26 virulence genes, retaining more natural T cell epitopes, covering newborns and adults	NCT04975178
VPM1002	Replacement of urease C gene with listeriolysin O gene promotes antigen cytosolic delivery and enhances immunogenicity	NCT04351685
GamTBvac	Ag85A + ESAT-6 antigen combination with adjuvant for adult prevention	NCT04975737
Phase II Clinical		
BCG Adolescent/Adult Revaccination	Enhanced long-term protection, reducing disease risk in adolescents/adults	NCT04152161
Phase I Clinical		
BNT164a1/BNT164b1	mRNA-LNP platform encoding 8 Mtb antigens covering different stages of infection, with differential nucleoside modification	NCT05537038
H107e/CAF®10b (nTB-01)	Novel antigen combined with CAF10b adjuvant, first-in-human trial	NCT06050356
TB/FLU-04L/TB/FLU-05E	Intranasal administration, expressing TB10.4, HspX and other antigens, mucosal immunity-oriented	NCT02501421
Ad5 Ag85A	Adenovirus type 5 vector expressing Ag85A antigen, enhancing cellular immunity	NCT02337270
AERAS-404	Ag85B + TB10.4 fusion protein combined with IC31 adjuvant for booster immunization after BCG priming	NCT02420444
ID93 + GLA-SE (QTP101)	4 Mtb antigens (virulence + latency-associated) combined with GLA-SE adjuvant (TLR4 agonist)	NCT06714513
RUTI	Inactivated Mycobacterium tuberculosis fragment preparation	NCT00546273
H56:IC31	Ag85B-ESAT-6-Rv2660c fusion protein combined with IC31 adjuvant, covering active and dormant bacteria	NCT02503839

## Future outlook and conclusions

Over the past five years, anti-tuberculosis research has achieved landmark progress across mechanistic dissection, drug development, clinical application and preventive intervention, laying a solid foundation for advancing toward the WHO End TB Strategy. Nevertheless, the global tuberculosis control landscape still faces multifaceted challenges, including community transmission of drug-resistant strains, clinical translation gaps of novel technologies, and insufficient adaptation for special populations. This concluding section outlines five priority strategic directions for future research and public health practice, spanning mechanistic deepening, clinical translation acceleration, precision medicine system construction, prevention system upgrading and global collaborative capacity building, providing a translational roadmap toward a tuberculosis-free world.

During 2020–2025, the field of antituberculosis treatment has achieved breakthrough progress in multiple dimensions including mechanism research, drug development, clinical application, and prevention and control technologies, bringing profound changes to the global TB prevention and control landscape. At the mechanistic research level, there has been in-depth analysis of the action targets and resistance mechanisms of classic drugs such as bedaquiline and delamanid, clarification of resistance mechanisms mediated by drug-resistant mutations, and successful identification of a series of emerging targets including cell wall biosynthesis, enzyme complex regulation, and immune checkpoints, laying a solid foundation for the development of novel drugs.

In terms of drug development and clinical translation, breakthroughs in short-course treatment regimens are particularly remarkable. Regimens such as BPaL/BPaLM have shortened the treatment duration of MDR-TB from 18–24 months to 6–9 months, and original short-course regimens have shown excellent efficacy in scenarios such as drug-susceptible TB and extensively drug-resistant TB; targeted delivery systems and personalized treatment strategies for TB at special sites such as spinal TB and tuberculous meningitis have been continuously improved, significantly reducing the incidence of severe complications. In the field of drug-resistant TB prevention and control, technologies such as whole-genome sequencing have revealed the spatiotemporal evolution of resistance mechanisms, including multi-dimensional mechanisms such as classic resistance escalation, novel resistance pathways, and immune escape resistance, and precision treatment regimens based on resistance stratification have been constructed, effectively improving the treatment success rate of MDR-TB and pre-XDR/XDR-TB.

Precision medicine and biotherapy have become important growth points. Dose optimization guided by pharmacogenomics and PK/PD models, as well as AI-assisted diagnosis and regimen recommendation, have promoted antituberculosis treatment into the era of personalization; exploration of novel biotherapies such as CAR-T cells, MSCs therapy, small nucleic acid drugs, and phage therapy has provided new approaches to overcoming drug resistance and eliminating dormant bacteria. Vaccine development and preventive intervention technologies have advanced simultaneously. Therapeutic vaccines such as multi-stage protein subunit vaccines, viral vector vaccines, and nucleic acid vaccines have entered clinical stages, and the application of digital prevention tools has significantly improved treatment adherence and prevention and control efficiency.

However, global TB prevention and control still faces multiple challenges: community transmission of drug-resistant TB has not been effectively curbed, and there is still an unmet need for XDR-TB treatment; treatment adaptability for special populations (immunocompromised individuals, children, patients with comorbidities) is insufficient; the prevention-treatment linkage gap caused by the limited protective efficacy of BCG against adult pulmonary TB still exists; clinical translation of novel biotherapies faces technical bottlenecks and safety assessment pressures. These issues need to be continuously addressed through interdisciplinary collaboration. 

In-depth expansion of mechanism research: In the future, continuous focus should be placed on the survival strategies and resistance mechanisms of *Mycobacterium tuberculosis*, with emphasis on analyzing the metabolic characteristics and awakening pathways of dormant bacteria, exploring the druggable potential of more "undruggable" targets; in-depth investigation of novel drug resistance regulatory mechanisms such as non-coding RNAs and epigenetic modifications, as well as the interaction between gut microbiota and *Mycobacterium tuberculosis*, to provide theoretical support for the development of innovative therapies targeting dormant bacteria and reversing drug resistance. At the same time, it is necessary to strengthen research on host–pathogen interaction mechanisms, clarify the key molecular pathways of immune escape, and provide new targets for the optimization of host-directed therapy.

Accelerated clinical translation of drugs and therapeutics: In terms of drug development, it is necessary to advance clinical trials of late-stage investigational drugs such as next-generation diarylquinolines and DprE1 inhibitors, optimize dosing regimens and combination strategies; for refractory cases such as XDR-TB, develop multi-target combination therapies and drug repurposing schemes to improve treatment success rates. In the field of delivery systems, further improve the performance of bone-targeted, brain-targeted, and lesion-responsive nanoformulations, solve issues related to biocompatibility, in vivo stability, and targeting precision, and promote the efficacy upgrade of treatment for TB at special sites. Novel biotherapies need to break through technical bottlenecks, optimize the targeting and safety of CAR-T cells and phage cocktail formulations, conduct larger-scale clinical trials to verify their effectiveness, and explore the synergistic effects of TPD technology with traditional antituberculosis drugs to shorten treatment duration.

Comprehensive construction of precision medicine system: Relying on multi-omics technologies (genomics, transcriptomics, proteomics, metabolomics) and AI algorithms, establish more accurate drug resistance prediction models and efficacy evaluation systems to achieve full-process personalized guidance from drug resistance screening, regimen formulation to efficacy monitoring. Promote the Model-Informed Precision Dosing strategy, combined with Therapeutic Drug Monitoring and PBPK models, to provide customized dose regimens for special populations. Deepen the application of AI in diagnosis and treatment, develop intelligent diagnosis and treatment platforms integrating imaging, laboratory data, and clinical information, realize early screening, precise diagnosis of TB, and dynamic optimization of treatment regimens, and especially improve the accessibility of technologies in resource-poor areas.

System upgrade of vaccines and preventive interventions: Vaccine development should focus on the dual goals of "prevention + treatment", promote the combined application of therapeutic vaccines and novel preventive vaccines, and improve the intervention effect on latent TB infection; Optimize the delivery system of mucosal vaccines, enhance local respiratory immune responses, and improve the protective efficacy of vaccines against adult pulmonary TB. In terms of preventive interventions, expand the scope of precision screening for LTBI, develop long-acting preventive treatment drugs to achieve the prevention and control goal of "one-shot cure"; further promote digital tools (mobile health applications, remote monitoring platforms), build a full-chain prevention and control system covering high-risk population screening, treatment adherence management, and epidemic monitoring, and narrow the regional gap in global prevention and control.

**Fig. 12:**
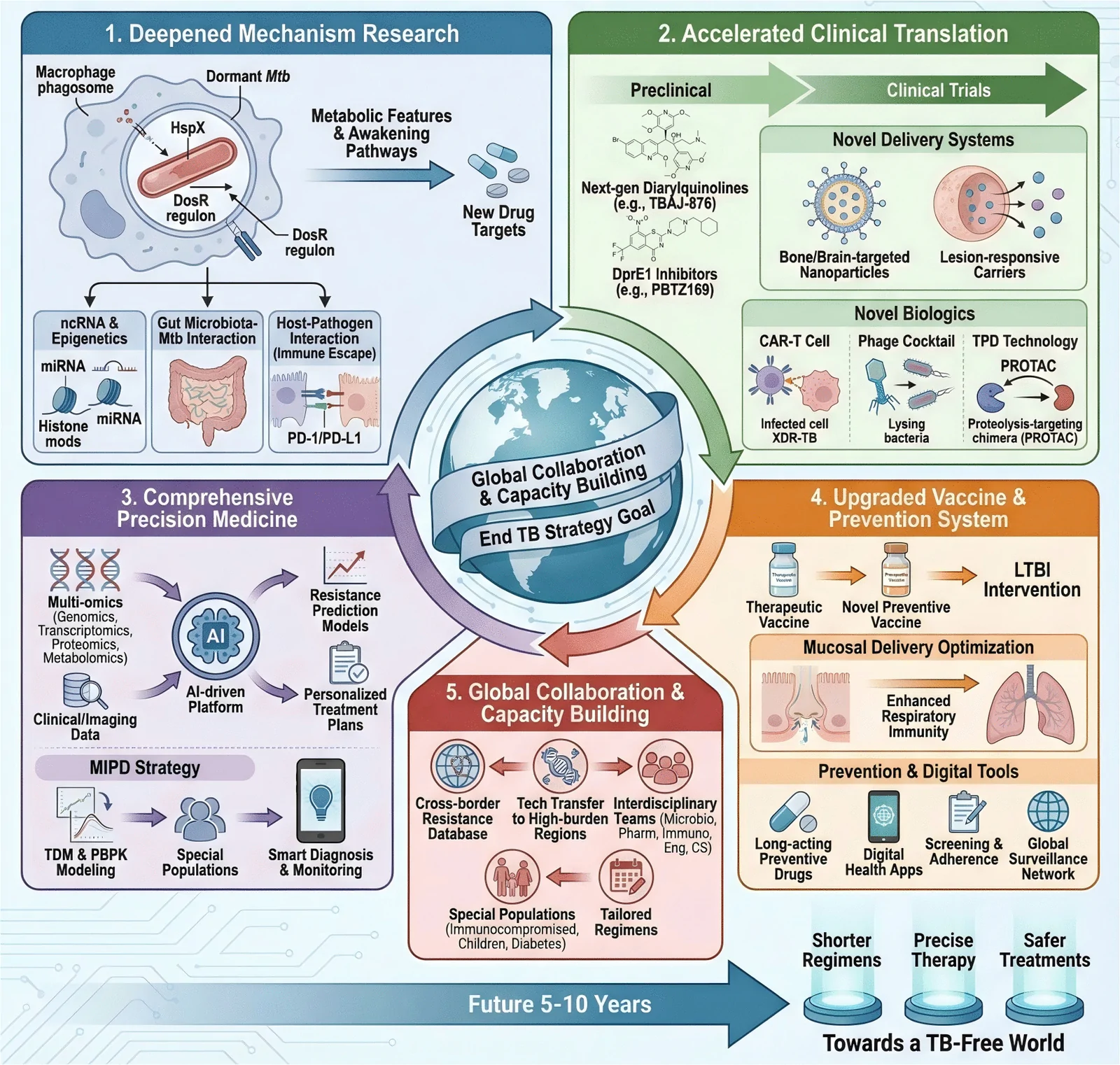
Priority strategic directions for tuberculosis research and global control toward WHO End TB goals. Five priority strategic directions for TB research and control toward WHO End TB goals. 1: Dissecting dormant Mtb biology, non-coding/epigenetic/microbiota resistance mechanisms, and novel host–pathogen targets. 2: Advancing novel agent trials; developing targeted nano delivery systems; optimizing biotherapy translational pathways. 3: Integrated multi-omics/AI precision platforms for individualized treatment and dynamic disease management. 4: Next-generation mucosal vaccines; expanded LTBI intervention; digital health tools for improved adherence. 5: Transnational resistance surveillance; equitable drug/vaccine access; capacity building in high-burden regions

Global collaboration and capacity building for prevention and control: China, as a high TB-burden country, has built a comprehensive multi-level TB prevention and control system featuring designated hospital networks and the "three-in-one" collaborative model involving centers for disease control, designated hospitals, and primary healthcare institutions, which has been recognized by the WHO as a reference model for resource-limited settings. National TB control programs and public health investment have driven substantial reductions in TB incidence and mortality over the past two decades, providing real-world evidence for global TB control strategy optimization. South-South cooperation and multilateral initiatives such as the Global Fund facilitate technology transfer and pharmaceutical support to high-burden countries along the Belt and Road, strengthening global health governance for TB. Strengthening transnational surveillance of drug-resistant strains, establishing shared genomic databases, and promoting equitable access to novel drugs and vaccines will be core priorities for global collaboration in the coming decade. Five priority strategic directions for advancing tuberculosis research and public health control toward the WHO End TB goals over the next 5–10 years are outlined in Fig. 12.

In the next 5–10 years, with the deepening of mechanism research, transformation of innovative technologies, and improvement of the global prevention and control system, TB treatment will gradually enter an era of "short-course, precision, and harmlessness", the epidemic of drug-resistant TB will be effectively curbed, and the goal of the "End TB Strategy" is expected to be gradually achieved. This requires the continuous efforts and close collaboration of global researchers, clinicians, public health personnel, and policymakers to jointly address this global public health challenge.

## Data Availability

All data supporting the findings of this study are available within the article and its cited references. No original datasets were generated for this review.
